# SuperNatural inhibitors to reverse multidrug resistance emerged by ABCB1 transporter: Database mining, lipid-mediated molecular dynamics, and pharmacokinetics study

**DOI:** 10.1371/journal.pone.0288919

**Published:** 2023-07-26

**Authors:** Mahmoud A. A. Ibrahim, Khlood A. A. Abdeljawaad, Alaa H. M. Abdelrahman, Mahmoud M. H. Abdelhamid, Mohamed Ahmed Naeem, Gamal A. H. Mekhemer, Peter A. Sidhom, Shaban R. M. Sayed, Paul W. Paré, Mohamed-Elamir F. Hegazy

**Affiliations:** 1 Computational Chemistry Laboratory, Chemistry Department, Faculty of Science, Minia University, Minia, Egypt; 2 School of Health Sciences, University of KwaZulu-Natal, Durban, South Africa; 3 Nutrition and Food Sciences, Ain Shams University Specialized Hospital, Ain Shams University, Cairo, Egypt; 4 Department of Pharmaceutical Chemistry, Faculty of Pharmacy, Tanta University, Tanta, Egypt; 5 Department of Botany and Microbiology, College of Science, King Saud University, Riyadh, Saudi Arabia; 6 Department of Chemistry & Biochemistry, Texas Tech University, Lubbock, Texas, United States of America; 7 Chemistry of Medicinal Plants Department, National Research Centre, Giza, Egypt; Nazarbayev University School of Medicine, PAKISTAN

## Abstract

An effective approach to reverse multidrug resistance (MDR) is P-glycoprotein (P-gp, ABCB1) transport inhibition. To identify such molecular regulators, the SuperNatural II database, which comprises > 326,000 compounds, was virtually screened for ABCB1 transporter inhibitors. The Lipinski rule was utilized to initially screen the SuperNatural II database, identifying 128,126 compounds. Those natural compounds were docked against the ABCB1 transporter, and those with docking scores less than zosuquidar (ZQU) inhibitor were subjected to molecular dynamics (MD) simulations. Based on MM-GBA binding energy (Δ*G*_binding_) estimations, UMHSN00009999 and UMHSN00097206 demonstrated Δ*G*_binding_ values of –68.3 and –64.1 kcal/mol, respectively, compared to ZQU with a Δ*G*_binding_ value of –49.8 kcal/mol. For an investigation of stability, structural and energetic analyses for UMHSN00009999- and UMHSN00097206-ABCB1 complexes were performed and proved the high steadiness of these complexes throughout 100 ns MD simulations. Pharmacokinetic properties of the identified compounds were also predicted. To mimic the physiological conditions, MD simulations in POPC membrane surroundings were applied to the UMHSN00009999- and UMHSN00097206-ABCB1 complexes. These results demonstrated that UMHSN00009999 and UMHSN00097206 are promising ABCB1 inhibitors for reversing MDR in cancer and warrant additional *in-vitro*/*in-vivo* studies.

## Introduction

Carcinoma is often an incurable disease, with approximately two million cases reported in 2020 alone [1]. Chemotherapy is one of the most widely used treatments [2], even though remission is observed in only 10% of cancer cases because neoplasm cells rapidly evolve resistant to most anticarcinoma drugs. Tumor cell excretion of chemotherapeutic medications is greatly aided by ATP-binding cassettes (ABC) and other membrane proteins that are powered by ATP hydrolysis [3, 4]. P-glycoprotein (P-gp), also known as ABCB1 (ATP-binding cassette), is among the most effective ABC proteins for drug export [5]. ABCB1 transporter has been recognized as a key component in the emergence of MDR tumor lines [6]. The ABCB1 transporter is encoded by an MDR1 gene having a molecular weight of 170 kilodaltons [7]. The ABCB1 transporter is observed to be expressed at high levels in colorectal cancer [8]. Two nucleotide-binding domains (NBDs) and two transmembrane domains (TMDs) work jointly for ABCB1 activation [9]. ABCB1 is activated when a substrate (Oncodrugs) binds to the protein TMD. A closed dimer is formed, and ATP binds to the two NBDs. ATP hydrolysis catalyzes a conformation change in the protein with the bound drug pumped out of the cell [10–13]. Therapeutic tumor treatments are anticipated with the discovery of new/effective ABCB1 blockers or modulators [16]. Indeed, several generations of ABCB1 blockers have been discovered using combinatorial chemistry [14, 15]. Unfortunately, such inhibitors have been ineffective due to high general toxicity and other complications facing cancer patients [16, 17]. Therefore, screening of new inhibitors against ABCB1 transporter is essential to reverse the MDR process.

**Fig 1:**
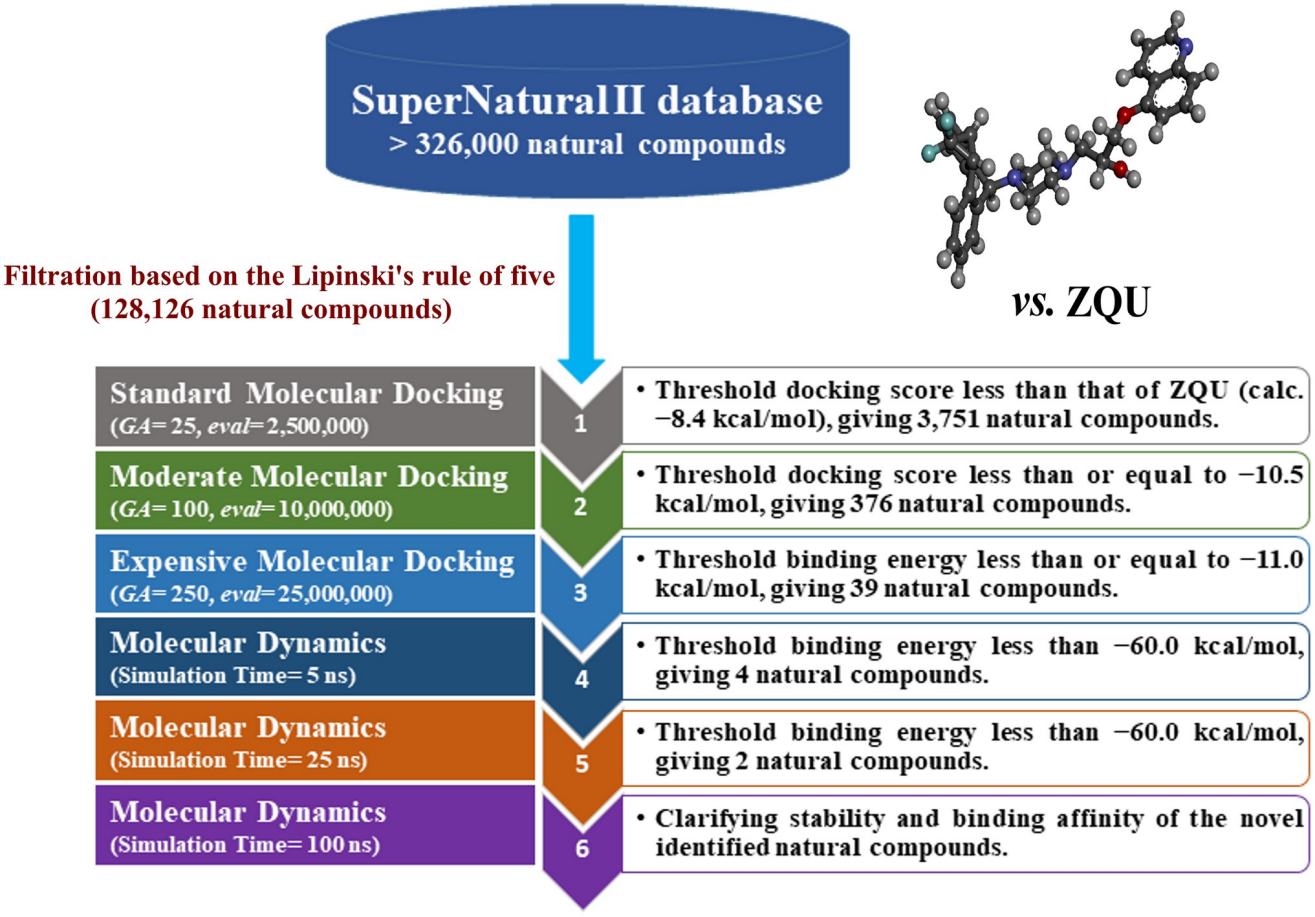
Schematic diagram of the *in-silico* techniques and filtration of the SuperNatural II database.

Natural products (NPs) have been deemed as an essential source of pharmaceutical drugs [18]. Several vastly-utilized anticarcinoma therapeutics have been obtained from natural sources, including paclitaxel, vincristine, etoposide, and irinotecan [19]. SuperNatural II database provides a classification based on the characterization of structures like alkaloids, amino acids, or fatty acids. The SuperNatural II database contains > 326,000 compounds, which are compiled from 16 suppliers [20–24]. This study was set to discover effective compounds from the SuperNatural II database that can inhibit the ABCB1 transporter using *in-silico* approaches. Thus, virtual screening, docking computations, and MD simulations were performed. Additionally, the binding affinities of the SuperNatural II inhibitors against the ABCB1 transporter were estimated employing the MM-GBSA approach. Stabilization of the structure and energy of the predicted natural inhibitors complexed with the ABCB1 transporter were examined over 100 ns MD simulations. Additionally, the identified SuperNatural II compounds complexed with ABCB1 transporter were simulated in POPC membrane to mimic physiologic circumstances. A schematic diagram for the utilized *in-silico* methods during the filtration of the database is represented in Fig 1. The pharmacokinetic characteristics of the identified ABCB1 inhibitors were predicted. The current work may be useful as a good starting point for identifying potent inhibitors against the ABCB1 transporter to reverse MDR.

## Computational methods

### ABCB1 preparation

The RCSB PDB database was used to retrieve the structure of the ABCB1 transporter [25]. The 3d structure of the ABCB1 transporter complexed with the zosuquidar ((2*R*)-1-(4-((1a*R*,10b*S*)-1,1-difluoro-1,1a,6,10b-tetrahydrodibenzo[a,e]cyclopropa[c][7]annulen-6-yl)piperazin-1-yl)-3-(quinolin-5-yloxy)propan-2-ol) (ZQU) inhibitor (PDB ID: 6QEE, resolution: 3.90 Å [26]) was used for all *in-silico* approaches. Modeller software was utilized to build the missing residues [27]. Preparing the protein involved assigning bonds in the correct order, orienting disoriented groups, and removing co-crystalized water molecules and inhibitors. The protonation states of residues were determined, and the missing hydrogens were inserted using the H++ web server [28].

### SuperNatural II preparation

The SuperNatural II database containing > 326,000 compounds was obtained in SDF format from Prof. Encinar’s website (http://docking.umh.es/downloaddb) [29]. All compounds were converted into 3D with the aid of Omega2 software, with a maximum of 200 conformers generated within a 10 kcal/mol energy window [30, 31]. All generated 3D structures were energetically minimized using MMFF94S force field within SZYBKI software [32, 33]. To study the protonation states of the database compounds, the fixpk_a_ implemented within QUACPAC software was applied [34]. The Gasteiger method was applied to assign the charges of the database compounds [35]. The prepared database is available via www.compchem.net/ccdb.

### Database filtration

The database was first filtered on the basis of Lipinski’s rule of five [29]. The filtration criteria were as follows: i) nOHNH (number of hydrogen bond donors) was appointed to ≤ 5, ii) nON (number of hydrogen bond acceptors) was set to ≤ 10, iii) MWt (molecular weight) of each compound was selected to be ≤ 500, iv), TPSA (topological polar surface area) was assigned to be ≤ 140, and v) Log*P* (partition coefficient) was appointed to ≤ 5. All compounds that obey Lipinski’s rule of five were then subjected to *in-silico* computation to evaluate their potency as ABCB1 inhibitors.

### Molecular docking

Molecular docking computations were carried out using AutoDock4.2.6 software [36]. The pdbqt file for the ABCB1 transporter was generated using MGL tools [37]. The receptor was kept rigid for docking. All docking parameters were set to default, with an exception to the *GA* (number of genetic algorithms run) and *eval* (maximum number of energy evaluations) values. Three docking levels were used, named standard, moderate, and expensive docking computations, with *GA* and *eval* values of 25 and 2,500,000, 100 and 10,000,000, and 250 and 25,000,000, respectively. The binding pocket for the ABCB1 transporter was specified by a grid box with 60 Å × 60 Å × 60 Å in the 3-dimensions with a spacing value of 0.375 Å. The built-in clustering analysis was used to group the anticipated docking poses for each compound using an RMSD tolerance of 1.0 Å.

### Molecular dynamics (MD)

MD simulations were executed with the assistance of AMBER16 software [38]. Detailed information about the applied MD protocol is described elsewhere [39–42]. Briefly, GAFF2 (general AMBER force field) was employed for the ligand parameterization [43]. On the other hand, ABCB1 transporter was characterized by AMBER force field 14SB [44]. RESP (restrained electrostatic potential) fitting approach was utilized to compute the charges of the inspected compounds at the HF/6-31G* level employing Gaussian09 software [45, 46]. For complex preparation, all inhibitor-ABCB1 complexes were centered in an octahedral box with a distance of 12 Å between the edge of the box and atoms of the inhibitor-ABCB1 complexes and solvated with TIP3P water molecules [47]. Charge neutralization was performed by inserting sodium and chloride counterions. Besides, a salt concentration was adjusted at 0.15 M NaCl. Subsequently, the prepared complexes were submitted to energetical minimization for 5,000 cycles. The minimized complexes were then heated up to 310 K under NVT ensemble. Heating stages were executed through 50 ps. The equilibration phase was then executed over 10 ns. Production stages were performed using NPT ensemble for 5, 25 and 100 ns. Berendsen barostat and Langevin dynamics were employed to control both pressure and temperature, respectively [48, 49]. The trajectories were recorded every 10 ps for structural and energetical analyses. Molecular visualizations were performed with BIOVIA discovery studio visualizer [50].

### MD simulations in the lipid bilayer

CHARMM-GUI web interface was used to construct ABCB1-lipid bilayer systems [51]. The ABCB1 transporter was embedded in a 1-palmitoyl-2-oleoyl-phosphatidylcholine (POPC) bilayer with a preset TIP3P solvent was used for computations. Cl^−^and Na^+^ ions were employed to neutralize the systems under investigation. Furthermore, the inhibitor-ABCB1 systems were positioned in the middle of the POPC membrane. The Lipid14 force field, implemented within the AMBER16 software, was utilized to describe the POPC membrane [52]. Ultimately, the MD simulations regarding the constructed systems were carried out using the same computational framework discussed in Section 2.5.

### Binding energy computations

Binding energy calculations for compounds in complex with ABCB1 transporter were estimated using an MM-GBSA (molecular mechanical-generalized Born surface area) approach [53]. The MM-GBSA approach was evaluated on the basis of uncorrelated snapshots gathered throughout the MD simulations. Herein, a modified generalized Born (GB) model proposed by Onufriev was utilized [54]. The Δ*G*_binding_ was computed according to the following equation:

$${\Delta G}_{binding}=G_{Complex}-\left(G_{Inhibitor}+G_{ABCB1}\right)$$ (1)

where the *G* term is:

$$G=G_{GB}+G_{SA}+E_{ele}+E_{vdW}$$ (2)

*E*_ele_, *E*_vdW_, *G*_GB_, and *G*_SA_ stand for electrostatic, van der Waals, electrostatic-solvation free energy, and nonpolar solvation-free energy, respectively. The entropy (*S*) evaluation was ignored because of its high computation need [55, 56]. In the lipid bilayer, the POPC was preserved in the MM-GBSA estimations over the MD simulations. All *in-silico* computations in this study were executed using the hybrid cluster of CompChem GPU/CPU (hpc.compchem.net).

### Pharmacokinetic characteristics

The pkCSM was employed to estimate the pharmacokinetic characteristics, involving absorption, distribution, metabolism, excretion, and toxicity (ADMET) [57]. The Caco-2 permeability and human intestine absorption (HIA) were utilized to predict absorption (A). Furthermore, the distribution (D) property was clarified via a steady-state volume distribution (VDss). A metabolism (M) property was predicted on the basis of CYP3A4 inhibitor, and the excretion (E) property was estimated based on the drug’s total clearance. AMES toxicity was employed to evaluate the toxicity (T) property of the investigated inhibitors.

## Results and discussion

### SuperNatural II database pre-screening

Prior to virtual screening against the ABCB1 transporter, the database was initially filtrated based on Lipinski’s rule of five. Only 128,126 out of > 326,000 natural compounds followed Lipinski’s rules. Thus, these compounds were prepared and screened against the ABCB1 transporter.

### Virtual screening of the SuperNatural II database

The outperformance of AutoDock4.2.6 software in predicting the ZQU-ABCB1 binding pose has been previously reported [39]. According to the published results, the calculated RMSD value between the predicted and experimental binding modes was 0.18 Å, with a docking score of −8.3 kcal/mol. Utilizing the validated docking protocol, the 128,126 pre-screened compounds were virtually screened towards the ABCB1 transporter utilizing standard parameters. According to the standard docking scores, 3,751 natural compounds demonstrated docking scores less than ZQU (calc. −8.4 kcal/mol against the ABCB1 transporter). Accordingly, these 3,751 compounds were re-docked against the ABCB1 transporter utilizing moderate docking parameters. The predicted docking scores for these 3,751 compounds (S1 Table). According to results listed in S1 Table, 376 natural compounds demonstrated docking scores ≤ –10.5 kcal/mol. Thus, these 376 natural compounds were subjected to more expensive computations. The corresponding docking scores are summarized in S2 Table. Based on data listed in S2 Table, only 39 natural compounds revealed docking scores ≤ –11.0 kcal/mol. Notably, the docking scores of –10.5 and –11.0 kcal/mol were selected as threshold values to shortlist the most potent inhibitors. The anticipated docking scores of these 39 natural compounds are compiled in Table 1. As well, the 2-dimensional interactions of these natural compounds with key ABCB1 binding residues are depicted in S1 Fig.

**Table 1:** Standard, moderate, and expensive docking scores of the top potent 39 SuperNatural II compounds against the ABCB1 transporter^a^.

SuperNatural II Code	Docking Score (kcal/mol)			SuperNatural II Code	Docking Score (kcal/mol)		
	Std.^b^	Mod.^c^	Exp.^d^		Std.^b^	Mod.^c^	Exp.^d^
**ZQU**	–8.4	–8.2	–8.3	**UMHSN00081081**	–10.4	–10.6	–12.1
**UMHSN00009999**	–12.5	–13.1	–12.6	**UMHSN00302140**	–12.1	–11.6	–12.1
**UMHSN00097206**	–12.5	–11.5	–12.5	**UMHSN00080936**	–11.9	–11.9	–11.9
**UMHSN00062899**	–12.1	–12.4	–12.4	**UMHSN00011720**	–12.7	–11.6	–11.9
**UMHSN00066079**	–12.4	–12.4	–12.4	**UMHSN00380932**	–11.6	–11.8	–11.8
**UMHSN00008763**	–10.4	–11.9	–12.1	**UMHSN00059546**	–10.8	–11.6	–11.8
**UMHSN00317150**	–11.8	–11.8	–11.8	**UMHSN00081079**	–11.7	–11.7	–11.7
**UMHSN00081067**	–11.8	–11.6	–11.8	**UMHSN00081043**	–11.6	–11.6	–11.6
**UMHSN00337215**	–11.8	–11.8	–11.8	**UMHSN00004668**	–11.0	–11.6	–11.6
**UMHSN00080939**	–11.8	–11.8	–11.8	**UMHSN00008381**	–10.0	–11.4	–11.6
**UMHSN00089274**	–11.5	–11.8	–11.8	**UMHSN00009954**	–11.4	–11.5	–11.6
**UMHSN00058310**	–10.5	–11.4	–11.8	**UMHSN00054684**	–11.4	–11.5	–11.5
**UMHSN00054807**	–11.1	–11.6	–11.6	**UMHSN00265972**	–11.5	–11.5	–11.5
**UMHSN00007945**	–10.2	–11.5	–11.5	**UMHSN00377659**	–11.4	–11.4	–11.4
**UMHSN00079222**	–11.2	–11.8	–11.5	**UMHSN00139755**	–9.7	–11.4	–11.4
**UMHSN00360652**	–11.5	–11.4	–11.5	**UMHSN00010807**	–11.3	–11.8	–11.4
**UMHSN00009897**	–11.2	–12.6	–11.5	**UMHSN00062975**	–11.5	–11.5	–11.4
**UMHSN00084553**	–11.1	–11.8	–11.5	**UMHSN00050809**	–9.9	–11.0	–11.3
**UMHSN00260998**	–10.9	–12.1	–11.5	**UMHSN00427183**	–10.9	–12.8	–11.2
**UMHSN00249525**	–11.4	–11.4	–11.4	**UMHSN00079819**	–10.3	–11.5	–11.1

^a^Data ranked based on the expensive docking calculations.

^b^Std. refers to standard docking calculations.

^c^Mod. refers to moderate docking calculations.

^d^Exp. refers to expensive docking calculations.

**Fig 2:**
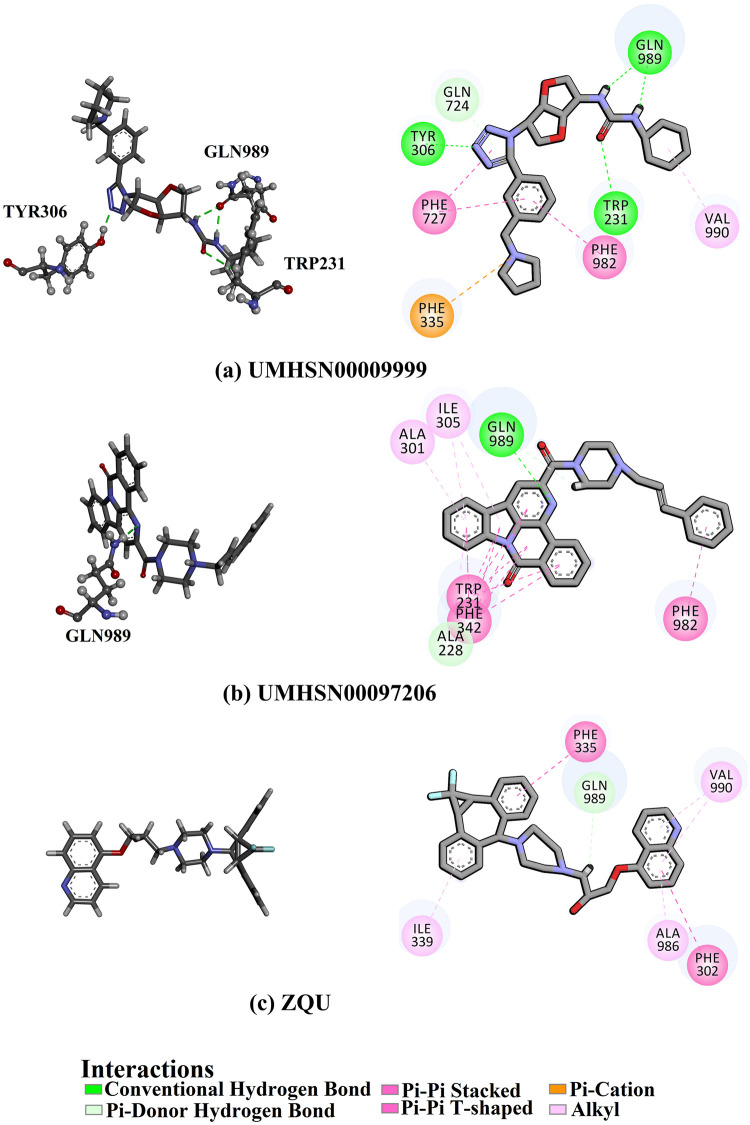
3-Dimensional and 2-Dimensional interactions of (a) UMHSN00009999, (b) UMHSN00097206, and (c) ZQU with the ABCB1 transporter.

Compound UMHSN00009999 manifested the lowest docking score with a value of –12.6 kcal/mol against the ABCB1 transporter (Table 1). Examining its docking mode demonstrated that the nitrogen atom of the 1,5-dimethyl-1H-tetrazole ring formed a hydrogen bond with TYR306 residue (1.73 Å) (Fig 2). Dimethylamine groups of the 1,3-dimethylurea formed two hydrogen bonds with the oxygen atom of the acetamide group of the GLN989 residue with lengths of 2.11 and 2.06 Å, respectively (Fig 2). The oxygen atom of the 1,3-dimethylurea contributed to another bond with the 1H-indole group of TRP231 residue (2.76 Å). Besides, compound UMHSN00009999 interacted with PHE727 and PHE981 residues by pi-pi interactions (Fig 2).

Compound UMHSN00097206 showed the second-lowest ABCB1 docking score with a value of –12.5 kcal/mol. Investigating the docking mode of UMHSN00097206 demonstrated that the nitrogen atom of the 6-methyl-1,5-naphthyridin-2(1H)-one participated in a hydrogen bond with GLN989 residue (2.16 Å). Besides, compound UMHSN00097206 displayed pi-pi interactions with TRP231, PHE342, and PHE982 residues of the ABCB1 binding pocket (Fig 2). ZQU did not exhibit any hydrogen bond inside the ABCB1 binding pocket, so the relatively strong docking score of –8.4 kcal/mol could be assigned to pi-pi interactions with PHE302 and PHE335 residues.

### Molecular dynamics (MD)

**Fig 3:**
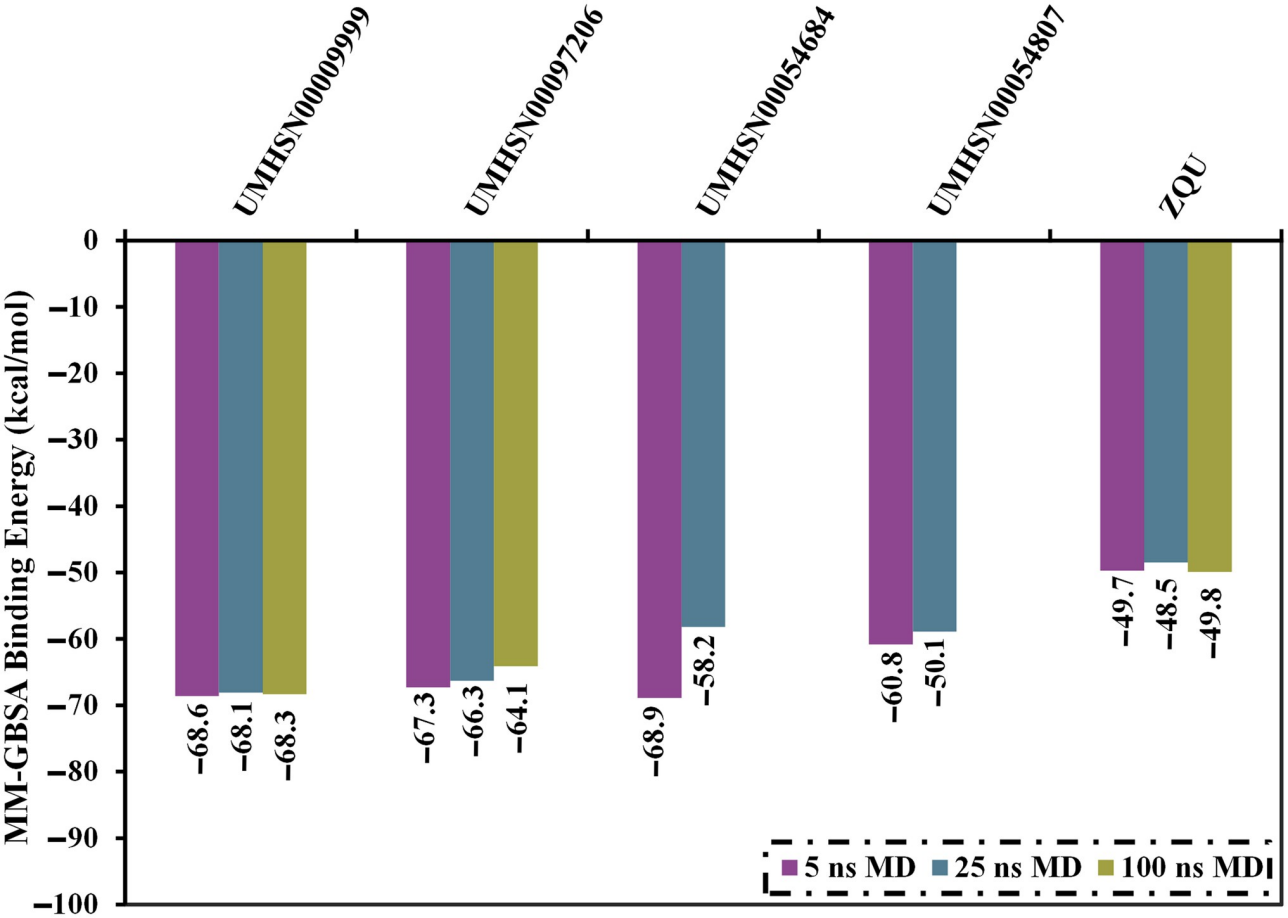
Calculated binding energies (in kcal/mol) throughout 5, 25, and 100 ns MD simulations for the promising SuperNatural II compounds against the ABCB1 transporter.

To verify the docking results and investigate the stability of identified compounds within the ABCB1 binding pocket, MD simulations combined with binding energy computations were executed. The top 39 compounds that showed ABCB1 docking scores ≤ –11.0 kcal/mol were submitted to MD simulations over 5 ns, pursued by estimating the binding energy utilizing MM-GBSA approach (S3 Table). Based on the evaluated MM-GBSA energies, only four compounds exhibited outstanding binding energy (Δ*G*_binding_) lower than –60.0 kcal/mol. Consequently, 25 ns MD simulations were conducted for these compounds to establish more reliable binding affinities within the binding pocket. Strikingly, out of the four identified natural compounds as prospective ABCB1 inhibitors, UMHSN00097206 and UMHSN00009999 demonstrated constancy binding energies with Δ*G*_binding_ values of −68.6 and −68.1 and −67.3 and −66.3 kcal/mol over 5 ns and 25 ns MD course, respectively (Fig 3). However, UMHSN00054807 and UMHSN00054684 complexed with ABCB1 transporter displayed a significant increase with an approximate value of –10.0 kcal/mol in the Δ*G*_binding_ over 25 ns, with respect to the Δ*G*_binding_ over 5 ns MD simulations (Fig 3). For this reason, UMHSN00054807 and UMHSN00054684 were excluded from the data set.

MD simulations were then extended to 100 ns for UMHSN00097206 and UMHSN00009999 complexed with ABCB1 to improve the predicted reliability. The estimated MM-GBSA binding energies over this extended MD simulation (Fig 3).

No variances between the estimated binding energies during the 25 and 100 ns MD simulations for UMHSN00009999, UMHSN00097206, and ZQU were observed (Fig 3). Compared to ZQU (calc. –49.8 kcal/mol), UMHSN00009999 and UMHSN0097206 showed superior ABCB1 binding affinities during the 100 ns MD simulations with average Δ*G*_binding_ values of –68.3 and –64.1 kcal/mol, respectively (Fig 3).

**Fig 4:**
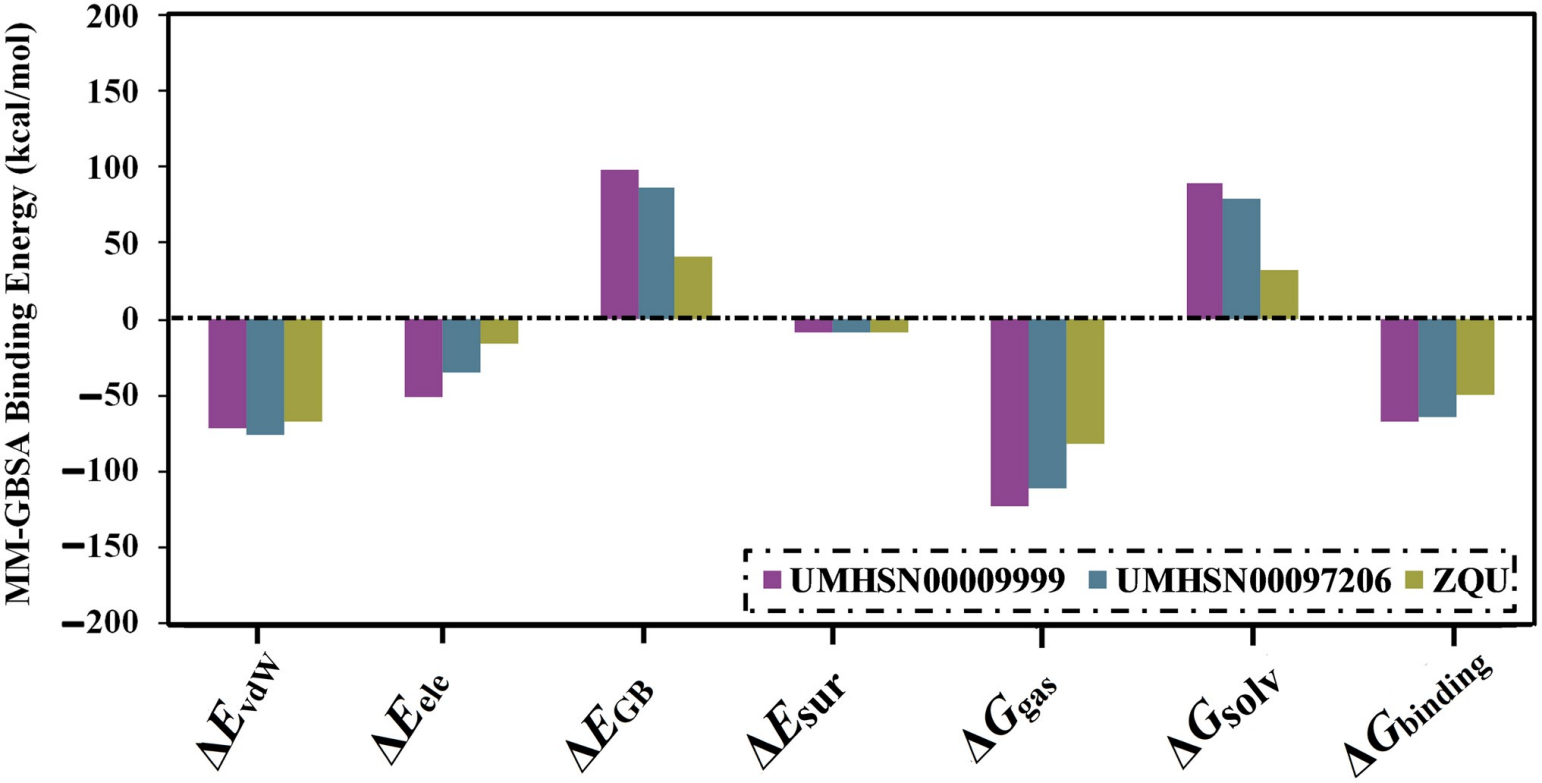
Decomposition of binding energies of UMHSN00009999, UMHSN00097206, and ZQU with the ABCB1 transporter.

Decomposing the MM-GBSA binding affinities was conducted to point out the nature of interactions between the inspected compounds and the ABCB1 transporter. *E*_vdW_ was the main contributor to the UMHSN00009999-, UMHSN00097206-, and ZQU-ABCB1 binding affinities with average values of –71.6, –67.9, and –66.7 kcal/mol, respectively. *E*_ele_ forces were also favorable for UMHSN00009999, UMHSN00097206, and ZQU complexed with ABCB1 transporter with average values of –51.1, –35.4, and –15.6 kcal/mol, respectively (Fig 4).

**Fig 5:**
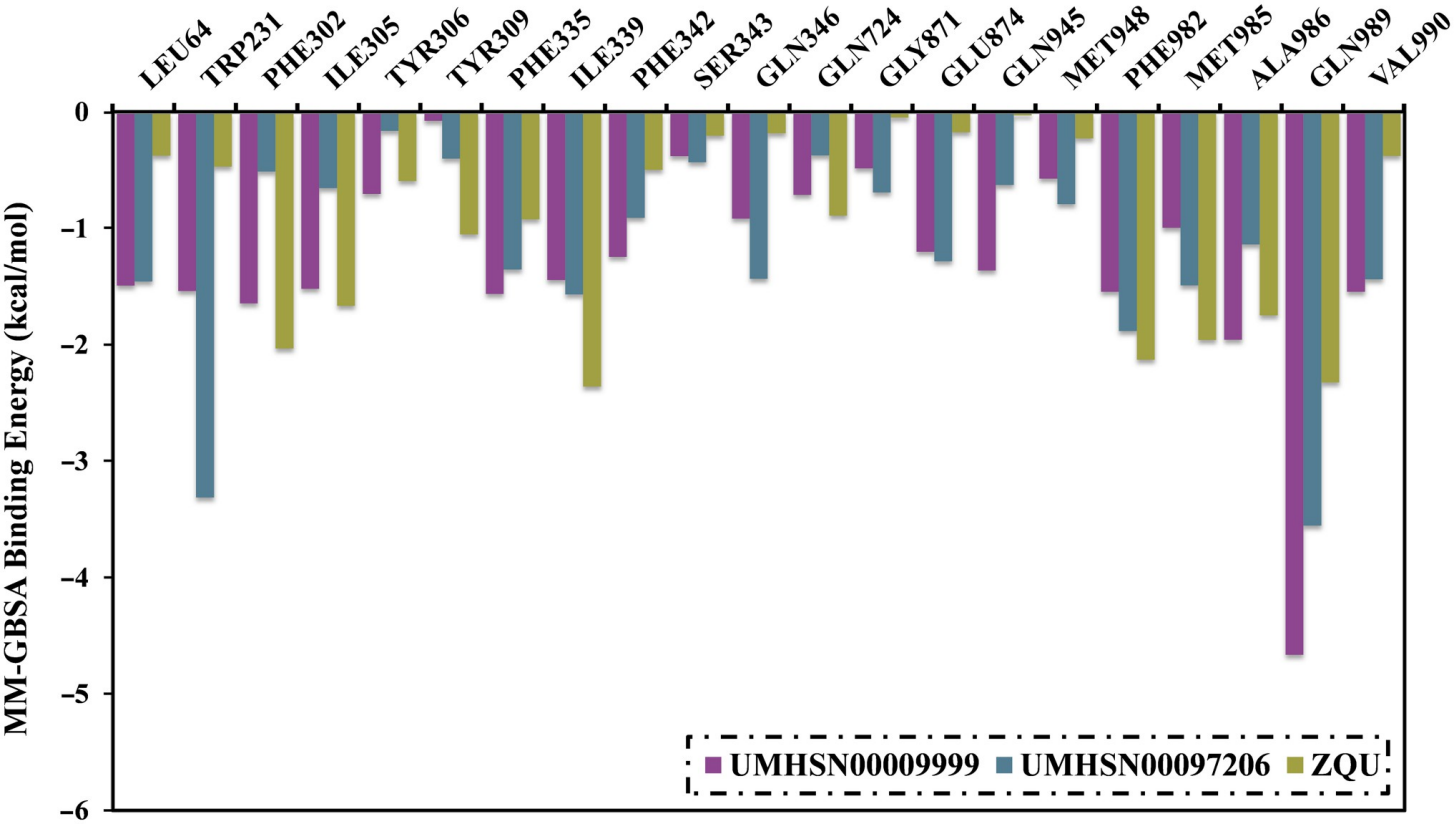
Energy participation of the key residues to the total binding energy (in kcal/mol) of UMHSN00009999, UMHSN00097206, and ZQU in complex with the ABCB1 transporter throughout 100 ns MD simulations.

To identify specific residues that bind the inhibitor to the transporter over the course of the MD simulations, per-residue decomposition analysis was examined (Fig 5). It is noteworthy that only residues with Δ*G*_binding_ lower than –0.5 kcal/mol were under consideration in per-residue energy decomposition analysis. As depicted in Fig 5, VAL990, ILE339, and GLN989 residues participated in interactions < –0.5 kcal/mol with UMHSN00009999, UMHSN00097206, and ZQU. GLN989 residue strongly affected the potential binding affinity with values of –4.7, –2.7, and –2.3 kcal/mol for UMHSN00009999-, UMHSN00097206-, and ZQU-ABCB1 complexes, respectively.

### Post-dynamics analyses

Compounds UMHSN00009999 and UMHSN00097206 complexed with ABCB1 transporter were further examined for stability using structural and energetical analyses throughout 100 ns MD simulations and compared with ZQU inhibitor.

#### Binding energy per-frame

**Fig 6:**
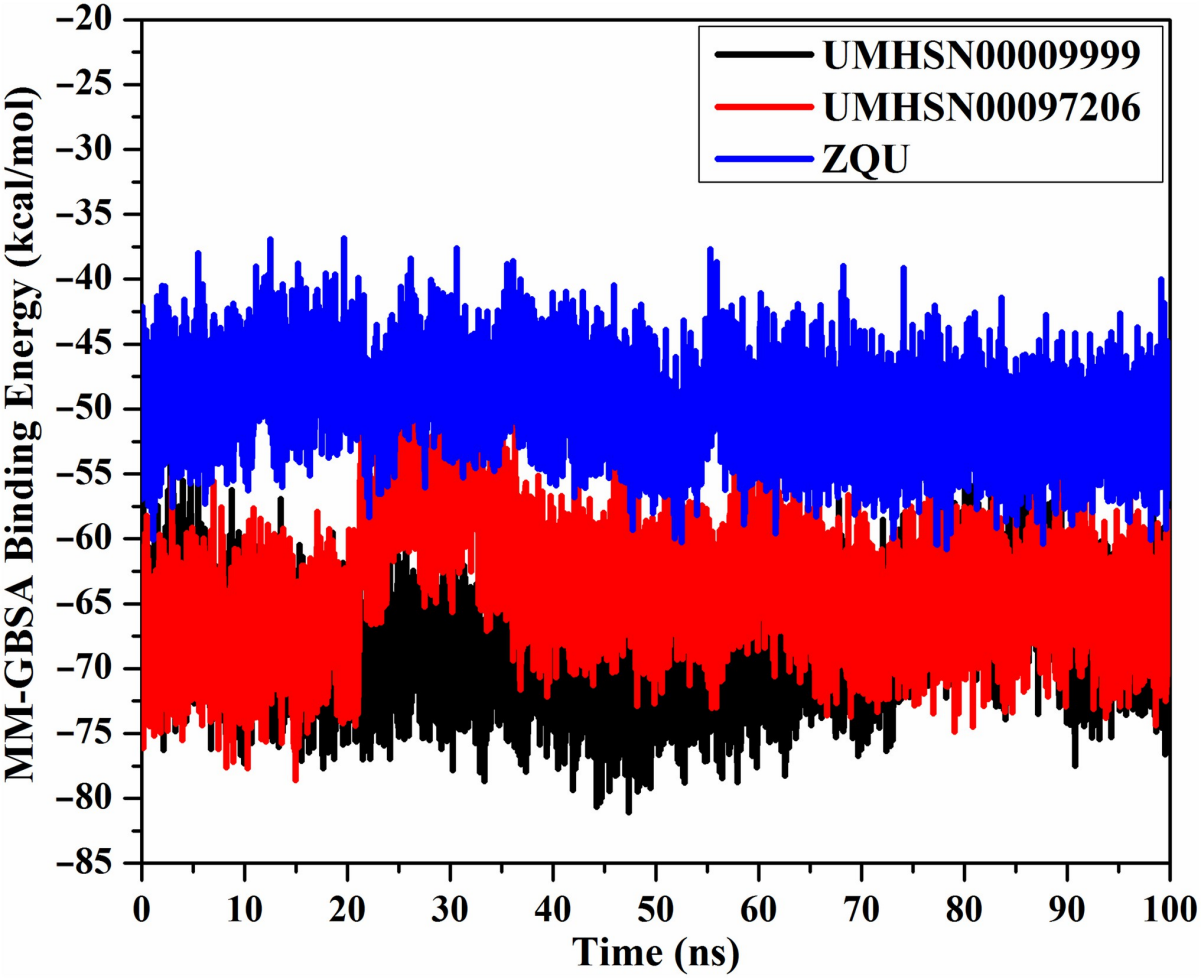
Binding energies per-frame for UMHSN00009999 (in black), UMHSN00097206 (in red), and ZQU (in blue) in complex with the ABCB1 transporter throughout 100 ns MD simulations.

Using the correlation between binding energy and time, the energetical stability of ABCB1 complexed with UMHSN00009999, UMHSN00097206, and ZQU was inspected over the 100 ns MD simulations (Fig 6). As depicted in Fig 6, overall stability was observed for UMHSN00009999, UMHSN00097206, and ZQU complexed with ABCB1 with average Δ*G*_binding_ values of –68.3, –64.1, and –49.8 kcal/mol, respectively. On the basis of this investigation, the studied complexes preserved stabilization thoughout the 100 ns MD simulations.

#### Hydrogen bond analysis

Identified compounds and ZQU hydrogen bonding was assessed in Table 2. Table 2 lists distances, occupancy, and hydrogen bond angles for critical ABCB1 residues. Compound UMHSN0009999 formed two stabled hydrogen bonds with GLN989 residue with occupancies and bond lengths of 93.5% and 2.8 Å and 86.2% and 2.8 Å, respectively (Table 2). UMHSN00097206 displayed one hydrogen bond with GLN989 residue (2.8 Å and 91.9% occupancy). This remarkably high hydrogen bond occupancy revealed the importance of GLN989 residue inside the ABCB1 binding pocket, which was in conformity with the per residue decomposition data. On the other hand, ZQU demonstrated one hydrogen bond with GLN989 residue (2.9 Å and 59.8% occupancy). It is worth noting that this bond was absent in the docked pose of ZQU within the ABCB1 binding pocket. Compared to ZQU-ABCB1 complex, intermolecular hydrogen bonding demonstrated that the UMHSN00009999- and UMHSN00097206- ABCB1 complexes are more stable.

**Table 2:** Hydrogen bond distances, occupancies, and angles between ABCB1 transporter amino acids, identified compounds and ZQU.

SuperNatural II Code	Acceptor	Donor	Distance (Å)^a^	Angle (degree)^a^	Occupied (%)^b^
**ZQU**	GLN989@O	ZQU@O-H	2.9	161.4	59.8
**UMHSN00009999**	GLN989@O	UMHSN00009999@N-H	2.8	150.3	93.5
	GLN989@O	UMHSN00009999@N-H	2.9	158.6	86.2
**UMHSN00097206**	GLN989@O	UMHSN00097206@N-H	2.8	151.1	91.9

^a^The hydrogen bonds are inspected via the acceptor-donor atom distance of < 3.5 Å and acceptor hydrogen donor angle of > 120°.

^b^Only hydrogen bonds with occupancy > 50% were elucidated.

#### Center-of-mass distance (CoM)

**Fig 7:**
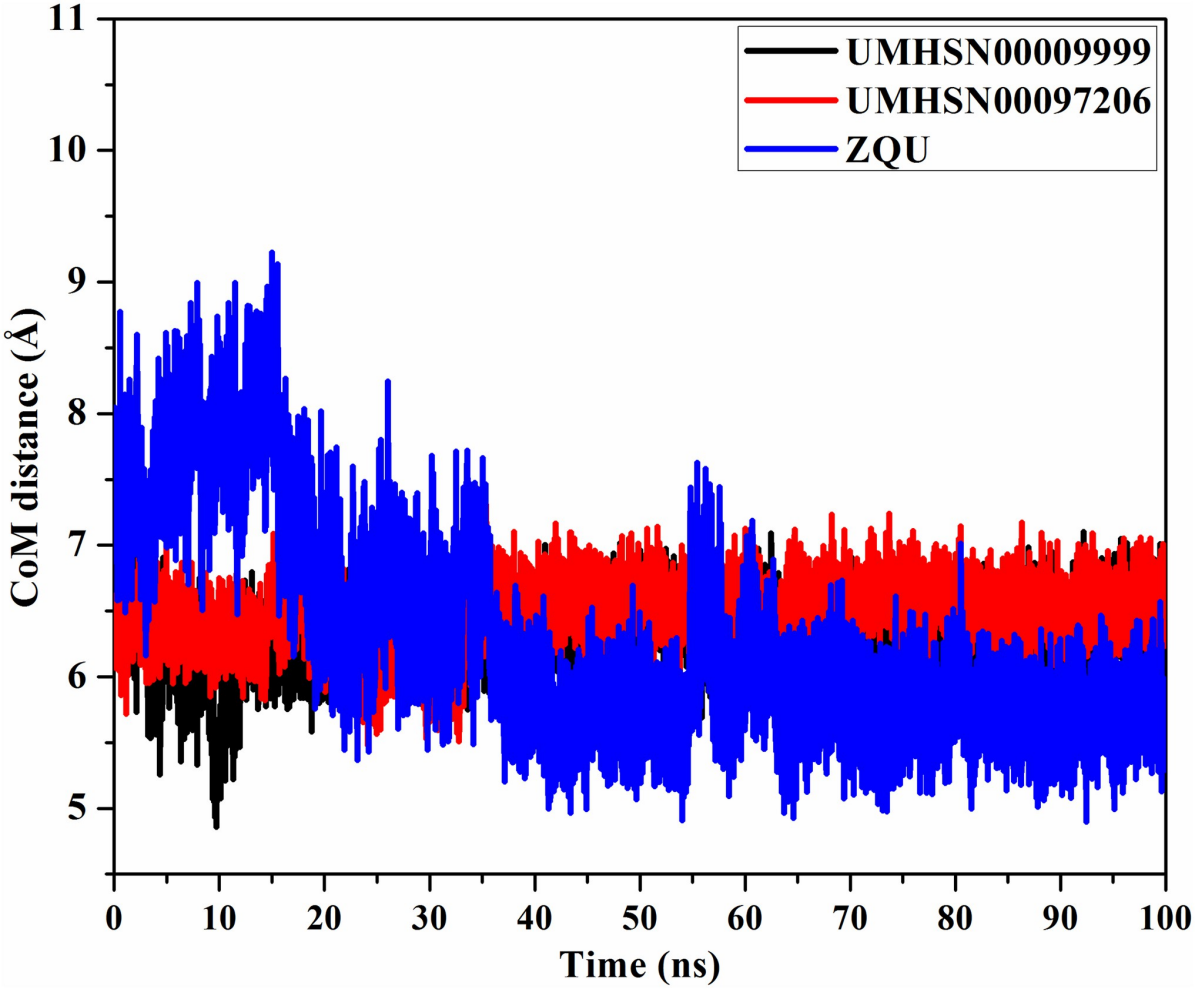
CoM distances (in Å) between UMHSN00009999 (in black), UMHSN00097206 (in red), and ZQU (in blue) and GLN989 residue of the ABCB1 transporter over 100 ns MD simulations.

The CoM distance between the inspected natural compounds and GLN989 residue was measured to better understand the persistence of the inhibitor-ABCB1 complexes over 100 ns MD simulations (Fig 7). CoM graph shows average values of 6.3, 6.5, and 6.3 Å for UMHSN00009999, UMHSN00097206, and ZQU, respectively. Distances were more consistent for UMHSN00009999 and UMHSN00097206 than for the ZQU. These results again indicate that ABCB1 binds more strongly to the identified compounds than ZQU.

#### Root-mean-square deviation (RMSD)

**Fig 8:**
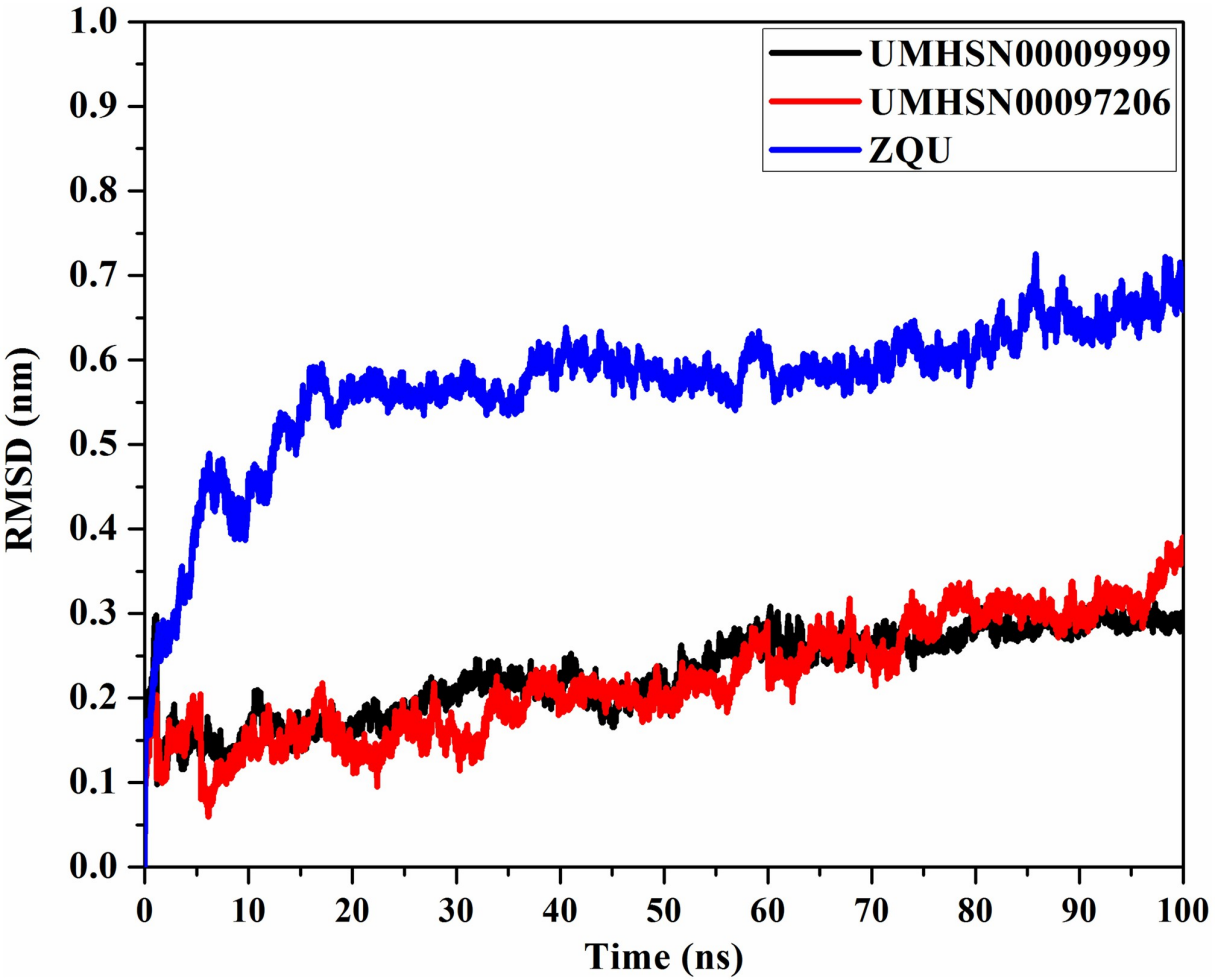
RMSD of UMHSN00009999 (in black), UMHSN00097206 (in red), and ZQU (in blue) with the ABCB1 transporter over 100 ns MD simulations.

To investigate the conformational variations, the RMSD of the entire complex backbone atoms was estimated (Fig 8). Estimated RMSD values were 0.2, 0.2, and 0.6 nm for UMHSN00009999-, UMHSN00097206-, and ZQU-ABCB1 complexes, respectively. According to RMSD analysis, the systems stabilized at 10 ns and remained stable throughout the termination of the simulation. Inferred from these findings is the tight compound binding with the ABCB1 transporter occurs without disturbance to the overall structure.

### Pharmacokinetic characteristics

Clinical trial failures of candidate drugs can be prevented by identifying drug similarities and pharmacokinetic properties during drug design [58]. The predicted pharmacokinetic properties for UMHSN00009999, UMHSN00097206, and ZQU are listed in Table 3. For evaluating the effectiveness of metabolites for oral administration, absorption is predicted based on HIA and Caco-2 permeability. If Caco-2 permeability is > 0.90, the molecule is highly absorbed. Additionally, a compound with an HIA of less than 30% is considered to be poorly absorbed. The current work demonstrated that the Caco-2 permeability for UMHSN00009999, UMHSN11197206, and ZQU is highly absorbed, with values of 0.96, 1.1, and 1.3 cm/s, respectively (Table 3). According to the predicted HIA, UMHSN00009999, UMHSN11197206 and ZQU demonstrated plausible absorbance with values of 89.4, 98.5, and 92.4%, respectively (Table 3).

**Table 3:** Predicted pharmacokinetic properties of the candidate inhibitors UMHSN00009999 and UMHSN00097206 compared to ZQU utilizing pkCSM server.

Inhibitor Code	Absorption (A)		Distribution (D)	Metabolism (M)	Excretion (E)	Toxicity (T)
	Caco2 Permeability (cm/s)	Human Intestinal Absorption (HIA)	VDss (human)	CYP3A4 Inhibitor	Total Clearance	AMES toxicity
**ZQU**	1.3	92.4	0.82	Yes	0.78	Yes
**UMHSN00009999**	0.96	89.4	0.59	Yes	0.47	No
**UMHSN000097206**	1.1	98.5	0.08	Yes	0.61	No

Similarly, VDss was utilized for determining the internal distribution within the human body. Compound UMHSN00009999 and ZQU showed faster diffusion than UMHSN00097206, with VDss values of 0.59, 0.82, and 0.08 L/kg, respectively. A VDss score of less than –0.15 L/kg is viewed negatively, while VDss values greater than 0.45 L/kg are interpreted as good diffusion. The cytochrome model CYP3A4 was used to assess the predicted because this enzyme is crucial for the metabolism of many drugs. From Table 3, UMHSN00009999, UMHSN00097206, and ZQU demonstrated their potentialities as CYP3A4 inhibitors. Moreover, UMHSN00009999, UMHSN00097206, and ZQU showed total clearance of 0.47, 0.61, and 0.78 log ml/min/kg, respectively. Besides, UMHSN00009999 and UMHSN00097206 did not show AMES toxicity rates. In contrast, ZQU was toxic according to the AMES toxicity (Table 3). The ADMET investigations indicated that the identified compounds have a high probability of being an effective therapeutic candidate for reversing MDR process.

### MD simulation in the lipid bilayer

**Fig 9:**
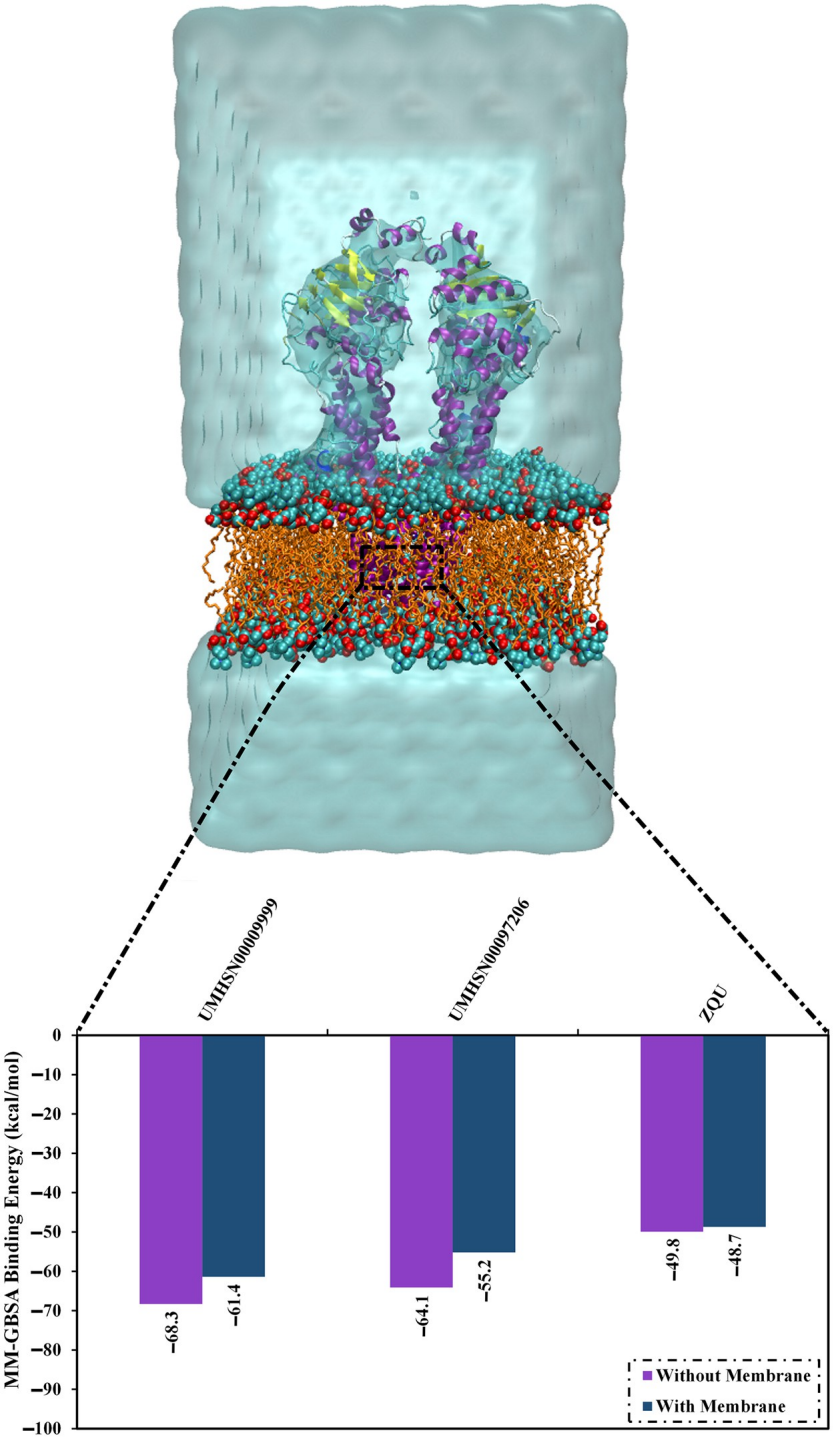
Binding energies for UMHSN00009999, UMHSN00097206, and ZQU within the ABCB1 binding pocket with and without the POPC membrane throughout the 100 ns MD simulations.

To mimic physiological conditions, compound stability was further investigated with the transporter immersed in a POPC bilayer. The Δ*G*_binding_ values were –61.4, –55.2, and –48.7 kcal/mol for UMHSN00009999, UMHSN00097206, and ZQU, respectively (Fig 9). Compared to no POPC-based results, the POPC bilayer existence demonstrated no significant impact on the binding energies of the identified natural compounds inside the ABCB1 binding pocket (Fig 9).

## Conclusions

Inhibiting the ABCB1 transporter is one of the most effective mechanisms for reversing multidrug resistance (MDR). Herein, *in-silico* techniques were used for screening the SuperNatural II database to point out potential compounds towards the ABCB1 transporter. On the basis of the docking scores, the potent natural compounds with docking scores ≤ –11.0 kcal/mol were subjected to MD simulations, pursued by MM-GBSA binding energy computations. In accordance with the MM-GBSA results, UMHSN00009999 and UMHSN00097206 showed superior binding energies with average Δ*G*_binding_ values of –68.3 and –64.1 kcal/mol over 100 ns MD simulations, respectively, compared to the ZQU (calc. –49.8 kcal/mol). The structural and energetical analyses confirmed inhibitor stability over the 100 ns MD simulation. According to pharmacokinetic properties, UMHSN000099999 and UMHSN00097206 demonstrated good oral bioavailability. In addition, MD simulations executed in a POPC environment revealed no significant change in inhibitor binding affinities. In summary, these findings revealed promising, novel, and effective ABCB1 inhibitors that can reverse MDR and can be deemed as a starting point for lead optimization.

## Supporting information

S1 Fig The anticipated binding modes of the top 39 scoring compounds within the active site of the ABCB1 transporter. (DOCX)

S1 Table Calculated standard and moderate docking scores (in kcal/mol) for the top 3751 SuperNatural II compounds and ZQU against the ABCB1 transporter protein. (DOCX)

S2 Table Estimated standard, moderate, and expensive docking scores (in kcal/mol) for the top 376 compounds and ZQU within the ABCB1 binding pocket. (DOCX)

S3 Table Evaluated standard, moderate, and expensive docking scores and MM-GBSA over 5 ns MD simulations (in kcal/mol) for the top 39 compounds and ZQU within the ABCB1 binding pocket. (DOCX)

S1 Graphical abstract (TIF)
