# Supplementary material for: SuperNatural inhibitors to reverse multidrug resistance emerged by ABCB1 transporter: Database mining, lipid-mediated molecular dynamics, and pharmacokinetics study
Source: PLoS One. 2023 Jul 26;18(7):e0288919. doi: 10.1371/journal.pone.0288919 (PMC10370898; doi:10.1371/journal.pone.0288919)
Supplement: S1 Fig — (DOCX) [file pone.0288919.s001.docx]

**S1 Fig.** The anticipated binding modes of the top 39 scoring compounds within the active site of the ABCB1 transporter.

**
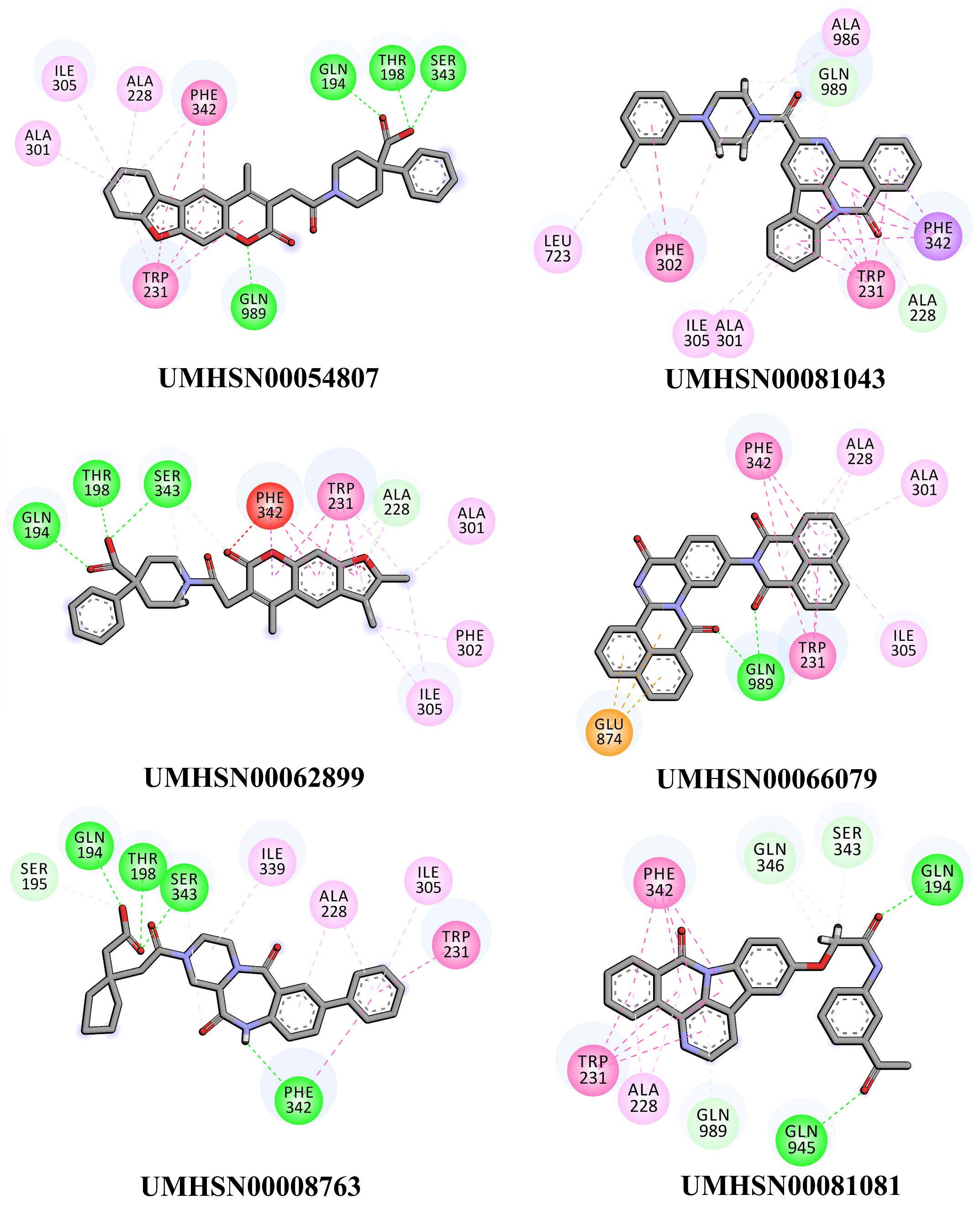
**


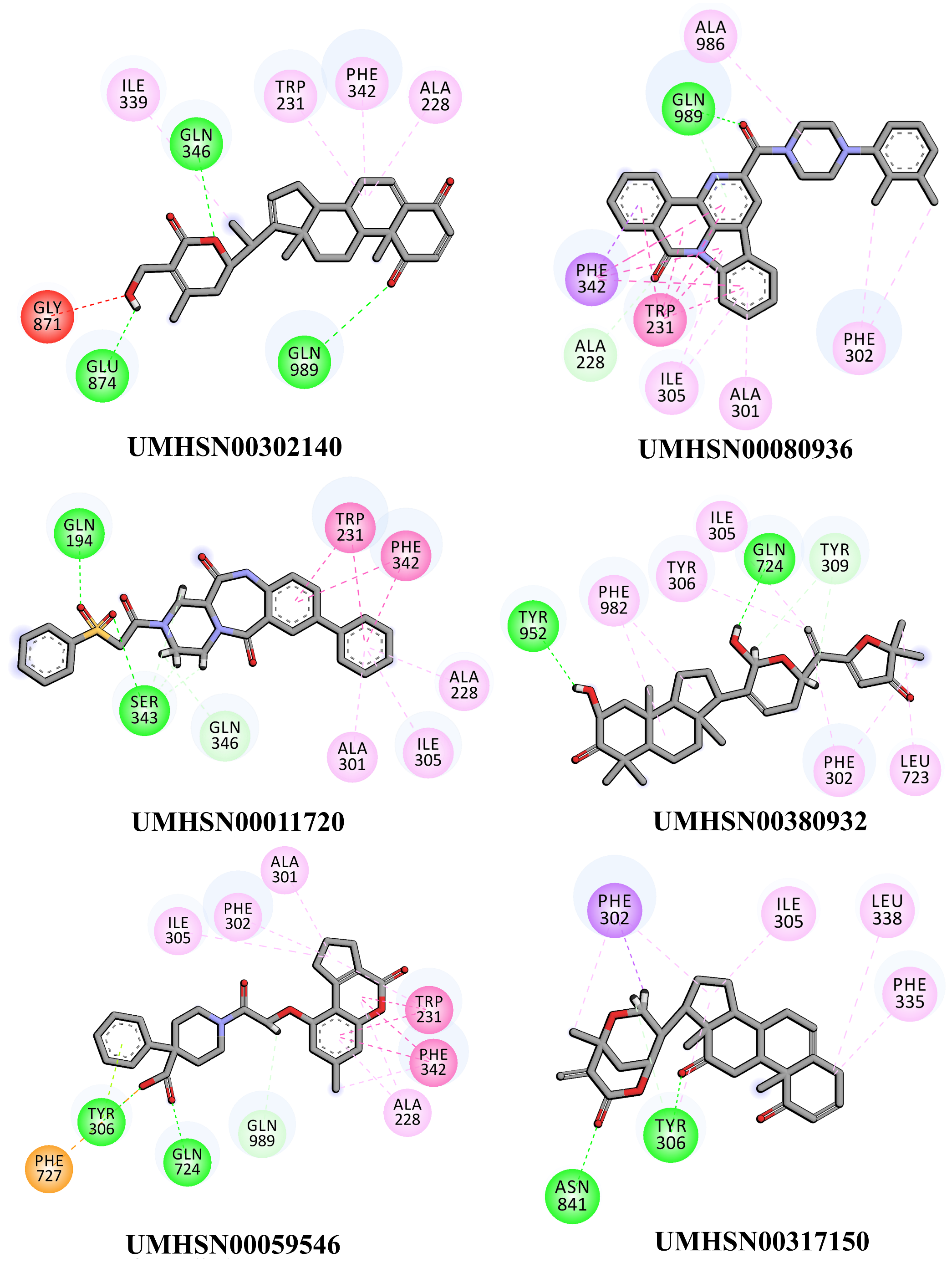


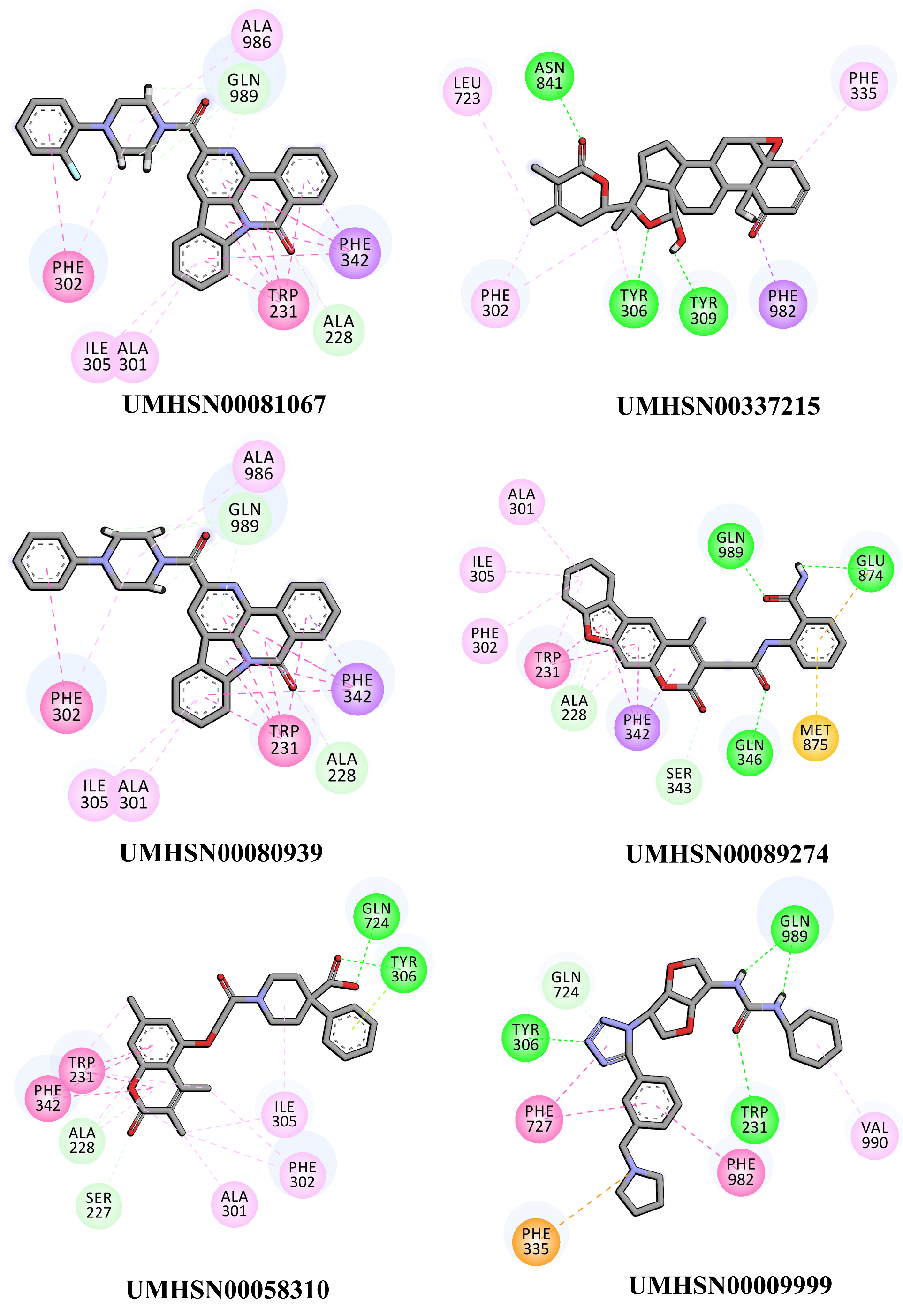


*
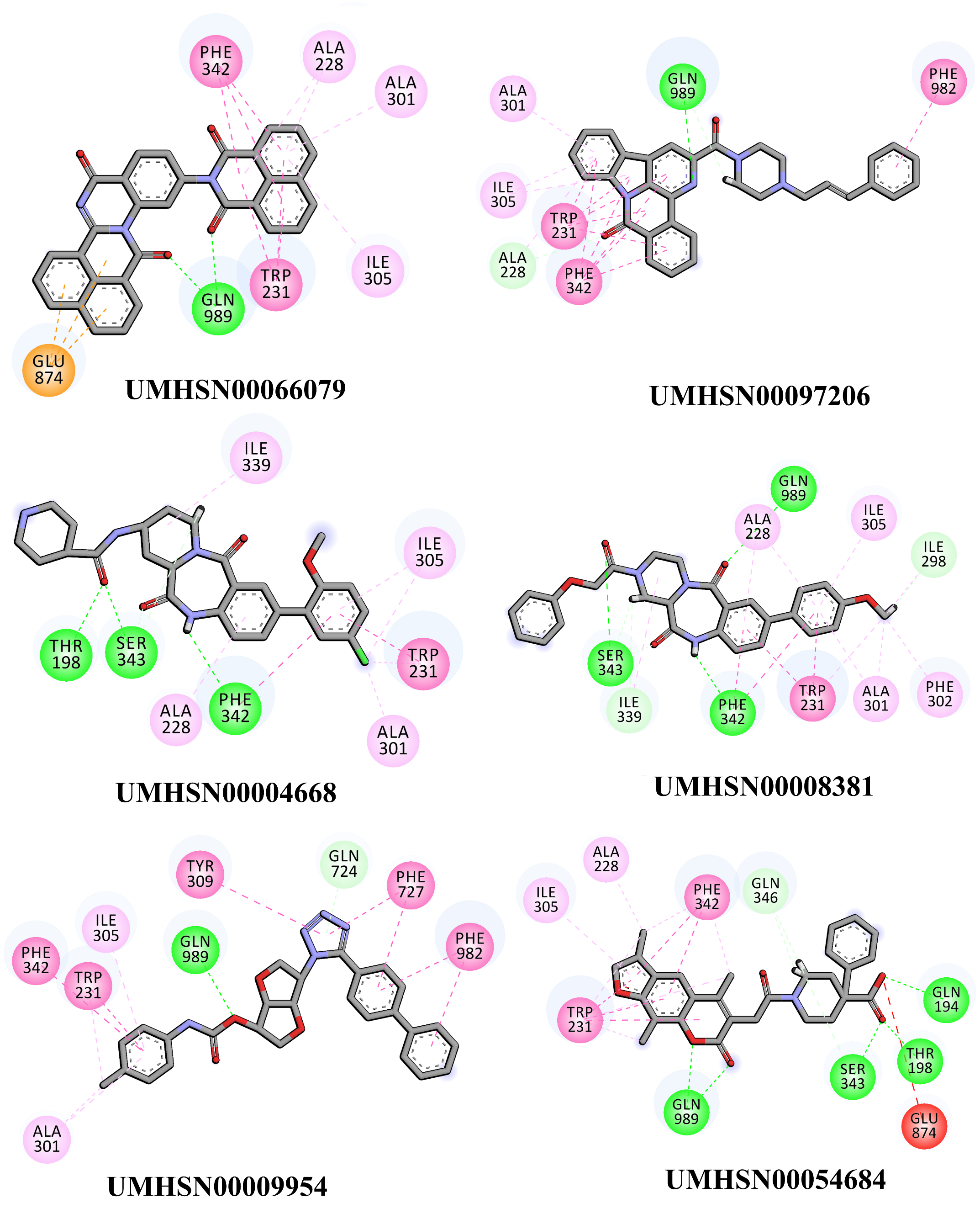
*

*
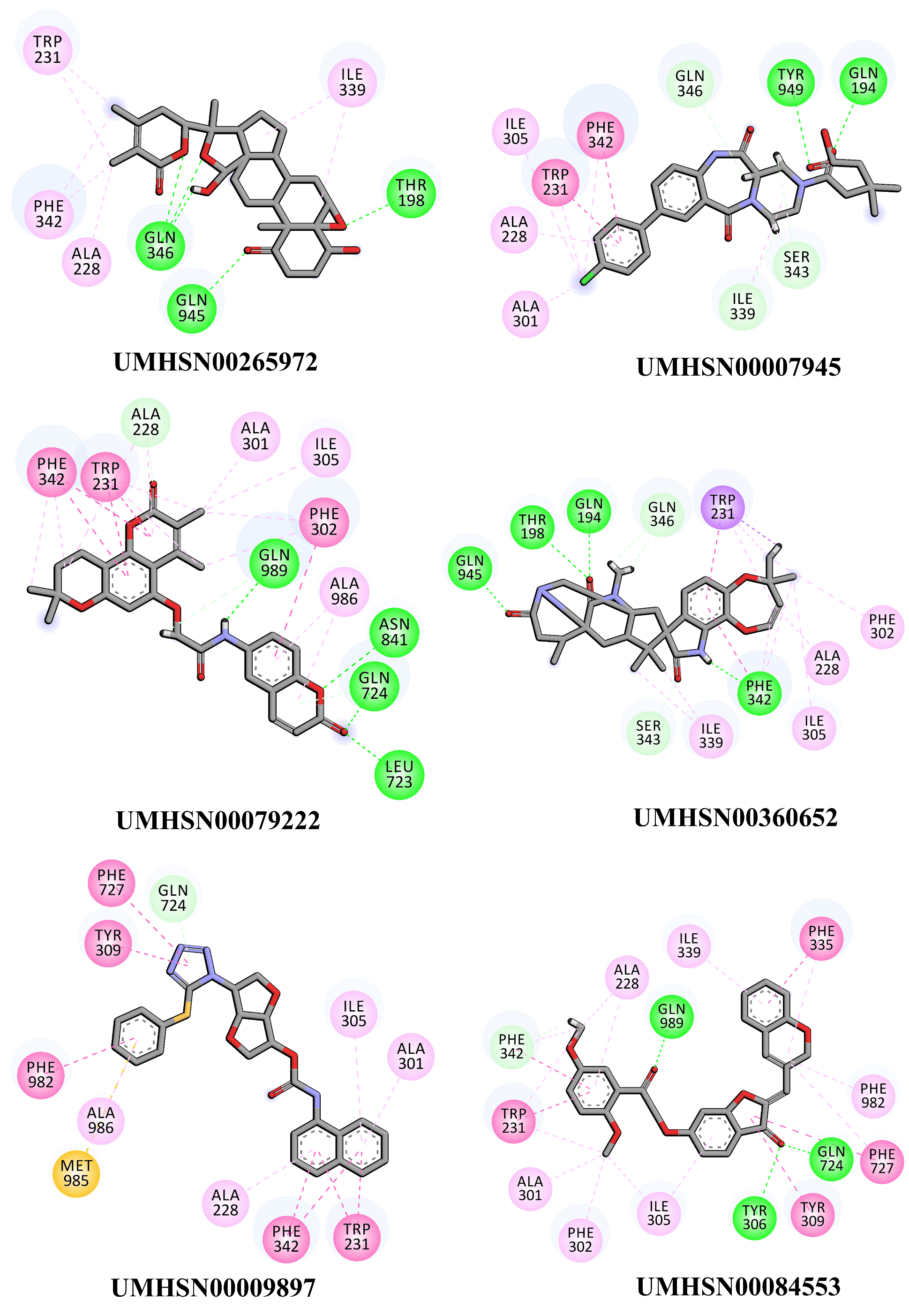
*

*
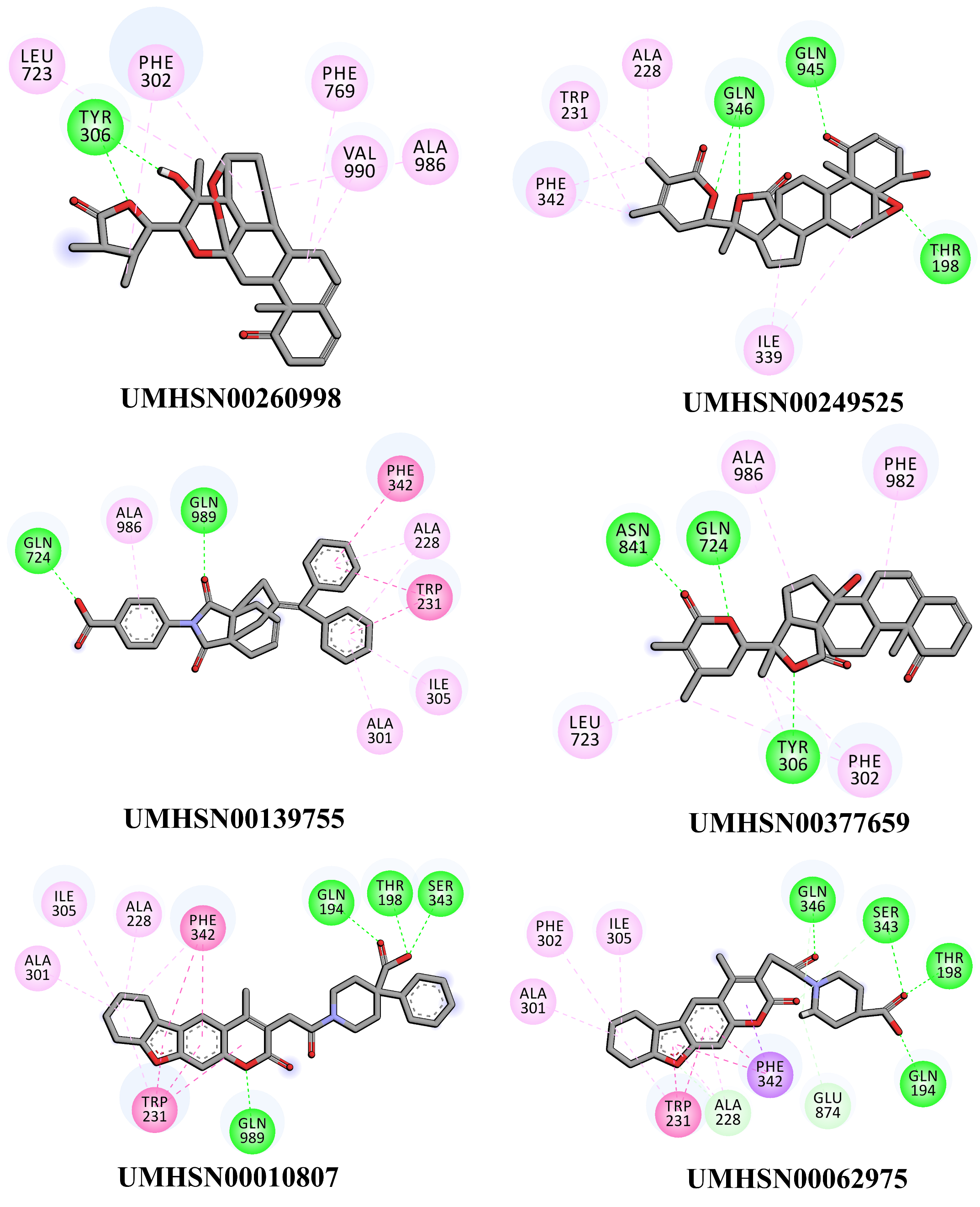
*

*
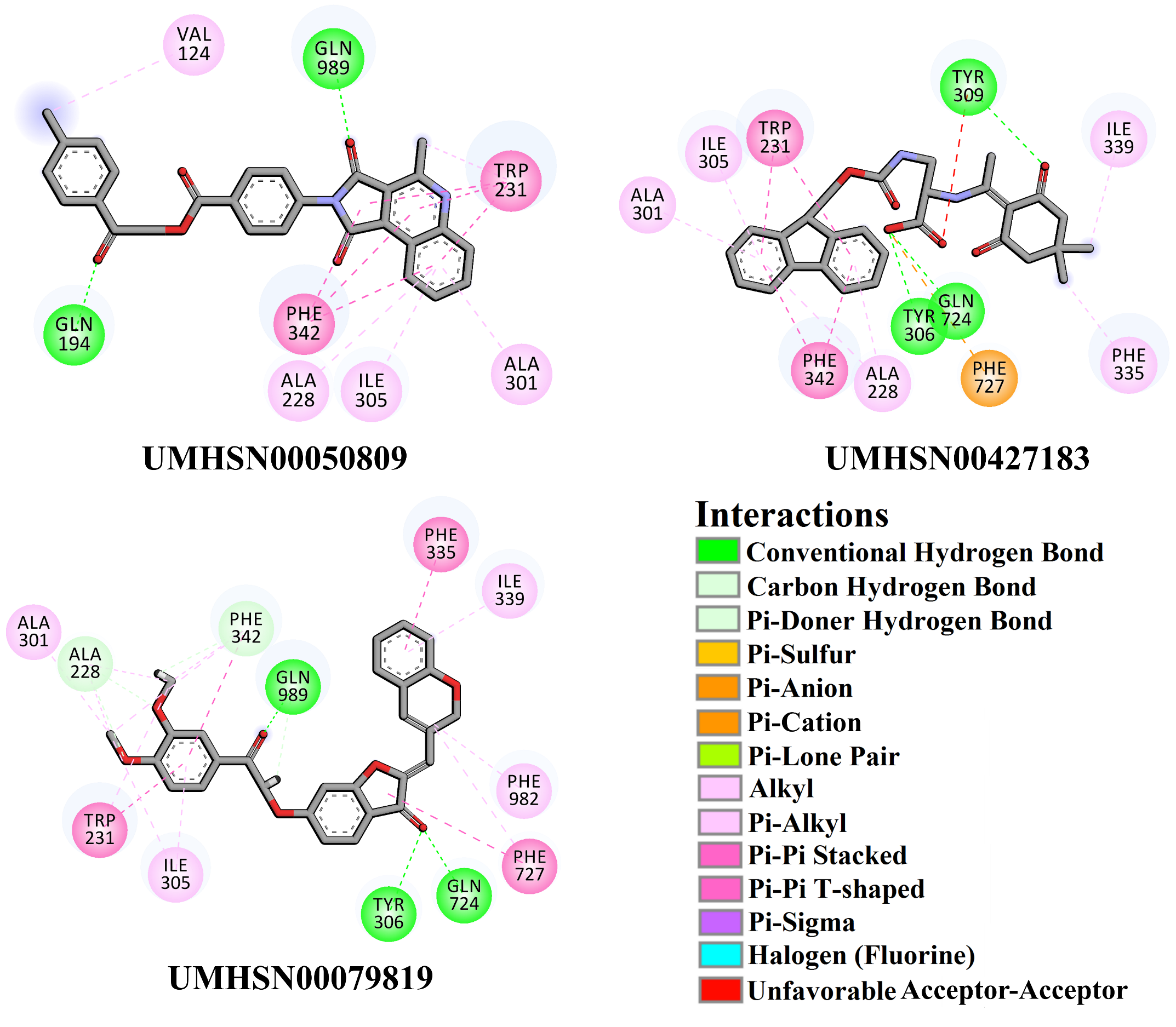
*
