# Supplementary material for: SuperNatural inhibitors to reverse multidrug resistance emerged by ABCB1 transporter: Database mining, lipid-mediated molecular dynamics, and pharmacokinetics study
Source: PLoS One. 2023 Jul 26;18(7):e0288919. doi: 10.1371/journal.pone.0288919 (PMC10370898; doi:10.1371/journal.pone.0288919)
Supplement: S1 Table — (DOCX) [file pone.0288919.s002.docx]

### S1 Table. Calculated standard and moderate docking scores (in kcal/mol) for the top 3751 SuperNatural II compounds and ZQU against the ABCB1 transporter protein. ^a^

| No. | SuperNatural II Code | Docking Score (kcal/mol) | | No. | SuperNatural II Code | Docking Score (kcal/mol) | |
| --- | --- | --- | --- | --- | --- | --- | --- |
|  |  | Std.^b^ | Mod.^c^ |  |  | Std.^b^ | Mod.^c^ |
|  | **ZQU** | **–8.4** | **–8.2** | 50 | UMHSN00080908 | –9.7 | –11.3 |
| 1 | UMHSN00009999 | –12.5 | –13.1 | 51 | UMHSN00342900 | –11.2 | –11.3 |
| 2 | UMHSN00054807 | –12.5 | –13.1 | 52 | UMHSN00007078 | –11.0 | –11.2 |
| 3 | UMHSN00011720 | –12.7 | –12.8 | 53 | UMHSN00010794 | –11.2 | –11.2 |
| 4 | UMHSN00081043 | –12.5 | –12.6 | 54 | UMHSN00009733 | –10.5 | –11.2 |
| 5 | UMHSN00066079 | –12.4 | –12.4 | 55 | UMHSN00345449 | –11.2 | –11.2 |
| 6 | UMHSN00062899 | –12.1 | –12.4 | 56 | UMHSN00008336 | –11.2 | –11.2 |
| 7 | UMHSN00302140 | –12.1 | –12.1 | 57 | UMHSN00060131 | –10.7 | –11.2 |
| 8 | UMHSN00081735 | –11.8 | –12.0 | 58 | UMHSN00080951 | –9.8 | –11.2 |
| 9 | UMHSN00080936 | –11.9 | –11.9 | 59 | UMHSN00008510 | –11.2 | –11.2 |
| 10 | UMHSN00008763 | –10.4 | –11.9 | 60 | UMHSN00009039 | –10.3 | –11.2 |
| 11 | UMHSN00080939 | –11.8 | –11.8 | 61 | UMHSN00089524 | –9.5 | –11.2 |
| 12 | UMHSN00081067 | –11.8 | –11.8 | 62 | UMHSN00265761 | –11.1 | –11.2 |
| 13 | UMHSN00009897 | –11.2 | –11.8 | 63 | UMHSN00080511 | –11.1 | –11.2 |
| 14 | UMHSN00337215 | –11.8 | –11.8 | 64 | UMHSN00084545 | –10.8 | –11.1 |
| 15 | UMHSN00058310 | –10.5 | –11.8 | 65 | UMHSN00081514 | –10.7 | –11.1 |
| 16 | UMHSN00380932 | –11.6 | –11.8 | 66 | UMHSN00321430 | –11.0 | –11.1 |
| 17 | UMHSN00089274 | –11.5 | –11.8 | 67 | UMHSN00061044 | –10.5 | –11.1 |
| 18 | UMHSN00317150 | –11.8 | –11.8 | 68 | UMHSN00009957 | –10.4 | –11.1 |
| 19 | UMHSN00081079 | –11.7 | –11.7 | 69 | UMHSN00081681 | –10.8 | –11.1 |
| 20 | UMHSN00062975 | –11.5 | –11.6 | 70 | UMHSN00097225 | –11.1 | –11.1 |
| 21 | UMHSN00004668 | –11.0 | –11.6 | 71 | UMHSN00234079 | –9.8 | –11.1 |
| 22 | UMHSN00050809 | –9.9 | –11.6 | 72 | UMHSN00080836 | –10.9 | –11.1 |
| 23 | UMHSN00059546 | –10.8 | –11.6 | 73 | UMHSN00081691 | –10.4 | –11.1 |
| 24 | UMHSN00097206 | –12.5 | –11.5 | 74 | UMHSN00274123 | –11.0 | –11.1 |
| 25 | UMHSN00009954 | –11.4 | –11.5 | 75 | UMHSN00048096 | –10.9 | –11.1 |
| 26 | UMHSN00360652 | –11.5 | –11.5 | 76 | UMHSN00059415 | –10.8 | –11.1 |
| 27 | UMHSN00008381 | –10.0 | –11.5 | 77 | UMHSN00335397 | –10.9 | –11.1 |
| 28 | UMHSN00007945 | –10.2 | –11.5 | 78 | UMHSN00011710 | –11.0 | –11.0 |
| 29 | UMHSN00265972 | –11.5 | –11.5 | 79 | UMHSN00063515 | –10.4 | –11.0 |
| 30 | UMHSN00260998 | –10.9 | –11.5 | 80 | UMHSN00168544 | –11.0 | –11.0 |
| 31 | UMHSN00054684 | –11.4 | –11.5 | 81 | UMHSN00286721 | –10.0 | –11.0 |
| 32 | UMHSN00010807 | –11.3 | –11.4 | 82 | UMHSN00009721 | –11.0 | –11.0 |
| 33 | UMHSN00249525 | –11.4 | –11.4 | 83 | UMHSN00010270 | –9.4 | –11.0 |
| 34 | UMHSN00079222 | –11.2 | –11.4 | 84 | UMHSN00014785 | –11.0 | –11.0 |
| 35 | UMHSN00139755 | –9.7 | –11.4 | 85 | UMHSN00004210 | –11.0 | –11.0 |
| 36 | UMHSN00377659 | –11.4 | –11.4 | 86 | UMHSN00291292 | –11.0 | –11.0 |
| 37 | UMHSN00378848 | –11.4 | –11.4 | 87 | UMHSN00014775 | –10.9 | –11.0 |
| 38 | UMHSN00427183 | –10.9 | –11.4 | 88 | UMHSN00079819 | –10.3 | –11.0 |
| 39 | UMHSN00084553 | –11.1 | –11.4 | 89 | UMHSN00235196 | –10.5 | –11.0 |
| 40 | UMHSN00081530 | –10.7 | –11.3 | 90 | UMHSN00013456 | –10.5 | –11.0 |
| 41 | UMHSN00249560 | –11.3 | –11.3 | 91 | UMHSN00014834 | –11.0 | –11.0 |
| 42 | UMHSN00005185 | –11.3 | –11.3 | 92 | UMHSN00133181 | –10.5 | –11.0 |
| 43 | UMHSN00081749 | –11.3 | –11.3 | 93 | UMHSN00053680 | –9.5 | –11.0 |
| 44 | UMHSN00089681 | –10.3 | –11.3 | 94 | UMHSN00054437 | –10.1 | –11.0 |
| 45 | UMHSN00008385 | –11.1 | –11.3 | 95 | UMHSN00081583 | –10.7 | –11.0 |
| 46 | UMHSN00087888 | –10.5 | –11.3 | 96 | UMHSN00006367 | –9.4 | –11.0 |
| 47 | UMHSN00084550 | –11.2 | –11.3 | 97 | UMHSN00008771 | –10.5 | –11.0 |
| 48 | UMHSN00009953 | –11.2 | –11.3 | 98 | UMHSN00008774 | –11.0 | –11.0 |
| 49 | UMHSN00084547 | –10.9 | –11.3 | 99 | UMHSN00089689 | –10.8 | –11.0 |

| No. | SuperNatural II Code | Docking Score (kcal/mol) | | No. | SuperNatural II Code | Docking Score (kcal/mol) | |
| --- | --- | --- | --- | --- | --- | --- | --- |
|  |  | Std.^b^ | Mod.^c^ |  |  | Std.^b^ | Mod.^c^ |
| 100 | UMHSN00011707 | –10.9 | –11.0 | 151 | UMHSN00004639 | –10.7 | –10.8 |
| 101 | UMHSN00079235 | –10.9 | –11.0 | 152 | UMHSN00005544 | –9.6 | –10.8 |
| 102 | UMHSN00082123 | –11.0 | –11.0 | 153 | UMHSN00008384 | –10.6 | –10.8 |
| 103 | UMHSN00101579 | –11.0 | –11.0 | 154 | UMHSN00016061 | –11.0 | –10.8 |
| 104 | UMHSN00119051 | –10.6 | –11.0 | 155 | UMHSN00049070 | –10.7 | –10.8 |
| 105 | UMHSN00295529 | –10.9 | –11.0 | 156 | UMHSN00062547 | –10.4 | –10.8 |
| 106 | UMHSN00009729 | –11.0 | –11.0 | 157 | UMHSN00081754 | –10.7 | –10.8 |
| 107 | UMHSN00014817 | –10.8 | –11.0 | 158 | UMHSN00082803 | –10.8 | –10.8 |
| 108 | UMHSN00008340 | –10.9 | –10.9 | 159 | UMHSN00004200 | –10.8 | –10.8 |
| 109 | UMHSN00060190 | –10.6 | –10.9 | 160 | UMHSN00008338 | –10.8 | –10.8 |
| 110 | UMHSN00148374 | –10.3 | –10.9 | 161 | UMHSN00011773 | –10.6 | –10.8 |
| 111 | UMHSN00151638 | –10.3 | –10.9 | 162 | UMHSN00014784 | –10.7 | –10.8 |
| 112 | UMHSN00055285 | –10.8 | –10.9 | 163 | UMHSN00014909 | –10.8 | –10.8 |
| 113 | UMHSN00127113 | –9.6 | –10.9 | 164 | UMHSN00018080 | –10.0 | –10.8 |
| 114 | UMHSN00004196 | –10.9 | –10.9 | 165 | UMHSN00108809 | –10.5 | –10.8 |
| 115 | UMHSN00009839 | –10.9 | –10.9 | 166 | UMHSN00135413 | –10.7 | –10.8 |
| 116 | UMHSN00061743 | –10.1 | –10.9 | 167 | UMHSN00150154 | –10.7 | –10.8 |
| 117 | UMHSN00081767 | –10.9 | –10.9 | 168 | UMHSN00151328 | –10.3 | –10.8 |
| 118 | UMHSN00150166 | –10.8 | –10.9 | 169 | UMHSN00278326 | –10.6 | –10.8 |
| 119 | UMHSN00008793 | –10.7 | –10.9 | 170 | UMHSN00323517 | –10.8 | –10.8 |
| 120 | UMHSN00052580 | –9.5 | –10.9 | 171 | UMHSN00336301 | –9.7 | –10.8 |
| 121 | UMHSN00091326 | –10.9 | –10.9 | 172 | UMHSN00008406 | –10.7 | –10.8 |
| 122 | UMHSN00106227 | –10.8 | –10.9 | 173 | UMHSN00115760 | –10.7 | –10.8 |
| 123 | UMHSN00294698 | –10.8 | –10.9 | 174 | UMHSN00253790 | –10.7 | –10.8 |
| 124 | UMHSN00005497 | –10.7 | –10.9 | 175 | UMHSN00352446 | –10.8 | –10.8 |
| 125 | UMHSN00151994 | –10.1 | –10.9 | 176 | UMHSN00011744 | –10.3 | –10.8 |
| 126 | UMHSN00153504 | –10.8 | –10.9 | 177 | UMHSN00330061 | –10.8 | –10.8 |
| 127 | UMHSN00005221 | –9.9 | –10.9 | 178 | UMHSN00335968 | –10.7 | –10.8 |
| 128 | UMHSN00025816 | –10.3 | –10.9 | 179 | UMHSN00008429 | –10.7 | –10.8 |
| 129 | UMHSN00004201 | –10.7 | –10.9 | 180 | UMHSN00008443 | –10.8 | –10.8 |
| 130 | UMHSN00297776 | –10.9 | –10.9 | 181 | UMHSN00009723 | –10.3 | –10.8 |
| 131 | UMHSN00338707 | –10.8 | –10.9 | 182 | UMHSN00014774 | –10.8 | –10.8 |
| 132 | UMHSN00081578 | –10.8 | –10.9 | 183 | UMHSN00014899 | –10.7 | –10.8 |
| 133 | UMHSN00082600 | –9.5 | –10.9 | 184 | UMHSN00080671 | –10.6 | –10.8 |
| 134 | UMHSN00163698 | –9.6 | –10.8 | 185 | UMHSN00053252 | –9.6 | –10.8 |
| 135 | UMHSN00008441 | –10.8 | –10.8 | 186 | UMHSN00129049 | –10.4 | –10.8 |
| 136 | UMHSN00059308 | –10.8 | –10.8 | 187 | UMHSN00004204 | –10.8 | –10.7 |
| 137 | UMHSN00303503 | –10.8 | –10.8 | 188 | UMHSN00009829 | –9.6 | –10.7 |
| 138 | UMHSN00311964 | –9.7 | –10.8 | 189 | UMHSN00054815 | –10.4 | –10.7 |
| 139 | UMHSN00078921 | –10.4 | –10.8 | 190 | UMHSN00063317 | –10.7 | –10.7 |
| 140 | UMHSN00081712 | –9.7 | –10.8 | 191 | UMHSN00081740 | –10.7 | –10.7 |
| 141 | UMHSN00151329 | –10.0 | –10.8 | 192 | UMHSN00008071 | –9.6 | –10.7 |
| 142 | UMHSN00151996 | –9.7 | –10.8 | 193 | UMHSN00008792 | –10.5 | –10.7 |
| 143 | UMHSN00298864 | –10.8 | –10.8 | 194 | UMHSN00063180 | –9.5 | –10.7 |
| 144 | UMHSN00348298 | –10.8 | –10.8 | 195 | UMHSN00080018 | –10.6 | –10.7 |
| 145 | UMHSN00004205 | –10.8 | –10.8 | 196 | UMHSN00086813 | –10.6 | –10.7 |
| 146 | UMHSN00007954 | –10.7 | –10.8 | 197 | UMHSN00115713 | –10.7 | –10.7 |
| 147 | UMHSN00057710 | –10.8 | –10.8 | 198 | UMHSN00169956 | –10.7 | –10.7 |
| 148 | UMHSN00059472 | –10.2 | –10.8 | 199 | UMHSN00362600 | –10.2 | –10.7 |
| 149 | UMHSN00272837 | –10.8 | –10.8 | 200 | UMHSN00014810 | –10.7 | –10.7 |
| 150 | UMHSN00335595 | –9.8 | –10.8 | 201 | UMHSN00052398 | –10.2 | –10.7 |

| No. | SuperNatural II Code | Docking Score (kcal/mol) | | No. | SuperNatural II Code | Docking Score (kcal/mol) | |
| --- | --- | --- | --- | --- | --- | --- | --- |
|  |  | Std.^b^ | Mod.^c^ |  |  | Std.^b^ | Mod.^c^ |
| 202 | UMHSN00081783 | –10.5 | –10.7 | 253 | UMHSN00269911 | –10.6 | –10.6 |
| 203 | UMHSN00004667 | –10.7 | –10.7 | 254 | UMHSN00328645 | –10.6 | –10.6 |
| 204 | UMHSN00005492 | –10.7 | –10.7 | 255 | UMHSN00382692 | –10.5 | –10.6 |
| 205 | UMHSN00007947 | –11.7 | –10.7 | 256 | UMHSN00004207 | –10.6 | –10.6 |
| 206 | UMHSN00009841 | –10.5 | –10.7 | 257 | UMHSN00010013 | –10.2 | –10.6 |
| 207 | UMHSN00009979 | –10.4 | –10.7 | 258 | UMHSN00011706 | –10.6 | –10.6 |
| 208 | UMHSN00081763 | –10.7 | –10.7 | 259 | UMHSN00053443 | –10.3 | –10.6 |
| 209 | UMHSN00150295 | –9.7 | –10.7 | 260 | UMHSN00057073 | –10.6 | –10.6 |
| 210 | UMHSN00247576 | –10.7 | –10.7 | 261 | UMHSN00081550 | –10.4 | –10.6 |
| 211 | UMHSN00333049 | –10.7 | –10.7 | 262 | UMHSN00093977 | –10.1 | –10.6 |
| 212 | UMHSN00004206 | –10.7 | –10.7 | 263 | UMHSN00094477 | –10.2 | –10.6 |
| 213 | UMHSN00005427 | –10.6 | –10.7 | 264 | UMHSN00099052 | –10.5 | –10.6 |
| 214 | UMHSN00014892 | –10.7 | –10.7 | 265 | UMHSN00148369 | –10.4 | –10.6 |
| 215 | UMHSN00302589 | –9.9 | –10.7 | 266 | UMHSN00150153 | –10.0 | –10.6 |
| 216 | UMHSN00365861 | –10.7 | –10.7 | 267 | UMHSN00168542 | –10.6 | –10.6 |
| 217 | UMHSN00009187 | –10.7 | –10.7 | 268 | UMHSN00236730 | –10.6 | –10.6 |
| 218 | UMHSN00012291 | –9.9 | –10.7 | 269 | UMHSN00338048 | –10.6 | –10.6 |
| 219 | UMHSN00016053 | –10.6 | –10.7 | 270 | UMHSN00004203 | –10.6 | –10.6 |
| 220 | UMHSN00081816 | –10.7 | –10.7 | 271 | UMHSN00005184 | –10.6 | –10.6 |
| 221 | UMHSN00008597 | –10.5 | –10.7 | 272 | UMHSN00014022 | –10.6 | –10.6 |
| 222 | UMHSN00009725 | –10.8 | –10.7 | 273 | UMHSN00014824 | –10.6 | –10.6 |
| 223 | UMHSN00024180 | –9.4 | –10.7 | 274 | UMHSN00080976 | –10.4 | –10.6 |
| 224 | UMHSN00062729 | –9.5 | –10.7 | 275 | UMHSN00083478 | –9.9 | –10.6 |
| 225 | UMHSN00318850 | –10.6 | –10.7 | 276 | UMHSN00084548 | –10.5 | –10.6 |
| 226 | UMHSN00336354 | –9.8 | –10.7 | 277 | UMHSN00169963 | –10.8 | –10.6 |
| 227 | UMHSN00342018 | –10.5 | –10.7 | 278 | UMHSN00244941 | –10.6 | –10.6 |
| 228 | UMHSN00383584 | –10.7 | –10.7 | 279 | UMHSN00330614 | –9.7 | –10.6 |
| 229 | UMHSN00009895 | –9.8 | –10.7 | 280 | UMHSN00008157 | –10.5 | –10.6 |
| 230 | UMHSN00092866 | –10.5 | –10.7 | 281 | UMHSN00008556 | –10.5 | –10.6 |
| 231 | UMHSN00366902 | –10.7 | –10.7 | 282 | UMHSN00013286 | –9.4 | –10.6 |
| 232 | UMHSN00008335 | –10.7 | –10.7 | 283 | UMHSN00022500 | –9.4 | –10.6 |
| 233 | UMHSN00081048 | –10.4 | –10.7 | 284 | UMHSN00059350 | –10.6 | –10.6 |
| 234 | UMHSN00081546 | –10.6 | –10.7 | 285 | UMHSN00059532 | –10.1 | –10.6 |
| 235 | UMHSN00091498 | –10.6 | –10.7 | 286 | UMHSN00087720 | –10.5 | –10.6 |
| 236 | UMHSN00151984 | –10.4 | –10.7 | 287 | UMHSN00236373 | –10.5 | –10.6 |
| 237 | UMHSN00153950 | –10.0 | –10.7 | 288 | UMHSN00260534 | –10.6 | –10.6 |
| 238 | UMHSN00263341 | –10.7 | –10.7 | 289 | UMHSN00329053 | –10.6 | –10.6 |
| 239 | UMHSN00341811 | –10.4 | –10.7 | 290 | UMHSN00007116 | –10.4 | –10.6 |
| 240 | UMHSN00081544 | –10.2 | –10.7 | 291 | UMHSN00062422 | –10.3 | –10.6 |
| 241 | UMHSN00130521 | –10.4 | –10.7 | 292 | UMHSN00062775 | –10.0 | –10.6 |
| 242 | UMHSN00227821 | –10.5 | –10.7 | 293 | UMHSN00098733 | –10.5 | –10.6 |
| 243 | UMHSN00288287 | –10.6 | –10.7 | 294 | UMHSN00105115 | –10.2 | –10.6 |
| 244 | UMHSN00310526 | –10.7 | –10.7 | 295 | UMHSN00308859 | –10.6 | –10.6 |
| 245 | UMHSN00082747 | –10.6 | –10.6 | 296 | UMHSN00342832 | –10.5 | –10.6 |
| 246 | UMHSN00087820 | –10.6 | –10.6 | 297 | UMHSN00005268 | –10.5 | –10.6 |
| 247 | UMHSN00008854 | –10.4 | –10.6 | 298 | UMHSN00016037 | –9.9 | –10.6 |
| 248 | UMHSN00009049 | –10.5 | –10.6 | 299 | UMHSN00060276 | –9.9 | –10.6 |
| 249 | UMHSN00014025 | –10.5 | –10.6 | 300 | UMHSN00081081 | –10.4 | –10.6 |
| 250 | UMHSN00019643 | –10.6 | –10.6 | 301 | UMHSN00088967 | –11.4 | –10.6 |
| 251 | UMHSN00057413 | –10.6 | –10.6 | 302 | UMHSN00352569 | –9.9 | –10.6 |
| 252 | UMHSN00264160 | –10.5 | –10.6 | 303 | UMHSN00361740 | –10.6 | –10.6 |

| No. | SuperNatural II Code | Docking Score (kcal/mol) | | No. | SuperNatural II Code | Docking Score (kcal/mol) | |
| --- | --- | --- | --- | --- | --- | --- | --- |
|  |  | Std.^b^ | Mod.^c^ |  |  | Std.^b^ | Mod.^c^ |
| 304 | UMHSN00005177 | –9.4 | –10.6 | 355 | UMHSN00010271 | –10.5 | –10.5 |
| 305 | UMHSN00008214 | –10.5 | –10.6 | 356 | UMHSN00030737 | –9.6 | –10.5 |
| 306 | UMHSN00008382 | –11.1 | –10.6 | 357 | UMHSN00081558 | –10.4 | –10.5 |
| 307 | UMHSN00008800 | –10.5 | –10.6 | 358 | UMHSN00089693 | –10.5 | –10.5 |
| 308 | UMHSN00009059 | –10.5 | –10.6 | 359 | UMHSN00096723 | –9.6 | –10.5 |
| 309 | UMHSN00013285 | –9.5 | –10.6 | 360 | UMHSN00151590 | –9.9 | –10.5 |
| 310 | UMHSN00053405 | –10.4 | –10.6 | 361 | UMHSN00317979 | –10.4 | –10.5 |
| 311 | UMHSN00054768 | –10.5 | –10.6 | 362 | UMHSN00009939 | –10.4 | –10.5 |
| 312 | UMHSN00057168 | –10.5 | –10.6 | 363 | UMHSN00055512 | –9.6 | –10.5 |
| 313 | UMHSN00082732 | –11.1 | –10.6 | 364 | UMHSN00062722 | –9.5 | –10.5 |
| 314 | UMHSN00091401 | –10.5 | –10.6 | 365 | UMHSN00080923 | –10.4 | –10.5 |
| 315 | UMHSN00112571 | –10.2 | –10.6 | 366 | UMHSN00081092 | –10.5 | –10.5 |
| 316 | UMHSN00115247 | –10.5 | –10.6 | 367 | UMHSN00084341 | –10.4 | –10.5 |
| 317 | UMHSN00234454 | –9.8 | –10.6 | 368 | UMHSN00089397 | –10.5 | –10.5 |
| 318 | UMHSN00010230 | –10.6 | –10.6 | 369 | UMHSN00110070 | –9.9 | –10.5 |
| 319 | UMHSN00018045 | –9.4 | –10.6 | 370 | UMHSN00148371 | –10.3 | –10.5 |
| 320 | UMHSN00025039 | –10.0 | –10.6 | 371 | UMHSN00170518 | –10.3 | –10.5 |
| 321 | UMHSN00078033 | –10.4 | –10.6 | 372 | UMHSN00261116 | –10.5 | –10.5 |
| 322 | UMHSN00080972 | –9.5 | –10.6 | 373 | UMHSN00262800 | –10.2 | –10.5 |
| 323 | UMHSN00081096 | –10.6 | –10.6 | 374 | UMHSN00007058 | –10.3 | –10.5 |
| 324 | UMHSN00271512 | –10.5 | –10.6 | 375 | UMHSN00009786 | –9.7 | –10.5 |
| 325 | UMHSN00291822 | –10.6 | –10.6 | 376 | UMHSN00009824 | –10.5 | –10.5 |
| 326 | UMHSN00294502 | –10.5 | –10.6 | 377 | UMHSN00011776 | –10.3 | –10.5 |
| 327 | UMHSN00324042 | –10.6 | –10.6 | 378 | UMHSN00078343 | –10.5 | –10.5 |
| 328 | UMHSN00371674 | –10.4 | –10.6 | 379 | UMHSN00081933 | –10.5 | –10.5 |
| 329 | UMHSN00004199 | –10.6 | –10.6 | 380 | UMHSN00122260 | –9.6 | –10.5 |
| 330 | UMHSN00008769 | –10.5 | –10.6 | 381 | UMHSN00162872 | –10.5 | –10.5 |
| 331 | UMHSN00010257 | –10.3 | –10.6 | 382 | UMHSN00258938 | –10.5 | –10.5 |
| 332 | UMHSN00024014 | –10.5 | –10.6 | 383 | UMHSN00276176 | –10.3 | –10.5 |
| 333 | UMHSN00078865 | –10.6 | –10.6 | 384 | UMHSN00287471 | –10.0 | –10.5 |
| 334 | UMHSN00089817 | –10.4 | –10.6 | 385 | UMHSN00359393 | –10.5 | –10.5 |
| 335 | UMHSN00152431 | –10.3 | –10.6 | 386 | UMHSN00390267 | –9.8 | –10.5 |
| 336 | UMHSN00305204 | –10.5 | –10.6 | 387 | UMHSN00008412 | –10.5 | –10.5 |
| 337 | UMHSN00354461 | –10.5 | –10.6 | 388 | UMHSN00008446 | –10.5 | –10.5 |
| 338 | UMHSN00081699 | –10.0 | –10.5 | 389 | UMHSN00014904 | –10.8 | –10.5 |
| 339 | UMHSN00150297 | –10.5 | –10.5 | 390 | UMHSN00018206 | –9.8 | –10.5 |
| 340 | UMHSN00008439 | –10.5 | –10.5 | 391 | UMHSN00055057 | –9.7 | –10.5 |
| 341 | UMHSN00011723 | –10.5 | –10.5 | 392 | UMHSN00059753 | –10.4 | –10.5 |
| 342 | UMHSN00054061 | –10.5 | –10.5 | 393 | UMHSN00083519 | –10.4 | –10.5 |
| 343 | UMHSN00080062 | –10.3 | –10.5 | 394 | UMHSN00104653 | –9.6 | –10.5 |
| 344 | UMHSN00082210 | –10.3 | –10.5 | 395 | UMHSN00126637 | –9.9 | –10.5 |
| 345 | UMHSN00152286 | –10.3 | –10.5 | 396 | UMHSN00232972 | –10.5 | –10.5 |
| 346 | UMHSN00162447 | –10.5 | –10.5 | 397 | UMHSN00322584 | –10.5 | –10.5 |
| 347 | UMHSN00227072 | –10.5 | –10.5 | 398 | UMHSN00009909 | –9.6 | –10.5 |
| 348 | UMHSN00332256 | –10.5 | –10.5 | 399 | UMHSN00024187 | –10.2 | –10.5 |
| 349 | UMHSN00061021 | –10.2 | –10.5 | 400 | UMHSN00081542 | –10.4 | –10.5 |
| 350 | UMHSN00062283 | –10.3 | –10.5 | 401 | UMHSN00115563 | –10.4 | –10.5 |
| 351 | UMHSN00081911 | –10.3 | –10.5 | 402 | UMHSN00291936 | –10.4 | –10.5 |
| 352 | UMHSN00095412 | –10.4 | –10.5 | 403 | UMHSN00377569 | –10.4 | –10.5 |
| 353 | UMHSN00150281 | –10.4 | –10.5 | 404 | UMHSN00393134 | –10.4 | –10.5 |
| 354 | UMHSN00342220 | –10.5 | –10.5 | 405 | UMHSN00427090 | –9.5 | –10.5 |

| No. | SuperNatural II Code | Docking Score (kcal/mol) | | No. | SuperNatural II Code | Docking Score (kcal/mol) | |
| --- | --- | --- | --- | --- | --- | --- | --- |
|  |  | Std.^b^ | Mod.^c^ |  |  | Std.^b^ | Mod.^c^ |
| 406 | UMHSN00004350 | –10.4 | –10.5 | 457 | UMHSN00083318 | –9.4 | –10.4 |
| 407 | UMHSN00005493 | –10.4 | –10.5 | 458 | UMHSN00104506 | –9.9 | –10.4 |
| 408 | UMHSN00010243 | –10.5 | –10.5 | 459 | UMHSN00136043 | –10.1 | –10.4 |
| 409 | UMHSN00010619 | –10.3 | –10.5 | 460 | UMHSN00150779 | –10.0 | –10.4 |
| 410 | UMHSN00012350 | –10.0 | –10.5 | 461 | UMHSN00261620 | –10.4 | –10.4 |
| 411 | UMHSN00012651 | –10.4 | –10.5 | 462 | UMHSN00315928 | –10.0 | –10.4 |
| 412 | UMHSN00014931 | –9.9 | –10.5 | 463 | UMHSN00005495 | –10.3 | –10.4 |
| 413 | UMHSN00015021 | –10.4 | –10.5 | 464 | UMHSN00006440 | –10.4 | –10.4 |
| 414 | UMHSN00039391 | –10.5 | –10.5 | 465 | UMHSN00009197 | –10.4 | –10.4 |
| 415 | UMHSN00062549 | –10.0 | –10.5 | 466 | UMHSN00010229 | –9.4 | –10.4 |
| 416 | UMHSN00063284 | –10.1 | –10.5 | 467 | UMHSN00014792 | –10.4 | –10.4 |
| 417 | UMHSN00084670 | –9.9 | –10.5 | 468 | UMHSN00016059 | –9.8 | –10.4 |
| 418 | UMHSN00089702 | –9.9 | –10.5 | 469 | UMHSN00061854 | –9.6 | –10.4 |
| 419 | UMHSN00150780 | –10.4 | –10.5 | 470 | UMHSN00075994 | –9.6 | –10.4 |
| 420 | UMHSN00152529 | –10.4 | –10.5 | 471 | UMHSN00080996 | –10.6 | –10.4 |
| 421 | UMHSN00220485 | –10.4 | –10.5 | 472 | UMHSN00099210 | –10.4 | –10.4 |
| 422 | UMHSN00284552 | –10.4 | –10.5 | 473 | UMHSN00132224 | –10.4 | –10.4 |
| 423 | UMHSN00010580 | –10.4 | –10.5 | 474 | UMHSN00134455 | –10.4 | –10.4 |
| 424 | UMHSN00081778 | –10.6 | –10.5 | 475 | UMHSN00235834 | –10.4 | –10.4 |
| 425 | UMHSN00089319 | –10.0 | –10.5 | 476 | UMHSN00266629 | –10.6 | –10.4 |
| 426 | UMHSN00245142 | –10.4 | –10.5 | 477 | UMHSN00004178 | –9.5 | –10.4 |
| 427 | UMHSN00277342 | –10.4 | –10.5 | 478 | UMHSN00008334 | –10.4 | –10.4 |
| 428 | UMHSN00008305 | –10.4 | –10.4 | 479 | UMHSN00009866 | –10.3 | –10.4 |
| 429 | UMHSN00009828 | –9.8 | –10.4 | 480 | UMHSN00012150 | –10.1 | –10.4 |
| 430 | UMHSN00010233 | –10.4 | –10.4 | 481 | UMHSN00058022 | –10.3 | –10.4 |
| 431 | UMHSN00014833 | –10.4 | –10.4 | 482 | UMHSN00059026 | –10.4 | –10.4 |
| 432 | UMHSN00081610 | –10.1 | –10.4 | 483 | UMHSN00082643 | –10.2 | –10.4 |
| 433 | UMHSN00083554 | –10.4 | –10.4 | 484 | UMHSN00106505 | –9.8 | –10.4 |
| 434 | UMHSN00160252 | –10.2 | –10.4 | 485 | UMHSN00154987 | –9.9 | –10.4 |
| 435 | UMHSN00276010 | –10.4 | –10.4 | 486 | UMHSN00270428 | –10.3 | –10.4 |
| 436 | UMHSN00313746 | –10.4 | –10.4 | 487 | UMHSN00012310 | –10.3 | –10.4 |
| 437 | UMHSN00392413 | –9.4 | –10.4 | 488 | UMHSN00014603 | –10.4 | –10.4 |
| 438 | UMHSN00004149 | –9.5 | –10.4 | 489 | UMHSN00016060 | –10.4 | –10.4 |
| 439 | UMHSN00004208 | –10.4 | –10.4 | 490 | UMHSN00062347 | –9.5 | –10.4 |
| 440 | UMHSN00008705 | –10.3 | –10.4 | 491 | UMHSN00085139 | –9.4 | –10.4 |
| 441 | UMHSN00054609 | –10.1 | –10.4 | 492 | UMHSN00103395 | –10.5 | –10.4 |
| 442 | UMHSN00071746 | –9.8 | –10.4 | 493 | UMHSN00150515 | –10.3 | –10.4 |
| 443 | UMHSN00080506 | –10.4 | –10.4 | 494 | UMHSN00234102 | –10.4 | –10.4 |
| 444 | UMHSN00112224 | –10.4 | –10.4 | 495 | UMHSN00266817 | –10.3 | –10.4 |
| 445 | UMHSN00151664 | –10.3 | –10.4 | 496 | UMHSN00384898 | –10.2 | –10.4 |
| 446 | UMHSN00282429 | –10.4 | –10.4 | 497 | UMHSN00004198 | –10.3 | –10.4 |
| 447 | UMHSN00390872 | –10.4 | –10.4 | 498 | UMHSN00065946 | –10.2 | –10.4 |
| 448 | UMHSN00400695 | –10.3 | –10.4 | 499 | UMHSN00075849 | –10.3 | –10.4 |
| 449 | UMHSN00004193 | –10.3 | –10.4 | 500 | UMHSN00076851 | –10.4 | –10.4 |
| 450 | UMHSN00009722 | –10.5 | –10.4 | 501 | UMHSN00080552 | –10.7 | –10.4 |
| 451 | UMHSN00010814 | –9.4 | –10.4 | 502 | UMHSN00081692 | –9.6 | –10.4 |
| 452 | UMHSN00014830 | –10.4 | –10.4 | 503 | UMHSN00081703 | –10.3 | –10.4 |
| 453 | UMHSN00014891 | –10.4 | –10.4 | 504 | UMHSN00095376 | –10.3 | –10.4 |
| 454 | UMHSN00014908 | –10.4 | –10.4 | 505 | UMHSN00161213 | –10.2 | –10.4 |
| 455 | UMHSN00036609 | –10.0 | –10.4 | 506 | UMHSN00328259 | –10.4 | –10.4 |
| 456 | UMHSN00063785 | –10.2 | –10.4 | 507 | UMHSN00014809 | –10.4 | –10.4 |

| No. | SuperNatural II Code | Docking Score (kcal/mol) | | No. | SuperNatural II Code | Docking Score (kcal/mol) | |
| --- | --- | --- | --- | --- | --- | --- | --- |
|  |  | Std.^b^ | Mod.^c^ |  |  | Std.^b^ | Mod.^c^ |
| 508 | UMHSN00056737 | –10.2 | –10.4 | 559 | UMHSN00153655 | –10.2 | –10.3 |
| 509 | UMHSN00062082 | –9.7 | –10.4 | 560 | UMHSN00004626 | –10.3 | –10.3 |
| 510 | UMHSN00080882 | –10.1 | –10.4 | 561 | UMHSN00009831 | –10.0 | –10.3 |
| 511 | UMHSN00085043 | –10.2 | –10.4 | 562 | UMHSN00010821 | –10.3 | –10.3 |
| 512 | UMHSN00152497 | –9.5 | –10.4 | 563 | UMHSN00031411 | –10.1 | –10.3 |
| 513 | UMHSN00162729 | –10.4 | –10.4 | 564 | UMHSN00037285 | –10.2 | –10.3 |
| 514 | UMHSN00169518 | –10.3 | –10.4 | 565 | UMHSN00059255 | –10.3 | –10.3 |
| 515 | UMHSN00236313 | –10.1 | –10.4 | 566 | UMHSN00065449 | –10.3 | –10.3 |
| 516 | UMHSN00013390 | –10.3 | –10.4 | 567 | UMHSN00078937 | –10.2 | –10.3 |
| 517 | UMHSN00082499 | –10.3 | –10.4 | 568 | UMHSN00089684 | –9.6 | –10.3 |
| 518 | UMHSN00154647 | –10.3 | –10.4 | 569 | UMHSN00155107 | –10.3 | –10.3 |
| 519 | UMHSN00236755 | –10.4 | –10.4 | 570 | UMHSN00169145 | –9.8 | –10.3 |
| 520 | UMHSN00356618 | –9.4 | –10.4 | 571 | UMHSN00245464 | –10.3 | –10.3 |
| 521 | UMHSN00364850 | –10.3 | –10.4 | 572 | UMHSN00272979 | –9.6 | –10.3 |
| 522 | UMHSN00009185 | –10.3 | –10.4 | 573 | UMHSN00339991 | –10.1 | –10.3 |
| 523 | UMHSN00010227 | –10.3 | –10.4 | 574 | UMHSN00003980 | –10.3 | –10.3 |
| 524 | UMHSN00012260 | –10.3 | –10.4 | 575 | UMHSN00005182 | –10.3 | –10.3 |
| 525 | UMHSN00012262 | –10.3 | –10.4 | 576 | UMHSN00014816 | –10.3 | –10.3 |
| 526 | UMHSN00052183 | –9.5 | –10.4 | 577 | UMHSN00024181 | –10.3 | –10.3 |
| 527 | UMHSN00054923 | –9.4 | –10.4 | 578 | UMHSN00038667 | –9.9 | –10.3 |
| 528 | UMHSN00076485 | –10.3 | –10.4 | 579 | UMHSN00054569 | –10.2 | –10.3 |
| 529 | UMHSN00080970 | –10.3 | –10.4 | 580 | UMHSN00056157 | –10.2 | –10.3 |
| 530 | UMHSN00082686 | –9.7 | –10.4 | 581 | UMHSN00058403 | –10.2 | –10.3 |
| 531 | UMHSN00152428 | –9.4 | –10.4 | 582 | UMHSN00081750 | –10.3 | –10.3 |
| 532 | UMHSN00154645 | –10.3 | –10.4 | 583 | UMHSN00101055 | –10.2 | –10.3 |
| 533 | UMHSN00427202 | –10.2 | –10.4 | 584 | UMHSN00104987 | –9.5 | –10.3 |
| 534 | UMHSN00004080 | –10.3 | –10.3 | 585 | UMHSN00300534 | –10.3 | –10.3 |
| 535 | UMHSN00008768 | –10.2 | –10.3 | 586 | UMHSN00008764 | –10.1 | –10.3 |
| 536 | UMHSN00009182 | –10.4 | –10.3 | 587 | UMHSN00014023 | –10.3 | –10.3 |
| 537 | UMHSN00010280 | –10.3 | –10.3 | 588 | UMHSN00021550 | –10.3 | –10.3 |
| 538 | UMHSN00011766 | –10.3 | –10.3 | 589 | UMHSN00021886 | –9.9 | –10.3 |
| 539 | UMHSN00012790 | –10.3 | –10.3 | 590 | UMHSN00048048 | –10.2 | –10.3 |
| 540 | UMHSN00014871 | –10.3 | –10.3 | 591 | UMHSN00053882 | –10.3 | –10.3 |
| 541 | UMHSN00014898 | –10.3 | –10.3 | 592 | UMHSN00054619 | –9.6 | –10.3 |
| 542 | UMHSN00014915 | –10.3 | –10.3 | 593 | UMHSN00058028 | –10.0 | –10.3 |
| 543 | UMHSN00067528 | –10.3 | –10.3 | 594 | UMHSN00080897 | –10.1 | –10.3 |
| 544 | UMHSN00078683 | –10.3 | –10.3 | 595 | UMHSN00216625 | –10.0 | –10.3 |
| 545 | UMHSN00078989 | –10.3 | –10.3 | 596 | UMHSN00259290 | –10.2 | –10.3 |
| 546 | UMHSN00151775 | –10.3 | –10.3 | 597 | UMHSN00318949 | –10.3 | –10.3 |
| 547 | UMHSN00163739 | –10.3 | –10.3 | 598 | UMHSN00369656 | –10.3 | –10.3 |
| 548 | UMHSN00293974 | –10.3 | –10.3 | 599 | UMHSN00009177 | –10.3 | –10.3 |
| 549 | UMHSN00349466 | –10.3 | –10.3 | 600 | UMHSN00010804 | –10.1 | –10.3 |
| 550 | UMHSN00007651 | –10.3 | –10.3 | 601 | UMHSN00013976 | –10.3 | –10.3 |
| 551 | UMHSN00009867 | –10.3 | –10.3 | 602 | UMHSN00014837 | –10.2 | –10.3 |
| 552 | UMHSN00019986 | –10.1 | –10.3 | 603 | UMHSN00014855 | –10.3 | –10.3 |
| 553 | UMHSN00054680 | –10.3 | –10.3 | 604 | UMHSN00016030 | –10.3 | –10.3 |
| 554 | UMHSN00058724 | –10.3 | –10.3 | 605 | UMHSN00058086 | –10.5 | –10.3 |
| 555 | UMHSN00059116 | –10.2 | –10.3 | 606 | UMHSN00060278 | –10.1 | –10.3 |
| 556 | UMHSN00074793 | –10.3 | –10.3 | 607 | UMHSN00080535 | –10.3 | –10.3 |
| 557 | UMHSN00074913 | –10.0 | –10.3 | 608 | UMHSN00083486 | –9.6 | –10.3 |
| 558 | UMHSN00080001 | –10.3 | –10.3 | 609 | UMHSN00163683 | –10.0 | –10.3 |

| No. | SuperNatural II Code | Docking Score (kcal/mol) | | No. | SuperNatural II Code | Docking Score (kcal/mol) | |
| --- | --- | --- | --- | --- | --- | --- | --- |
|  |  | Std.^b^ | Mod.^c^ |  |  | Std.^b^ | Mod.^c^ |
| 610 | UMHSN00169255 | –10.1 | –10.3 | 661 | UMHSN00053596 | –10.3 | –10.3 |
| 611 | UMHSN00241255 | –9.9 | –10.3 | 662 | UMHSN00073863 | –10.2 | –10.3 |
| 612 | UMHSN00259938 | –10.3 | –10.3 | 663 | UMHSN00078689 | –10.2 | –10.3 |
| 613 | UMHSN00262255 | –10.3 | –10.3 | 664 | UMHSN00081902 | –10.3 | –10.3 |
| 614 | UMHSN00293449 | –10.2 | –10.3 | 665 | UMHSN00282614 | –10.3 | –10.3 |
| 615 | UMHSN00376828 | –9.7 | –10.3 | 666 | UMHSN00347466 | –9.6 | –10.3 |
| 616 | UMHSN00400707 | –10.3 | –10.3 | 667 | UMHSN00377404 | –10.2 | –10.3 |
| 617 | UMHSN00008367 | –10.3 | –10.3 | 668 | UMHSN00379515 | –9.4 | –10.3 |
| 618 | UMHSN00009359 | –10.1 | –10.3 | 669 | UMHSN00427203 | –10.1 | –10.3 |
| 619 | UMHSN00009726 | –10.3 | –10.3 | 670 | UMHSN00008375 | –10.2 | –10.3 |
| 620 | UMHSN00009947 | –10.0 | –10.3 | 671 | UMHSN00042130 | –10.3 | –10.3 |
| 621 | UMHSN00010273 | –10.3 | –10.3 | 672 | UMHSN00058427 | –9.5 | –10.3 |
| 622 | UMHSN00014773 | –10.3 | –10.3 | 673 | UMHSN00103886 | –10.2 | –10.3 |
| 623 | UMHSN00014845 | –10.3 | –10.3 | 674 | UMHSN00152527 | –10.2 | –10.3 |
| 624 | UMHSN00053385 | –10.1 | –10.3 | 675 | UMHSN00250818 | –9.9 | –10.3 |
| 625 | UMHSN00054954 | –9.4 | –10.3 | 676 | UMHSN00314036 | –10.3 | –10.3 |
| 626 | UMHSN00076673 | –10.4 | –10.3 | 677 | UMHSN00318990 | –9.9 | –10.3 |
| 627 | UMHSN00081901 | –10.1 | –10.3 | 678 | UMHSN00366768 | –9.4 | –10.3 |
| 628 | UMHSN00081930 | –10.1 | –10.3 | 679 | UMHSN00007942 | –10.2 | –10.2 |
| 629 | UMHSN00088803 | –10.3 | –10.3 | 680 | UMHSN00015860 | –10.1 | –10.2 |
| 630 | UMHSN00097200 | –10.9 | –10.3 | 681 | UMHSN00016965 | –10.1 | –10.2 |
| 631 | UMHSN00154022 | –9.9 | –10.3 | 682 | UMHSN00018112 | –9.7 | –10.2 |
| 632 | UMHSN00252934 | –10.3 | –10.3 | 683 | UMHSN00065936 | –10.1 | –10.2 |
| 633 | UMHSN00315168 | –9.7 | –10.3 | 684 | UMHSN00078753 | –10.2 | –10.2 |
| 634 | UMHSN00334868 | –10.4 | –10.3 | 685 | UMHSN00080395 | –9.6 | –10.2 |
| 635 | UMHSN00336044 | –10.3 | –10.3 | 686 | UMHSN00081797 | –10.2 | –10.2 |
| 636 | UMHSN00380141 | –10.3 | –10.3 | 687 | UMHSN00112490 | –10.0 | –10.2 |
| 637 | UMHSN00401033 | –10.2 | –10.3 | 688 | UMHSN00127202 | –10.2 | –10.2 |
| 638 | UMHSN00007959 | –10.2 | –10.3 | 689 | UMHSN00150299 | –10.1 | –10.2 |
| 639 | UMHSN00009222 | –10.3 | –10.3 | 690 | UMHSN00150742 | –10.1 | –10.2 |
| 640 | UMHSN00009840 | –10.3 | –10.3 | 691 | UMHSN00155105 | –10.2 | –10.2 |
| 641 | UMHSN00019723 | –10.1 | –10.3 | 692 | UMHSN00313946 | –10.2 | –10.2 |
| 642 | UMHSN00077938 | –10.1 | –10.3 | 693 | UMHSN00357127 | –10.2 | –10.2 |
| 643 | UMHSN00080521 | –9.9 | –10.3 | 694 | UMHSN00005414 | –10.2 | –10.2 |
| 644 | UMHSN00080947 | –9.9 | –10.3 | 695 | UMHSN00009981 | –10.1 | –10.2 |
| 645 | UMHSN00081793 | –10.0 | –10.3 | 696 | UMHSN00013127 | –9.6 | –10.2 |
| 646 | UMHSN00087158 | –10.0 | –10.3 | 697 | UMHSN00014777 | –10.2 | –10.2 |
| 647 | UMHSN00090417 | –9.5 | –10.3 | 698 | UMHSN00014914 | –10.2 | –10.2 |
| 648 | UMHSN00112122 | –9.5 | –10.3 | 699 | UMHSN00015792 | –10.3 | –10.2 |
| 649 | UMHSN00115657 | –10.2 | –10.3 | 700 | UMHSN00018221 | –9.9 | –10.2 |
| 650 | UMHSN00150741 | –9.5 | –10.3 | 701 | UMHSN00019722 | –10.9 | –10.2 |
| 651 | UMHSN00152534 | –10.2 | –10.3 | 702 | UMHSN00056095 | –10.2 | –10.2 |
| 652 | UMHSN00161391 | –9.9 | –10.3 | 703 | UMHSN00078737 | –9.7 | –10.2 |
| 653 | UMHSN00272941 | –10.2 | –10.3 | 704 | UMHSN00079231 | –10.1 | –10.2 |
| 654 | UMHSN00311214 | –10.3 | –10.3 | 705 | UMHSN00081742 | –10.1 | –10.2 |
| 655 | UMHSN00350891 | –10.3 | –10.3 | 706 | UMHSN00082783 | –10.2 | –10.2 |
| 656 | UMHSN00004214 | –10.3 | –10.3 | 707 | UMHSN00091329 | –10.2 | –10.2 |
| 657 | UMHSN00009221 | –10.3 | –10.3 | 708 | UMHSN00096738 | –10.2 | –10.2 |
| 658 | UMHSN00009764 | –10.2 | –10.3 | 709 | UMHSN00098581 | –10.1 | –10.2 |
| 659 | UMHSN00018582 | –10.2 | –10.3 | 710 | UMHSN00141409 | –10.2 | –10.2 |
| 660 | UMHSN00024036 | –10.2 | –10.3 | 711 | UMHSN00286847 | –10.0 | –10.2 |

| No. | SuperNatural II Code | Docking Score (kcal/mol) | | No. | SuperNatural II Code | Docking Score (kcal/mol) | |
| --- | --- | --- | --- | --- | --- | --- | --- |
|  |  | Std.^b^ | Mod.^c^ |  |  | Std.^b^ | Mod.^c^ |
| 712 | UMHSN00312429 | –9.9 | –10.2 | 763 | UMHSN00005510 | –9.9 | –10.2 |
| 713 | UMHSN00008598 | –10.1 | –10.2 | 764 | UMHSN00008342 | –10.4 | –10.2 |
| 714 | UMHSN00014019 | –10.1 | –10.2 | 765 | UMHSN00008343 | –10.2 | –10.2 |
| 715 | UMHSN00015058 | –10.2 | –10.2 | 766 | UMHSN00008454 | –10.2 | –10.2 |
| 716 | UMHSN00015160 | –10.2 | –10.2 | 767 | UMHSN00014360 | –10.0 | –10.2 |
| 717 | UMHSN00044921 | –10.2 | –10.2 | 768 | UMHSN00015050 | –10.2 | –10.2 |
| 718 | UMHSN00055426 | –10.1 | –10.2 | 769 | UMHSN00015606 | –9.4 | –10.2 |
| 719 | UMHSN00081762 | –10.2 | –10.2 | 770 | UMHSN00071723 | –10.1 | –10.2 |
| 720 | UMHSN00097854 | –10.0 | –10.2 | 771 | UMHSN00079087 | –9.6 | –10.2 |
| 721 | UMHSN00099060 | –10.2 | –10.2 | 772 | UMHSN00079600 | –10.2 | –10.2 |
| 722 | UMHSN00099769 | –10.2 | –10.2 | 773 | UMHSN00084690 | –10.1 | –10.2 |
| 723 | UMHSN00126638 | –10.1 | –10.2 | 774 | UMHSN00089692 | –9.5 | –10.2 |
| 724 | UMHSN00241474 | –10.2 | –10.2 | 775 | UMHSN00094039 | –10.0 | –10.2 |
| 725 | UMHSN00291742 | –10.2 | –10.2 | 776 | UMHSN00114039 | –9.7 | –10.2 |
| 726 | UMHSN00311512 | –10.2 | –10.2 | 777 | UMHSN00139914 | –9.7 | –10.2 |
| 727 | UMHSN00315352 | –9.5 | –10.2 | 778 | UMHSN00149700 | –10.1 | –10.2 |
| 728 | UMHSN00372439 | –10.1 | –10.2 | 779 | UMHSN00162546 | –10.0 | –10.2 |
| 729 | UMHSN00004385 | –10.2 | –10.2 | 780 | UMHSN00225267 | –10.1 | –10.2 |
| 730 | UMHSN00009900 | –9.7 | –10.2 | 781 | UMHSN00259255 | –10.1 | –10.2 |
| 731 | UMHSN00011752 | –10.2 | –10.2 | 782 | UMHSN00299051 | –10.1 | –10.2 |
| 732 | UMHSN00014776 | –10.2 | –10.2 | 783 | UMHSN00008310 | –10.2 | –10.2 |
| 733 | UMHSN00017284 | –10.0 | –10.2 | 784 | UMHSN00025090 | –10.0 | –10.2 |
| 734 | UMHSN00048460 | –10.2 | –10.2 | 785 | UMHSN00058199 | –10.2 | –10.2 |
| 735 | UMHSN00049926 | –10.1 | –10.2 | 786 | UMHSN00081577 | –9.5 | –10.2 |
| 736 | UMHSN00101338 | –10.2 | –10.2 | 787 | UMHSN00081992 | –10.1 | –10.2 |
| 737 | UMHSN00137005 | –10.2 | –10.2 | 788 | UMHSN00083989 | –9.9 | –10.2 |
| 738 | UMHSN00163009 | –10.1 | –10.2 | 789 | UMHSN00087086 | –9.6 | –10.2 |
| 739 | UMHSN00169139 | –10.2 | –10.2 | 790 | UMHSN00150510 | –10.1 | –10.2 |
| 740 | UMHSN00259915 | –10.0 | –10.2 | 791 | UMHSN00163694 | –10.1 | –10.2 |
| 741 | UMHSN00262785 | –10.2 | –10.2 | 792 | UMHSN00166592 | –9.5 | –10.2 |
| 742 | UMHSN00312834 | –10.1 | –10.2 | 793 | UMHSN00265970 | –10.2 | –10.2 |
| 743 | UMHSN00381468 | –10.1 | –10.2 | 794 | UMHSN00285411 | –10.1 | –10.2 |
| 744 | UMHSN00404118 | –10.2 | –10.2 | 795 | UMHSN00303681 | –10.1 | –10.2 |
| 745 | UMHSN00427112 | –10.1 | –10.2 | 796 | UMHSN00004143 | –10.1 | –10.2 |
| 746 | UMHSN00007744 | –9.6 | –10.2 | 797 | UMHSN00012315 | –10.1 | –10.2 |
| 747 | UMHSN00009357 | –9.4 | –10.2 | 798 | UMHSN00015951 | –10.1 | –10.2 |
| 748 | UMHSN00009358 | –10.2 | –10.2 | 799 | UMHSN00031417 | –9.4 | –10.2 |
| 749 | UMHSN00038355 | –10.2 | –10.2 | 800 | UMHSN00057220 | –10.2 | –10.2 |
| 750 | UMHSN00049091 | –9.9 | –10.2 | 801 | UMHSN00058185 | –10.1 | –10.2 |
| 751 | UMHSN00057899 | –10.1 | –10.2 | 802 | UMHSN00066756 | –10.2 | –10.2 |
| 752 | UMHSN00061715 | –10.0 | –10.2 | 803 | UMHSN00068712 | –10.1 | –10.2 |
| 753 | UMHSN00072825 | –10.1 | –10.2 | 804 | UMHSN00080789 | –10.1 | –10.2 |
| 754 | UMHSN00080831 | –10.1 | –10.2 | 805 | UMHSN00082649 | –10.0 | –10.2 |
| 755 | UMHSN00095995 | –10.1 | –10.2 | 806 | UMHSN00154948 | –10.1 | –10.2 |
| 756 | UMHSN00135484 | –9.9 | –10.2 | 807 | UMHSN00163102 | –10.1 | –10.2 |
| 757 | UMHSN00264626 | –10.3 | –10.2 | 808 | UMHSN00214786 | –10.1 | –10.2 |
| 758 | UMHSN00308295 | –10.2 | –10.2 | 809 | UMHSN00359832 | –10.0 | –10.2 |
| 759 | UMHSN00358573 | –10.2 | –10.2 | 810 | UMHSN00004202 | –10.0 | –10.2 |
| 760 | UMHSN00365714 | –10.0 | –10.2 | 811 | UMHSN00004628 | –10.1 | –10.2 |
| 761 | UMHSN00394934 | –10.1 | –10.2 | 812 | UMHSN00005073 | –10.0 | –10.2 |
| 762 | UMHSN00427207 | –9.5 | –10.2 | 813 | UMHSN00008415 | –10.2 | –10.2 |

| No. | SuperNatural II Code | Docking Score (kcal/mol) | | No. | SuperNatural II Code | Docking Score (kcal/mol) | |
| --- | --- | --- | --- | --- | --- | --- | --- |
|  |  | Std.^b^ | Mod.^c^ |  |  | Std.^b^ | Mod.^c^ |
| 814 | UMHSN00009224 | –10.1 | –10.2 | 865 | UMHSN00352535 | –9.9 | –10.1 |
| 815 | UMHSN00009978 | –10.1 | –10.2 | 866 | UMHSN00354524 | –10.1 | –10.1 |
| 816 | UMHSN00052085 | –9.9 | –10.2 | 867 | UMHSN00361052 | –10.1 | –10.1 |
| 817 | UMHSN00053853 | –10.1 | –10.2 | 868 | UMHSN00370671 | –10.0 | –10.1 |
| 818 | UMHSN00056457 | –10.6 | –10.2 | 869 | UMHSN00005693 | –10.1 | –10.1 |
| 819 | UMHSN00059444 | –10.1 | –10.2 | 870 | UMHSN00008212 | –9.5 | –10.1 |
| 820 | UMHSN00063648 | –9.6 | –10.2 | 871 | UMHSN00008780 | –10.1 | –10.1 |
| 821 | UMHSN00076625 | –10.1 | –10.2 | 872 | UMHSN00010651 | –9.8 | –10.1 |
| 822 | UMHSN00080655 | –9.9 | –10.2 | 873 | UMHSN00012666 | –9.7 | –10.1 |
| 823 | UMHSN00088665 | –9.7 | –10.2 | 874 | UMHSN00014021 | –9.4 | –10.1 |
| 824 | UMHSN00090909 | –9.9 | –10.2 | 875 | UMHSN00014836 | –10.0 | –10.1 |
| 825 | UMHSN00091895 | –9.7 | –10.2 | 876 | UMHSN00035553 | –9.6 | –10.1 |
| 826 | UMHSN00097357 | –10.1 | –10.2 | 877 | UMHSN00058394 | –9.9 | –10.1 |
| 827 | UMHSN00099098 | –9.9 | –10.2 | 878 | UMHSN00061622 | –9.5 | –10.1 |
| 828 | UMHSN00099240 | –9.5 | –10.2 | 879 | UMHSN00063215 | –10.2 | –10.1 |
| 829 | UMHSN00108682 | –9.4 | –10.2 | 880 | UMHSN00082525 | –10.1 | –10.1 |
| 830 | UMHSN00150302 | –10.1 | –10.2 | 881 | UMHSN00088126 | –9.8 | –10.1 |
| 831 | UMHSN00277846 | –10.0 | –10.2 | 882 | UMHSN00097257 | –9.7 | –10.1 |
| 832 | UMHSN00316038 | –10.0 | –10.2 | 886 | UMHSN00109685 | –10.1 | –10.1 |
| 833 | UMHSN00333515 | –10.1 | –10.2 | 887 | UMHSN00113603 | –9.5 | –10.1 |
| 834 | UMHSN00346887 | –10.0 | –10.2 | 888 | UMHSN00129550 | –10.1 | –10.1 |
| 835 | UMHSN00009827 | –10.0 | –10.2 | 889 | UMHSN00150202 | –9.7 | –10.1 |
| 836 | UMHSN00010264 | –10.1 | –10.2 | 890 | UMHSN00150644 | –10.0 | –10.1 |
| 837 | UMHSN00016259 | –10.1 | –10.2 | 891 | UMHSN00154649 | –10.1 | –10.1 |
| 838 | UMHSN00021584 | –9.9 | –10.2 | 892 | UMHSN00173521 | –10.1 | –10.1 |
| 839 | UMHSN00022639 | –9.8 | –10.2 | 893 | UMHSN00265331 | –10.1 | –10.1 |
| 840 | UMHSN00035488 | –10.4 | –10.2 | 894 | UMHSN00326693 | –9.6 | –10.1 |
| 841 | UMHSN00059303 | –10.2 | –10.2 | 895 | UMHSN00427208 | –9.5 | –10.1 |
| 842 | UMHSN00067452 | –10.2 | –10.2 | 896 | UMHSN00004095 | –10.1 | –10.1 |
| 843 | UMHSN00104014 | –10.2 | –10.2 | 897 | UMHSN00008181 | –10.0 | –10.1 |
| 844 | UMHSN00153947 | –9.6 | –10.2 | 898 | UMHSN00008308 | –10.1 | –10.1 |
| 845 | UMHSN00255790 | –10.1 | –10.2 | 899 | UMHSN00013155 | –9.6 | –10.1 |
| 846 | UMHSN00362706 | –10.1 | –10.2 | 900 | UMHSN00013156 | –10.1 | –10.1 |
| 847 | UMHSN00008457 | –10.1 | –10.1 | 901 | UMHSN00015025 | –10.1 | –10.1 |
| 848 | UMHSN00009944 | –10.1 | –10.1 | 902 | UMHSN00081056 | –10.1 | –10.1 |
| 849 | UMHSN00010811 | –10.1 | –10.1 | 903 | UMHSN00108605 | –9.5 | –10.1 |
| 850 | UMHSN00013998 | –9.6 | –10.1 | 904 | UMHSN00152368 | –9.9 | –10.1 |
| 851 | UMHSN00021475 | –9.6 | –10.1 | 905 | UMHSN00265522 | –10.0 | –10.1 |
| 852 | UMHSN00052542 | –10.0 | –10.1 | 906 | UMHSN00335826 | –10.2 | –10.1 |
| 853 | UMHSN00056830 | –9.9 | –10.1 | 907 | UMHSN00427147 | –10.3 | –10.1 |
| 854 | UMHSN00077825 | –10.1 | –10.1 | 908 | UMHSN00004644 | –10.0 | –10.1 |
| 855 | UMHSN00094097 | –9.9 | –10.1 | 909 | UMHSN00008452 | –10.1 | –10.1 |
| 856 | UMHSN00097212 | –9.9 | –10.1 | 910 | UMHSN00009218 | –10.1 | –10.1 |
| 857 | UMHSN00116179 | –10.0 | –10.1 | 911 | UMHSN00014783 | –10.0 | –10.1 |
| 858 | UMHSN00150640 | –10.0 | –10.1 | 912 | UMHSN00035889 | –9.9 | –10.1 |
| 859 | UMHSN00150873 | –10.0 | –10.1 | 913 | UMHSN00062843 | –9.8 | –10.1 |
| 860 | UMHSN00154828 | –10.0 | –10.1 | 914 | UMHSN00063560 | –9.8 | –10.1 |
| 861 | UMHSN00169508 | –10.0 | –10.1 | 915 | UMHSN00063622 | –10.1 | –10.1 |
| 862 | UMHSN00230018 | –10.1 | –10.1 | 916 | UMHSN00081836 | –9.7 | –10.1 |
| 863 | UMHSN00237849 | –9.8 | –10.1 | 917 | UMHSN00168974 | –10.0 | –10.1 |
| 864 | UMHSN00276063 | –10.3 | –10.1 | 918 | UMHSN00271746 | –10.0 | –10.1 |

| No. | SuperNatural II Code | Docking Score (kcal/mol) | | No. | SuperNatural II Code | Docking Score (kcal/mol) | |
| --- | --- | --- | --- | --- | --- | --- | --- |
|  |  | Std.^b^ | Mod.^c^ |  |  | Std.^b^ | Mod.^c^ |
| 919 | UMHSN00369337 | –10.1 | –10.1 | 970 | UMHSN00063160 | –9.8 | –10.1 |
| 920 | UMHSN00007060 | –10.4 | –10.1 | 971 | UMHSN00066349 | –9.7 | –10.1 |
| 921 | UMHSN00009739 | –10.1 | –10.1 | 972 | UMHSN00071499 | –9.7 | –10.1 |
| 922 | UMHSN00019612 | –10.0 | –10.1 | 973 | UMHSN00073393 | –10.0 | –10.1 |
| 923 | UMHSN00022421 | –10.0 | –10.1 | 974 | UMHSN00080534 | –9.7 | –10.1 |
| 924 | UMHSN00022422 | –9.6 | –10.1 | 975 | UMHSN00081850 | –10.1 | –10.1 |
| 925 | UMHSN00054212 | –10.0 | –10.1 | 976 | UMHSN00101994 | –10.2 | –10.1 |
| 926 | UMHSN00058230 | –9.9 | –10.1 | 977 | UMHSN00111882 | –9.9 | –10.1 |
| 927 | UMHSN00058246 | –9.7 | –10.1 | 978 | UMHSN00145331 | –9.8 | –10.1 |
| 928 | UMHSN00085657 | –10.0 | –10.1 | 979 | UMHSN00151161 | –10.0 | –10.1 |
| 929 | UMHSN00091523 | –9.7 | –10.1 | 980 | UMHSN00174380 | –10.0 | –10.1 |
| 930 | UMHSN00102433 | –10.0 | –10.1 | 981 | UMHSN00238467 | –10.1 | –10.1 |
| 931 | UMHSN00141374 | –9.9 | –10.1 | 982 | UMHSN00267824 | –10.1 | –10.1 |
| 932 | UMHSN00148874 | –9.6 | –10.1 | 983 | UMHSN00297594 | –10.1 | –10.1 |
| 933 | UMHSN00150296 | –9.7 | –10.1 | 984 | UMHSN00318043 | –10.1 | –10.1 |
| 934 | UMHSN00155113 | –10.1 | –10.1 | 985 | UMHSN00004108 | –10.0 | –10.1 |
| 935 | UMHSN00176166 | –10.1 | –10.1 | 986 | UMHSN00004625 | –9.9 | –10.1 |
| 936 | UMHSN00241020 | –9.7 | –10.1 | 987 | UMHSN00004663 | –9.9 | –10.1 |
| 937 | UMHSN00008086 | –9.9 | –10.1 | 988 | UMHSN00008378 | –9.7 | –10.1 |
| 938 | UMHSN00008312 | –10.1 | –10.1 | 989 | UMHSN00008560 | –10.1 | –10.1 |
| 939 | UMHSN00009168 | –10.1 | –10.1 | 990 | UMHSN00009235 | –10.1 | –10.1 |
| 940 | UMHSN00009816 | –10.1 | –10.1 | 991 | UMHSN00009763 | –10.1 | –10.1 |
| 941 | UMHSN00010649 | –10.0 | –10.1 | 992 | UMHSN00018401 | –9.4 | –10.1 |
| 942 | UMHSN00014823 | –10.1 | –10.1 | 993 | UMHSN00055894 | –9.9 | –10.1 |
| 943 | UMHSN00014895 | –10.5 | –10.1 | 994 | UMHSN00055989 | –9.9 | –10.1 |
| 944 | UMHSN00015531 | –9.7 | –10.1 | 995 | UMHSN00062736 | –10.0 | –10.1 |
| 945 | UMHSN00031189 | –10.0 | –10.1 | 996 | UMHSN00073084 | –9.8 | –10.1 |
| 946 | UMHSN00063321 | –9.9 | –10.1 | 997 | UMHSN00076907 | –10.1 | –10.1 |
| 947 | UMHSN00072813 | –10.0 | –10.1 | 998 | UMHSN00081775 | –9.9 | –10.1 |
| 948 | UMHSN00079053 | –9.8 | –10.1 | 999 | UMHSN00084676 | –9.5 | –10.1 |
| 949 | UMHSN00080539 | –10.0 | –10.1 | 1000 | UMHSN00132837 | –10.1 | –10.1 |
| 950 | UMHSN00081635 | –10.9 | –10.1 | 1001 | UMHSN00237206 | –10.0 | –10.1 |
| 951 | UMHSN00083183 | –9.6 | –10.1 | 1002 | UMHSN00251881 | –10.1 | –10.1 |
| 952 | UMHSN00105302 | –9.7 | –10.1 | 1003 | UMHSN00259118 | –10.1 | –10.1 |
| 953 | UMHSN00106633 | –10.0 | –10.1 | 1004 | UMHSN00303214 | –9.4 | –10.1 |
| 954 | UMHSN00115356 | –10.1 | –10.1 | 1005 | UMHSN00318448 | –10.0 | –10.1 |
| 955 | UMHSN00152337 | –10.1 | –10.1 | 1006 | UMHSN00364163 | –10.1 | –10.1 |
| 956 | UMHSN00160628 | –9.9 | –10.1 | 1007 | UMHSN00365038 | –10.1 | –10.1 |
| 957 | UMHSN00161051 | –10.0 | –10.1 | 1008 | UMHSN00004341 | –9.9 | –10.1 |
| 958 | UMHSN00234858 | –10.1 | –10.1 | 1009 | UMHSN00007097 | –9.8 | –10.1 |
| 959 | UMHSN00253227 | –10.1 | –10.1 | 1010 | UMHSN00007962 | –9.8 | –10.1 |
| 960 | UMHSN00262453 | –10.1 | –10.1 | 1011 | UMHSN00008307 | –10.0 | –10.1 |
| 961 | UMHSN00004661 | –10.1 | –10.1 | 1012 | UMHSN00009711 | –10.0 | –10.1 |
| 962 | UMHSN00008303 | –10.1 | –10.1 | 1013 | UMHSN00009737 | –10.1 | –10.1 |
| 963 | UMHSN00008374 | –10.1 | –10.1 | 1014 | UMHSN00011022 | –10.0 | –10.1 |
| 964 | UMHSN00014829 | –10.1 | –10.1 | 1015 | UMHSN00011742 | –10.0 | –10.1 |
| 965 | UMHSN00014907 | –10.1 | –10.1 | 1016 | UMHSN00011743 | –9.8 | –10.1 |
| 966 | UMHSN00016618 | –9.4 | –10.1 | 1017 | UMHSN00012102 | –9.7 | –10.1 |
| 967 | UMHSN00017320 | –9.9 | –10.1 | 1018 | UMHSN00017050 | –9.9 | –10.1 |
| 968 | UMHSN00024011 | –10.0 | –10.1 | 1019 | UMHSN00017619 | –10.0 | –10.1 |
| 969 | UMHSN00061581 | –9.9 | –10.1 | 1020 | UMHSN00024013 | –10.0 | –10.1 |

| No. | SuperNatural II Code | Docking Score (kcal/mol) | | No. | SuperNatural II Code | Docking Score (kcal/mol) | |
| --- | --- | --- | --- | --- | --- | --- | --- |
|  |  | Std.^b^ | Mod.^c^ |  |  | Std.^b^ | Mod.^c^ |
| 1021 | UMHSN00032975 | –10.1 | –10.1 | 1072 | UMHSN00072467 | –9.7 | –10.0 |
| 1022 | UMHSN00068724 | –10.1 | –10.1 | 1073 | UMHSN00081108 | –9.8 | –10.0 |
| 1023 | UMHSN00076934 | –10.1 | –10.1 | 1074 | UMHSN00083383 | –10.0 | –10.0 |
| 1024 | UMHSN00097218 | –9.7 | –10.1 | 1075 | UMHSN00084322 | –9.9 | –10.0 |
| 1025 | UMHSN00144691 | –9.6 | –10.1 | 1076 | UMHSN00088685 | –10.0 | –10.0 |
| 1026 | UMHSN00152298 | –10.0 | –10.1 | 1077 | UMHSN00092480 | –10.0 | –10.0 |
| 1027 | UMHSN00152304 | –10.0 | –10.1 | 1078 | UMHSN00134602 | –9.9 | –10.0 |
| 1028 | UMHSN00152338 | –10.0 | –10.1 | 1079 | UMHSN00151203 | –9.4 | –10.0 |
| 1029 | UMHSN00160947 | –10.0 | –10.1 | 1080 | UMHSN00162347 | –9.9 | –10.0 |
| 1030 | UMHSN00235544 | –9.9 | –10.1 | 1081 | UMHSN00238404 | –10.0 | –10.0 |
| 1031 | UMHSN00259553 | –9.4 | –10.1 | 1082 | UMHSN00306316 | –10.0 | –10.0 |
| 1032 | UMHSN00260805 | –10.1 | –10.1 | 1083 | UMHSN00376019 | –9.9 | –10.0 |
| 1033 | UMHSN00354373 | –9.9 | –10.1 | 1084 | UMHSN00008373 | –10.0 | –10.0 |
| 1034 | UMHSN00355805 | –9.9 | –10.1 | 1085 | UMHSN00008392 | –10.0 | –10.0 |
| 1035 | UMHSN00383784 | –10.0 | –10.1 | 1086 | UMHSN00008398 | –10.0 | –10.0 |
| 1036 | UMHSN00004375 | –10.0 | –10.1 | 1087 | UMHSN00009176 | –10.0 | –10.0 |
| 1037 | UMHSN00004630 | –9.8 | –10.1 | 1088 | UMHSN00009519 | –10.0 | –10.0 |
| 1038 | UMHSN00009217 | –9.8 | –10.1 | 1089 | UMHSN00009894 | –10.0 | –10.0 |
| 1039 | UMHSN00014020 | –9.6 | –10.1 | 1090 | UMHSN00010611 | –10.1 | –10.0 |
| 1040 | UMHSN00014878 | –10.0 | –10.1 | 1091 | UMHSN00012121 | –10.0 | –10.0 |
| 1041 | UMHSN00015138 | –10.0 | –10.1 | 1092 | UMHSN00015883 | –10.0 | –10.0 |
| 1042 | UMHSN00016493 | –9.6 | –10.1 | 1093 | UMHSN00022175 | –9.7 | –10.0 |
| 1043 | UMHSN00075418 | –10.1 | –10.1 | 1094 | UMHSN00079014 | –9.4 | –10.0 |
| 1044 | UMHSN00076537 | –10.0 | –10.1 | 1095 | UMHSN00081780 | –10.0 | –10.0 |
| 1045 | UMHSN00084333 | –10.0 | –10.1 | 1096 | UMHSN00084342 | –10.0 | –10.0 |
| 1046 | UMHSN00086435 | –10.0 | –10.1 | 1097 | UMHSN00085375 | –9.8 | –10.0 |
| 1047 | UMHSN00089680 | –10.1 | –10.1 | 1098 | UMHSN00095847 | –9.5 | –10.0 |
| 1048 | UMHSN00090934 | –9.9 | –10.1 | 1099 | UMHSN00100222 | –9.9 | –10.0 |
| 1049 | UMHSN00135415 | –9.4 | –10.1 | 1100 | UMHSN00104003 | –9.8 | –10.0 |
| 1050 | UMHSN00154705 | –10.0 | –10.1 | 1101 | UMHSN00105308 | –9.7 | –10.0 |
| 1051 | UMHSN00229840 | –10.0 | –10.1 | 1102 | UMHSN00107548 | –10.0 | –10.0 |
| 1052 | UMHSN00241060 | –10.1 | –10.1 | 1103 | UMHSN00141060 | –10.0 | –10.0 |
| 1053 | UMHSN00270294 | –10.0 | –10.1 | 1104 | UMHSN00152317 | –9.5 | –10.0 |
| 1054 | UMHSN00275080 | –10.4 | –10.1 | 1105 | UMHSN00329015 | –9.8 | –10.0 |
| 1055 | UMHSN00294523 | –10.0 | –10.1 | 1106 | UMHSN00391043 | –9.9 | –10.0 |
| 1056 | UMHSN00316436 | –10.8 | –10.1 | 1107 | UMHSN00004380 | –9.6 | –10.0 |
| 1057 | UMHSN00331018 | –10.0 | –10.1 | 1108 | UMHSN00008445 | –9.7 | –10.0 |
| 1058 | UMHSN00005523 | –9.9 | –10.0 | 1109 | UMHSN00024017 | –9.9 | –10.0 |
| 1059 | UMHSN00008365 | –10.0 | –10.0 | 1110 | UMHSN00052477 | –9.9 | –10.0 |
| 1060 | UMHSN00009043 | –10.0 | –10.0 | 1111 | UMHSN00053312 | –10.4 | –10.0 |
| 1061 | UMHSN00009868 | –9.9 | –10.0 | 1112 | UMHSN00055936 | –9.8 | –10.0 |
| 1062 | UMHSN00009886 | –10.0 | –10.0 | 1113 | UMHSN00057425 | –9.7 | –10.0 |
| 1063 | UMHSN00010016 | –10.1 | –10.0 | 1114 | UMHSN00059455 | –9.8 | –10.0 |
| 1064 | UMHSN00012726 | –10.0 | –10.0 | 1115 | UMHSN00076482 | –10.0 | –10.0 |
| 1065 | UMHSN00013927 | –10.1 | –10.0 | 1116 | UMHSN00078994 | –10.0 | –10.0 |
| 1066 | UMHSN00015680 | –10.0 | –10.0 | 1117 | UMHSN00086483 | –9.9 | –10.0 |
| 1067 | UMHSN00018633 | –9.9 | –10.0 | 1118 | UMHSN00087954 | –9.9 | –10.0 |
| 1068 | UMHSN00054202 | –9.9 | –10.0 | 1119 | UMHSN00090541 | –10.0 | –10.0 |
| 1069 | UMHSN00055572 | –9.6 | –10.0 | 1120 | UMHSN00091598 | –9.4 | –10.0 |
| 1070 | UMHSN00055593 | –9.9 | –10.0 | 1121 | UMHSN00094046 | –9.8 | –10.0 |
| 1071 | UMHSN00057948 | –10.0 | –10.0 | 1122 | UMHSN00094120 | –9.4 | –10.0 |

| No. | SuperNatural II Code | Docking Score (kcal/mol) | | No. | SuperNatural II Code | Docking Score (kcal/mol) | |
| --- | --- | --- | --- | --- | --- | --- | --- |
|  |  | Std.^b^ | Mod.^c^ |  |  | Std.^b^ | Mod.^c^ |
| 1123 | UMHSN00096168 | –9.7 | –10.0 | 1174 | UMHSN00150813 | –9.6 | –10.0 |
| 1124 | UMHSN00096772 | –9.8 | –10.0 | 1175 | UMHSN00153976 | –9.9 | –10.0 |
| 1125 | UMHSN00134010 | –9.4 | –10.0 | 1176 | UMHSN00232258 | –10.0 | –10.0 |
| 1126 | UMHSN00144251 | –10.0 | –10.0 | 1177 | UMHSN00005431 | –9.5 | –10.0 |
| 1127 | UMHSN00239961 | –10.0 | –10.0 | 1178 | UMHSN00008783 | –10.0 | –10.0 |
| 1128 | UMHSN00243328 | –10.0 | –10.0 | 1179 | UMHSN00009925 | –9.7 | –10.0 |
| 1129 | UMHSN00262837 | –9.8 | –10.0 | 1180 | UMHSN00010621 | –10.6 | –10.0 |
| 1130 | UMHSN00268526 | –9.6 | –10.0 | 1181 | UMHSN00011841 | –10.0 | –10.0 |
| 1131 | UMHSN00309229 | –9.5 | –10.0 | 1182 | UMHSN00014789 | –9.9 | –10.0 |
| 1132 | UMHSN00315035 | –10.1 | –10.0 | 1183 | UMHSN00018155 | –9.7 | –10.0 |
| 1133 | UMHSN00327199 | –9.5 | –10.0 | 1184 | UMHSN00026117 | –9.8 | –10.0 |
| 1134 | UMHSN00008450 | –9.9 | –10.0 | 1185 | UMHSN00031457 | –9.9 | –10.0 |
| 1135 | UMHSN00009035 | –9.8 | –10.0 | 1186 | UMHSN00048518 | –9.9 | –10.0 |
| 1136 | UMHSN00009191 | –10.0 | –10.0 | 1187 | UMHSN00050338 | –9.5 | –10.0 |
| 1137 | UMHSN00009813 | –10.0 | –10.0 | 1188 | UMHSN00058235 | –9.8 | –10.0 |
| 1138 | UMHSN00010805 | –9.9 | –10.0 | 1189 | UMHSN00062208 | –9.8 | –10.0 |
| 1139 | UMHSN00013933 | –10.0 | –10.0 | 1190 | UMHSN00063585 | –9.4 | –10.0 |
| 1140 | UMHSN00031197 | –9.7 | –10.0 | 1191 | UMHSN00078864 | –10.3 | –10.0 |
| 1141 | UMHSN00049947 | –9.9 | –10.0 | 1192 | UMHSN00081786 | –9.9 | –10.0 |
| 1142 | UMHSN00058228 | –10.1 | –10.0 | 1193 | UMHSN00096698 | –9.7 | –10.0 |
| 1143 | UMHSN00066431 | –9.6 | –10.0 | 1194 | UMHSN00098137 | –9.9 | –10.0 |
| 1144 | UMHSN00072447 | –9.5 | –10.0 | 1195 | UMHSN00144364 | –10.0 | –10.0 |
| 1145 | UMHSN00079344 | –10.3 | –10.0 | 1196 | UMHSN00152308 | –9.9 | –10.0 |
| 1146 | UMHSN00080684 | –9.8 | –10.0 | 1197 | UMHSN00152482 | –10.0 | –10.0 |
| 1147 | UMHSN00080911 | –10.3 | –10.0 | 1198 | UMHSN00241022 | –9.8 | –10.0 |
| 1148 | UMHSN00081532 | –9.9 | –10.0 | 1199 | UMHSN00275361 | –10.0 | –10.0 |
| 1149 | UMHSN00081776 | –9.7 | –10.0 | 1200 | UMHSN00333529 | –9.8 | –10.0 |
| 1150 | UMHSN00097409 | –10.0 | –10.0 | 1201 | UMHSN00350391 | –9.9 | –10.0 |
| 1151 | UMHSN00113993 | –9.5 | –10.0 | 1202 | UMHSN00371122 | –9.7 | –10.0 |
| 1152 | UMHSN00134164 | –10.0 | –10.0 | 1203 | UMHSN00396211 | –10.0 | –10.0 |
| 1153 | UMHSN00141900 | –9.9 | –10.0 | 1204 | UMHSN00004631 | –10.0 | –10.0 |
| 1154 | UMHSN00147101 | –10.2 | –10.0 | 1205 | UMHSN00006349 | –10.0 | –10.0 |
| 1155 | UMHSN00230178 | –10.0 | –10.0 | 1206 | UMHSN00008438 | –9.7 | –10.0 |
| 1156 | UMHSN00256309 | –9.6 | –10.0 | 1207 | UMHSN00008660 | –9.9 | –10.0 |
| 1157 | UMHSN00268359 | –9.8 | –10.0 | 1208 | UMHSN00014934 | –10.0 | –10.0 |
| 1158 | UMHSN00273445 | –10.1 | –10.0 | 1209 | UMHSN00015630 | –10.0 | –10.0 |
| 1159 | UMHSN00294466 | –9.9 | –10.0 | 1210 | UMHSN00021807 | –9.9 | –10.0 |
| 1160 | UMHSN00324248 | –10.0 | –10.0 | 1211 | UMHSN00048501 | –9.9 | –10.0 |
| 1161 | UMHSN00005545 | –10.0 | –10.0 | 1212 | UMHSN00054985 | –10.1 | –10.0 |
| 1162 | UMHSN00007944 | –9.9 | –10.0 | 1213 | UMHSN00061362 | –9.9 | –10.0 |
| 1163 | UMHSN00008144 | –9.4 | –10.0 | 1214 | UMHSN00076847 | –10.0 | –10.0 |
| 1164 | UMHSN00008503 | –9.9 | –10.0 | 1215 | UMHSN00082294 | –9.4 | –10.0 |
| 1165 | UMHSN00021470 | –9.9 | –10.0 | 1216 | UMHSN00111789 | –10.0 | –10.0 |
| 1166 | UMHSN00060615 | –9.4 | –10.0 | 1217 | UMHSN00146006 | –9.6 | –10.0 |
| 1167 | UMHSN00070807 | –9.6 | –10.0 | 1218 | UMHSN00156962 | –10.0 | –10.0 |
| 1168 | UMHSN00090381 | –9.9 | –10.0 | 1219 | UMHSN00165057 | –10.0 | –10.0 |
| 1169 | UMHSN00093835 | –10.0 | –10.0 | 1220 | UMHSN00236184 | –9.9 | –10.0 |
| 1170 | UMHSN00104732 | –10.0 | –10.0 | 1221 | UMHSN00273058 | –10.0 | –10.0 |
| 1171 | UMHSN00126504 | –9.9 | –10.0 | 1222 | UMHSN00274430 | –10.0 | –10.0 |
| 1172 | UMHSN00135524 | –9.8 | –10.0 | 1223 | UMHSN00301710 | –9.6 | –10.0 |
| 1173 | UMHSN00150716 | –9.9 | –10.0 | 1224 | UMHSN00313504 | –9.4 | –10.0 |

| No. | SuperNatural II Code | Docking Score (kcal/mol) | | No. | SuperNatural II Code | Docking Score (kcal/mol) | |
| --- | --- | --- | --- | --- | --- | --- | --- |
|  |  | Std.^b^ | Mod.^c^ |  |  | Std.^b^ | Mod.^c^ |
| 1225 | UMHSN00338314 | –10.0 | –10.0 | 1276 | UMHSN00313127 | –9.6 | –10.0 |
| 1226 | UMHSN00380797 | –10.0 | –10.0 | 1277 | UMHSN00367024 | –9.9 | –10.0 |
| 1227 | UMHSN00386514 | –9.5 | –10.0 | 1278 | UMHSN00374978 | –9.7 | –10.0 |
| 1228 | UMHSN00427087 | –9.9 | –10.0 | 1279 | UMHSN00394080 | –9.9 | –10.0 |
| 1229 | UMHSN00008570 | –9.9 | –10.0 | 1280 | UMHSN00005386 | –9.8 | –10.0 |
| 1230 | UMHSN00009818 | –10.0 | –10.0 | 1281 | UMHSN00007677 | –9.9 | –10.0 |
| 1231 | UMHSN00012124 | –10.0 | –10.0 | 1282 | UMHSN00008620 | –9.9 | –10.0 |
| 1232 | UMHSN00015958 | –10.1 | –10.0 | 1283 | UMHSN00010004 | –9.8 | –10.0 |
| 1233 | UMHSN00052836 | –9.4 | –10.0 | 1284 | UMHSN00013926 | –9.9 | –10.0 |
| 1234 | UMHSN00058442 | –9.9 | –10.0 | 1285 | UMHSN00015170 | –9.9 | –10.0 |
| 1235 | UMHSN00070962 | –9.9 | –10.0 | 1286 | UMHSN00016040 | –9.9 | –10.0 |
| 1236 | UMHSN00078936 | –10.0 | –10.0 | 1287 | UMHSN00018150 | –9.4 | –10.0 |
| 1237 | UMHSN00080886 | –9.6 | –10.0 | 1288 | UMHSN00021814 | –9.9 | –10.0 |
| 1238 | UMHSN00085522 | –10.0 | –10.0 | 1289 | UMHSN00053177 | –9.5 | –10.0 |
| 1239 | UMHSN00090415 | –9.7 | –10.0 | 1290 | UMHSN00078679 | –9.5 | –10.0 |
| 1240 | UMHSN00115467 | –9.8 | –10.0 | 1291 | UMHSN00080596 | –9.7 | –10.0 |
| 1241 | UMHSN00122556 | –10.0 | –10.0 | 1292 | UMHSN00091032 | –9.8 | –10.0 |
| 1242 | UMHSN00135616 | –10.0 | –10.0 | 1293 | UMHSN00104692 | –9.4 | –10.0 |
| 1243 | UMHSN00151799 | –9.9 | –10.0 | 1294 | UMHSN00151745 | –9.8 | –10.0 |
| 1244 | UMHSN00248056 | –10.0 | –10.0 | 1295 | UMHSN00270324 | –9.8 | –10.0 |
| 1245 | UMHSN00309892 | –9.9 | –10.0 | 1296 | UMHSN00272349 | –9.7 | –10.0 |
| 1246 | UMHSN00337998 | –9.9 | –10.0 | 1297 | UMHSN00274892 | –9.9 | –10.0 |
| 1247 | UMHSN00004662 | –9.9 | –10.0 | 1298 | UMHSN00299303 | –9.8 | –10.0 |
| 1248 | UMHSN00005515 | –9.9 | –10.0 | 1299 | UMHSN00335013 | –9.9 | –10.0 |
| 1249 | UMHSN00008369 | –10.0 | –10.0 | 1300 | UMHSN00356342 | –9.9 | –10.0 |
| 1250 | UMHSN00008393 | –9.9 | –10.0 | 1301 | UMHSN00427424 | –9.8 | –10.0 |
| 1251 | UMHSN00009731 | –9.9 | –10.0 | 1302 | UMHSN00004386 | –9.9 | –9.9 |
| 1252 | UMHSN00009977 | –9.8 | –10.0 | 1303 | UMHSN00008344 | –9.9 | –9.9 |
| 1253 | UMHSN00014342 | –9.7 | –10.0 | 1304 | UMHSN00009223 | –9.7 | –9.9 |
| 1254 | UMHSN00014854 | –10.0 | –10.0 | 1305 | UMHSN00009985 | –10.7 | –9.9 |
| 1255 | UMHSN00014905 | –9.9 | –10.0 | 1306 | UMHSN00011961 | –9.9 | –9.9 |
| 1256 | UMHSN00019721 | –9.8 | –10.0 | 1307 | UMHSN00012120 | –9.9 | –9.9 |
| 1257 | UMHSN00026115 | –9.7 | –10.0 | 1308 | UMHSN00014844 | –9.9 | –9.9 |
| 1258 | UMHSN00031096 | –9.9 | –10.0 | 1309 | UMHSN00040747 | –9.9 | –9.9 |
| 1259 | UMHSN00053908 | –9.9 | –10.0 | 1310 | UMHSN00062044 | –9.7 | –9.9 |
| 1260 | UMHSN00056945 | –10.0 | –10.0 | 1311 | UMHSN00081925 | –10.2 | –9.9 |
| 1261 | UMHSN00080522 | –9.8 | –10.0 | 1312 | UMHSN00082958 | –9.9 | –9.9 |
| 1262 | UMHSN00082458 | –10.0 | –10.0 | 1313 | UMHSN00084092 | –10.2 | –9.9 |
| 1263 | UMHSN00082676 | –10.2 | –10.0 | 1314 | UMHSN00089854 | –9.4 | –9.9 |
| 1264 | UMHSN00097199 | –9.4 | –10.0 | 1315 | UMHSN00090408 | –9.8 | –9.9 |
| 1265 | UMHSN00106547 | –9.6 | –10.0 | 1316 | UMHSN00091539 | –9.7 | –9.9 |
| 1266 | UMHSN00108460 | –10.0 | –10.0 | 1317 | UMHSN00104736 | –9.9 | –9.9 |
| 1267 | UMHSN00123829 | –10.0 | –10.0 | 1318 | UMHSN00108782 | –9.5 | –9.9 |
| 1268 | UMHSN00144283 | –9.9 | –10.0 | 1319 | UMHSN00231548 | –9.9 | –9.9 |
| 1269 | UMHSN00152399 | –9.7 | –10.0 | 1320 | UMHSN00276400 | –9.9 | –9.9 |
| 1270 | UMHSN00161020 | –9.4 | –10.0 | 1321 | UMHSN00287802 | –9.9 | –9.9 |
| 1271 | UMHSN00245339 | –9.5 | –10.0 | 1322 | UMHSN00402259 | –9.9 | –9.9 |
| 1272 | UMHSN00260706 | –9.7 | –10.0 | 1323 | UMHSN00004109 | –9.9 | –9.9 |
| 1273 | UMHSN00261068 | –9.7 | –10.0 | 1324 | UMHSN00007001 | –9.9 | –9.9 |
| 1274 | UMHSN00287366 | –10.0 | –10.0 | 1325 | UMHSN00008372 | –9.9 | –9.9 |
| 1275 | UMHSN00299810 | –9.9 | –10.0 | 1326 | UMHSN00008388 | –9.9 | –9.9 |

| No. | SuperNatural II Code | Docking Score (kcal/mol) | | No. | SuperNatural II Code | Docking Score (kcal/mol) | |
| --- | --- | --- | --- | --- | --- | --- | --- |
|  |  | Std.^b^ | Mod.^c^ |  |  | Std.^b^ | Mod.^c^ |
| 1327 | UMHSN00008391 | –9.9 | –9.9 | 1378 | UMHSN00226060 | –9.9 | –9.9 |
| 1328 | UMHSN00009178 | –10.0 | –9.9 | 1379 | UMHSN00262216 | –9.4 | –9.9 |
| 1329 | UMHSN00011769 | –9.9 | –9.9 | 1380 | UMHSN00269520 | –9.8 | –9.9 |
| 1330 | UMHSN00014561 | –9.9 | –9.9 | 1381 | UMHSN00312395 | –9.9 | –9.9 |
| 1331 | UMHSN00014808 | –9.9 | –9.9 | 1382 | UMHSN00317908 | –9.9 | –9.9 |
| 1332 | UMHSN00014826 | –9.9 | –9.9 | 1383 | UMHSN00319228 | –9.9 | –9.9 |
| 1333 | UMHSN00017327 | –9.6 | –9.9 | 1384 | UMHSN00319551 | –9.9 | –9.9 |
| 1334 | UMHSN00019611 | –9.8 | –9.9 | 1385 | UMHSN00342551 | –9.9 | –9.9 |
| 1335 | UMHSN00021867 | –9.8 | –9.9 | 1386 | UMHSN00351501 | –9.9 | –9.9 |
| 1336 | UMHSN00022183 | –9.8 | –9.9 | 1387 | UMHSN00388989 | –9.6 | –9.9 |
| 1337 | UMHSN00026940 | –9.7 | –9.9 | 1388 | UMHSN00008299 | –9.7 | –9.9 |
| 1338 | UMHSN00031410 | –9.8 | –9.9 | 1389 | UMHSN00009047 | –9.8 | –9.9 |
| 1339 | UMHSN00041081 | –9.9 | –9.9 | 1390 | UMHSN00009706 | –9.8 | –9.9 |
| 1340 | UMHSN00053034 | –9.7 | –9.9 | 1391 | UMHSN00014788 | –9.7 | –9.9 |
| 1341 | UMHSN00055196 | –9.7 | –9.9 | 1392 | UMHSN00014839 | –9.9 | –9.9 |
| 1342 | UMHSN00089933 | –9.9 | –9.9 | 1393 | UMHSN00021894 | –9.8 | –9.9 |
| 1343 | UMHSN00098944 | –9.8 | –9.9 | 1394 | UMHSN00022280 | –9.8 | –9.9 |
| 1344 | UMHSN00104662 | –9.4 | –9.9 | 1395 | UMHSN00026341 | –9.9 | –9.9 |
| 1345 | UMHSN00129447 | –9.9 | –9.9 | 1396 | UMHSN00031168 | –9.8 | –9.9 |
| 1346 | UMHSN00133618 | –9.5 | –9.9 | 1397 | UMHSN00035976 | –9.7 | –9.9 |
| 1347 | UMHSN00236870 | –9.6 | –9.9 | 1398 | UMHSN00048075 | –9.6 | –9.9 |
| 1348 | UMHSN00242820 | –9.7 | –9.9 | 1399 | UMHSN00073855 | –9.8 | –9.9 |
| 1349 | UMHSN00269625 | –9.4 | –9.9 | 1400 | UMHSN00076675 | –9.8 | –9.9 |
| 1350 | UMHSN00295336 | –9.4 | –9.9 | 1401 | UMHSN00080766 | –9.9 | –9.9 |
| 1351 | UMHSN00295749 | –9.9 | –9.9 | 1402 | UMHSN00091391 | –9.9 | –9.9 |
| 1352 | UMHSN00004665 | –9.8 | –9.9 | 1403 | UMHSN00104539 | –9.9 | –9.9 |
| 1353 | UMHSN00008304 | –9.9 | –9.9 | 1404 | UMHSN00105066 | –9.8 | –9.9 |
| 1354 | UMHSN00008394 | –9.9 | –9.9 | 1405 | UMHSN00115626 | –9.9 | –9.9 |
| 1355 | UMHSN00008762 | –9.9 | –9.9 | 1406 | UMHSN00115954 | –10.3 | –9.9 |
| 1356 | UMHSN00012115 | –9.9 | –9.9 | 1407 | UMHSN00133769 | –9.5 | –9.9 |
| 1357 | UMHSN00013391 | –9.8 | –9.9 | 1408 | UMHSN00134891 | –9.8 | –9.9 |
| 1358 | UMHSN00013990 | –9.4 | –9.9 | 1409 | UMHSN00135614 | –9.4 | –9.9 |
| 1359 | UMHSN00017266 | –9.8 | –9.9 | 1410 | UMHSN00148367 | –9.8 | –9.9 |
| 1360 | UMHSN00022177 | –9.9 | –9.9 | 1411 | UMHSN00150781 | –9.9 | –9.9 |
| 1361 | UMHSN00022222 | –9.6 | –9.9 | 1412 | UMHSN00151432 | –9.7 | –9.9 |
| 1362 | UMHSN00057605 | –9.9 | –9.9 | 1413 | UMHSN00253281 | –9.9 | –9.9 |
| 1363 | UMHSN00059103 | –9.6 | –9.9 | 1414 | UMHSN00273678 | –9.9 | –9.9 |
| 1364 | UMHSN00069762 | –9.9 | –9.9 | 1415 | UMHSN00284627 | –9.8 | –9.9 |
| 1365 | UMHSN00078824 | –10.0 | –9.9 | 1416 | UMHSN00291772 | –9.9 | –9.9 |
| 1366 | UMHSN00083728 | –9.4 | –9.9 | 1417 | UMHSN00312058 | –9.9 | –9.9 |
| 1367 | UMHSN00085017 | –9.9 | –9.9 | 1418 | UMHSN00317161 | –9.9 | –9.9 |
| 1368 | UMHSN00087839 | –9.7 | –9.9 | 1419 | UMHSN00319591 | –9.9 | –9.9 |
| 1369 | UMHSN00090125 | –9.9 | –9.9 | 1420 | UMHSN00343884 | –9.5 | –9.9 |
| 1370 | UMHSN00091021 | –9.8 | –9.9 | 1421 | UMHSN00354531 | –9.9 | –9.9 |
| 1371 | UMHSN00114088 | –9.9 | –9.9 | 1422 | UMHSN00004348 | –9.9 | –9.9 |
| 1372 | UMHSN00115989 | –9.9 | –9.9 | 1423 | UMHSN00005417 | –9.9 | –9.9 |
| 1373 | UMHSN00145766 | –9.8 | –9.9 | 1424 | UMHSN00008463 | –9.9 | –9.9 |
| 1374 | UMHSN00150746 | –9.9 | –9.9 | 1425 | UMHSN00008613 | –9.8 | –9.9 |
| 1375 | UMHSN00152555 | –9.8 | –9.9 | 1426 | UMHSN00010423 | –9.6 | –9.9 |
| 1376 | UMHSN00153883 | –9.8 | –9.9 | 1427 | UMHSN00010438 | –9.9 | –9.9 |
| 1377 | UMHSN00224407 | –9.9 | –9.9 | 1428 | UMHSN00012268 | –9.5 | –9.9 |

| No. | SuperNatural II Code | Docking Score (kcal/mol) | | No. | SuperNatural II Code | Docking Score (kcal/mol) | |
| --- | --- | --- | --- | --- | --- | --- | --- |
|  |  | Std.^b^ | Mod.^c^ |  |  | Std.^b^ | Mod.^c^ |
| 1429 | UMHSN00013042 | –9.8 | –9.9 | 1480 | UMHSN00292914 | –9.7 | –9.9 |
| 1430 | UMHSN00014559 | –9.9 | –9.9 | 1481 | UMHSN00332807 | –9.4 | –9.9 |
| 1431 | UMHSN00016031 | –9.9 | –9.9 | 1482 | UMHSN00381985 | –9.8 | –9.9 |
| 1432 | UMHSN00026343 | –9.9 | –9.9 | 1483 | UMHSN00387349 | –9.9 | –9.9 |
| 1433 | UMHSN00053054 | –9.8 | –9.9 | 1484 | UMHSN00004176 | –9.9 | –9.9 |
| 1434 | UMHSN00054476 | –9.8 | –9.9 | 1485 | UMHSN00010953 | –9.6 | –9.9 |
| 1435 | UMHSN00057070 | –9.7 | –9.9 | 1486 | UMHSN00027893 | –9.7 | –9.9 |
| 1436 | UMHSN00060641 | –9.7 | –9.9 | 1487 | UMHSN00052971 | –9.5 | –9.9 |
| 1437 | UMHSN00073840 | –9.8 | –9.9 | 1488 | UMHSN00068344 | –9.8 | –9.9 |
| 1438 | UMHSN00078051 | –9.6 | –9.9 | 1489 | UMHSN00081565 | –9.9 | –9.9 |
| 1439 | UMHSN00080679 | –9.8 | –9.9 | 1490 | UMHSN00084669 | –9.9 | –9.9 |
| 1440 | UMHSN00086432 | –9.8 | –9.9 | 1491 | UMHSN00086792 | –9.4 | –9.9 |
| 1441 | UMHSN00095903 | –9.8 | –9.9 | 1492 | UMHSN00103976 | –9.8 | –9.9 |
| 1442 | UMHSN00096299 | –9.9 | –9.9 | 1493 | UMHSN00129153 | –9.7 | –9.9 |
| 1443 | UMHSN00123522 | –9.9 | –9.9 | 1494 | UMHSN00133093 | –9.8 | –9.9 |
| 1444 | UMHSN00127157 | –9.7 | –9.9 | 1495 | UMHSN00143556 | –9.8 | –9.9 |
| 1445 | UMHSN00150672 | –9.9 | –9.9 | 1496 | UMHSN00143668 | –9.8 | –9.9 |
| 1446 | UMHSN00153576 | –9.9 | –9.9 | 1497 | UMHSN00150641 | –9.8 | –9.9 |
| 1447 | UMHSN00153855 | –9.8 | –9.9 | 1498 | UMHSN00150645 | –9.9 | –9.9 |
| 1448 | UMHSN00167058 | –9.9 | –9.9 | 1499 | UMHSN00170110 | –9.9 | –9.9 |
| 1449 | UMHSN00230623 | –9.9 | –9.9 | 1500 | UMHSN00236109 | –9.9 | –9.9 |
| 1450 | UMHSN00239187 | –9.8 | –9.9 | 1501 | UMHSN00244455 | –9.9 | –9.9 |
| 1451 | UMHSN00261500 | –9.4 | –9.9 | 1502 | UMHSN00282083 | –10.0 | –9.9 |
| 1452 | UMHSN00267205 | –9.9 | –9.9 | 1503 | UMHSN00339766 | –9.9 | –9.9 |
| 1453 | UMHSN00271602 | –9.7 | –9.9 | 1504 | UMHSN00004091 | –9.8 | –9.9 |
| 1454 | UMHSN00318144 | –9.4 | –9.9 | 1505 | UMHSN00008323 | –9.8 | –9.9 |
| 1455 | UMHSN00005509 | –9.5 | –9.9 | 1506 | UMHSN00008708 | –9.8 | –9.9 |
| 1456 | UMHSN00007657 | –9.9 | –9.9 | 1507 | UMHSN00010216 | –9.8 | –9.9 |
| 1457 | UMHSN00008363 | –9.9 | –9.9 | 1508 | UMHSN00014558 | –9.8 | –9.9 |
| 1458 | UMHSN00008491 | –9.4 | –9.9 | 1509 | UMHSN00017372 | –9.7 | –9.9 |
| 1459 | UMHSN00009041 | –9.9 | –9.9 | 1510 | UMHSN00017753 | –9.9 | –9.9 |
| 1460 | UMHSN00012172 | –9.4 | –9.9 | 1511 | UMHSN00056767 | –9.9 | –9.9 |
| 1461 | UMHSN00012288 | –9.9 | –9.9 | 1512 | UMHSN00058292 | –9.8 | –9.9 |
| 1462 | UMHSN00013120 | –9.6 | –9.9 | 1513 | UMHSN00064563 | –9.9 | –9.9 |
| 1463 | UMHSN00023741 | –9.8 | –9.9 | 1514 | UMHSN00072853 | –9.9 | –9.9 |
| 1464 | UMHSN00026279 | –9.9 | –9.9 | 1515 | UMHSN00079724 | –9.7 | –9.9 |
| 1465 | UMHSN00042477 | –9.9 | –9.9 | 1516 | UMHSN00080961 | –9.5 | –9.9 |
| 1466 | UMHSN00049415 | –9.9 | –9.9 | 1517 | UMHSN00096579 | –9.5 | –9.9 |
| 1467 | UMHSN00049741 | –9.8 | –9.9 | 1518 | UMHSN00112644 | –10.1 | –9.9 |
| 1468 | UMHSN00080565 | –9.6 | –9.9 | 1519 | UMHSN00116011 | –9.9 | –9.9 |
| 1469 | UMHSN00088351 | –9.6 | –9.9 | 1520 | UMHSN00128981 | –9.8 | –9.9 |
| 1470 | UMHSN00091402 | –10.0 | –9.9 | 1521 | UMHSN00141100 | –9.8 | –9.9 |
| 1471 | UMHSN00113278 | –9.9 | –9.9 | 1522 | UMHSN00246528 | –9.9 | –9.9 |
| 1472 | UMHSN00115885 | –9.7 | –9.9 | 1523 | UMHSN00250096 | –9.9 | –9.9 |
| 1473 | UMHSN00139910 | –9.9 | –9.9 | 1524 | UMHSN00305994 | –9.5 | –9.9 |
| 1474 | UMHSN00159326 | –9.9 | –9.9 | 1525 | UMHSN00387387 | –9.6 | –9.9 |
| 1475 | UMHSN00168945 | –9.9 | –9.9 | 1526 | UMHSN00427275 | –9.5 | –9.9 |
| 1476 | UMHSN00168979 | –9.7 | –9.9 | 1527 | UMHSN00008395 | –9.8 | –9.9 |
| 1477 | UMHSN00242052 | –9.8 | –9.9 | 1528 | UMHSN00011734 | –9.9 | –9.9 |
| 1478 | UMHSN00270642 | –9.9 | –9.9 | 1529 | UMHSN00011767 | –9.8 | –9.9 |
| 1479 | UMHSN00285606 | –9.7 | –9.9 | 1530 | UMHSN00012469 | –9.7 | –9.9 |

| No. | SuperNatural II Code | Docking Score (kcal/mol) | | No. | SuperNatural II Code | Docking Score (kcal/mol) | |
| --- | --- | --- | --- | --- | --- | --- | --- |
|  |  | Std.^b^ | Mod.^c^ |  |  | Std.^b^ | Mod.^c^ |
| 1531 | UMHSN00013330 | –9.9 | –9.9 | 1582 | UMHSN00251031 | –9.8 | –9.9 |
| 1532 | UMHSN00014562 | –9.8 | –9.9 | 1583 | UMHSN00268084 | –9.9 | –9.9 |
| 1533 | UMHSN00014903 | –9.8 | –9.9 | 1584 | UMHSN00278275 | –9.8 | –9.9 |
| 1534 | UMHSN00015296 | –9.5 | –9.9 | 1585 | UMHSN00326608 | –9.6 | –9.9 |
| 1535 | UMHSN00017276 | –9.4 | –9.9 | 1586 | UMHSN00376987 | –9.8 | –9.9 |
| 1536 | UMHSN00022671 | –9.8 | –9.9 | 1587 | UMHSN00384975 | –9.6 | –9.9 |
| 1537 | UMHSN00026465 | –9.8 | –9.9 | 1588 | UMHSN00004111 | –9.8 | –9.8 |
| 1538 | UMHSN00055604 | –9.8 | –9.9 | 1589 | UMHSN00008360 | –9.8 | –9.8 |
| 1539 | UMHSN00071421 | –10.0 | –9.9 | 1590 | UMHSN00008856 | –9.7 | –9.8 |
| 1540 | UMHSN00076390 | –9.9 | –9.9 | 1591 | UMHSN00009164 | –9.8 | –9.8 |
| 1541 | UMHSN00078054 | –9.8 | –9.9 | 1592 | UMHSN00009167 | –9.8 | –9.8 |
| 1542 | UMHSN00084344 | –9.8 | –9.9 | 1593 | UMHSN00010840 | –9.6 | –9.8 |
| 1543 | UMHSN00096780 | –9.7 | –9.9 | 1594 | UMHSN00013922 | –9.8 | –9.8 |
| 1544 | UMHSN00103691 | –9.8 | –9.9 | 1595 | UMHSN00014166 | –9.6 | –9.8 |
| 1545 | UMHSN00139903 | –9.9 | –9.9 | 1596 | UMHSN00015171 | –9.8 | –9.8 |
| 1546 | UMHSN00152403 | –9.7 | –9.9 | 1597 | UMHSN00015803 | –9.8 | –9.8 |
| 1547 | UMHSN00161176 | –9.4 | –9.9 | 1598 | UMHSN00017980 | –9.9 | –9.8 |
| 1548 | UMHSN00255566 | –9.7 | –9.9 | 1599 | UMHSN00057964 | –9.8 | –9.8 |
| 1549 | UMHSN00303336 | –9.9 | –9.9 | 1600 | UMHSN00061943 | –9.6 | –9.8 |
| 1550 | UMHSN00326311 | –9.6 | –9.9 | 1601 | UMHSN00066325 | –9.8 | –9.8 |
| 1551 | UMHSN00327781 | –9.8 | –9.9 | 1602 | UMHSN00072992 | –9.6 | –9.8 |
| 1552 | UMHSN00358389 | –9.9 | –9.9 | 1603 | UMHSN00079502 | –9.8 | –9.8 |
| 1553 | UMHSN00367855 | –9.9 | –9.9 | 1604 | UMHSN00080614 | –9.7 | –9.8 |
| 1554 | UMHSN00384195 | –9.8 | –9.9 | 1605 | UMHSN00082778 | –9.9 | –9.8 |
| 1555 | UMHSN00004125 | –9.8 | –9.9 | 1606 | UMHSN00083735 | –9.8 | –9.8 |
| 1556 | UMHSN00007062 | –9.9 | –9.9 | 1607 | UMHSN00086619 | –9.7 | –9.8 |
| 1557 | UMHSN00009146 | –9.7 | –9.9 | 1608 | UMHSN00087715 | –9.7 | –9.8 |
| 1558 | UMHSN00009837 | –9.8 | –9.9 | 1609 | UMHSN00089310 | –9.7 | –9.8 |
| 1559 | UMHSN00011702 | –9.6 | –9.9 | 1610 | UMHSN00089327 | –9.8 | –9.8 |
| 1560 | UMHSN00025976 | –9.6 | –9.9 | 1611 | UMHSN00090380 | –9.8 | –9.8 |
| 1561 | UMHSN00048808 | –9.7 | –9.9 | 1612 | UMHSN00096868 | –9.8 | –9.8 |
| 1562 | UMHSN00058380 | –9.7 | –9.9 | 1613 | UMHSN00103400 | –9.9 | –9.8 |
| 1563 | UMHSN00058513 | –9.5 | –9.9 | 1614 | UMHSN00177070 | –10.2 | –9.8 |
| 1564 | UMHSN00059337 | –9.8 | –9.9 | 1615 | UMHSN00213874 | –9.8 | –9.8 |
| 1565 | UMHSN00063093 | –9.8 | –9.9 | 1616 | UMHSN00289707 | –9.6 | –9.8 |
| 1566 | UMHSN00071577 | –9.8 | –9.9 | 1617 | UMHSN00298751 | –9.8 | –9.8 |
| 1567 | UMHSN00076913 | –9.8 | –9.9 | 1618 | UMHSN00351323 | –9.8 | –9.8 |
| 1568 | UMHSN00078836 | –10.0 | –9.9 | 1619 | UMHSN00351586 | –9.8 | –9.8 |
| 1569 | UMHSN00081690 | –9.6 | –9.9 | 1620 | UMHSN00352224 | –9.6 | –9.8 |
| 1570 | UMHSN00089722 | –9.5 | –9.9 | 1621 | UMHSN00004336 | –9.8 | –9.8 |
| 1571 | UMHSN00108219 | –9.9 | –9.9 | 1622 | UMHSN00005615 | –9.8 | –9.8 |
| 1572 | UMHSN00119190 | –9.8 | –9.9 | 1623 | UMHSN00013973 | –9.6 | –9.8 |
| 1573 | UMHSN00122783 | –9.8 | –9.9 | 1624 | UMHSN00014599 | –9.8 | –9.8 |
| 1574 | UMHSN00150719 | –9.8 | –9.9 | 1625 | UMHSN00017323 | –10.1 | –9.8 |
| 1575 | UMHSN00152355 | –9.9 | –9.9 | 1626 | UMHSN00019660 | –9.8 | –9.8 |
| 1576 | UMHSN00152446 | –9.8 | –9.9 | 1627 | UMHSN00048529 | –9.8 | –9.8 |
| 1577 | UMHSN00153291 | –9.9 | –9.9 | 1628 | UMHSN00049424 | –9.4 | –9.8 |
| 1578 | UMHSN00215155 | –9.8 | –9.9 | 1629 | UMHSN00054152 | –9.5 | –9.8 |
| 1579 | UMHSN00226265 | –9.9 | –9.9 | 1630 | UMHSN00055945 | –9.7 | –9.8 |
| 1580 | UMHSN00232686 | –9.6 | –9.9 | 1631 | UMHSN00080694 | –10.4 | –9.8 |
| 1581 | UMHSN00249156 | –9.8 | –9.9 | 1632 | UMHSN00090826 | –9.7 | –9.8 |

| No. | SuperNatural II Code | Docking Score (kcal/mol) | | No. | SuperNatural II Code | Docking Score (kcal/mol) | |
| --- | --- | --- | --- | --- | --- | --- | --- |
|  |  | Std.^b^ | Mod.^c^ |  |  | Std.^b^ | Mod.^c^ |
| 1633 | UMHSN00094518 | –9.6 | –9.8 | 1684 | UMHSN00139907 | –9.8 | –9.8 |
| 1634 | UMHSN00103939 | –9.8 | –9.8 | 1685 | UMHSN00152384 | –9.7 | –9.8 |
| 1635 | UMHSN00110243 | –9.8 | –9.8 | 1686 | UMHSN00152566 | –9.8 | –9.8 |
| 1636 | UMHSN00142216 | –9.9 | –9.8 | 1687 | UMHSN00169141 | –9.7 | –9.8 |
| 1637 | UMHSN00150008 | –9.4 | –9.8 | 1688 | UMHSN00169272 | –9.7 | –9.8 |
| 1638 | UMHSN00153093 | –9.8 | –9.8 | 1689 | UMHSN00234116 | –9.7 | –9.8 |
| 1639 | UMHSN00238757 | –9.8 | –9.8 | 1690 | UMHSN00276543 | –9.8 | –9.8 |
| 1640 | UMHSN00278844 | –9.9 | –9.8 | 1691 | UMHSN00294995 | –10.8 | –9.8 |
| 1641 | UMHSN00401144 | –9.6 | –9.8 | 1692 | UMHSN00004180 | –9.5 | –9.8 |
| 1642 | UMHSN00428753 | –9.8 | –9.8 | 1693 | UMHSN00009165 | –9.4 | –9.8 |
| 1643 | UMHSN00008451 | –9.8 | –9.8 | 1694 | UMHSN00009183 | –9.6 | –9.8 |
| 1644 | UMHSN00008626 | –9.8 | –9.8 | 1695 | UMHSN00009801 | –9.8 | –9.8 |
| 1645 | UMHSN00009833 | –9.8 | –9.8 | 1696 | UMHSN00013287 | –9.8 | –9.8 |
| 1646 | UMHSN00010130 | –9.7 | –9.8 | 1697 | UMHSN00014851 | –9.9 | –9.8 |
| 1647 | UMHSN00014859 | –9.8 | –9.8 | 1698 | UMHSN00049451 | –9.5 | –9.8 |
| 1648 | UMHSN00015173 | –9.5 | –9.8 | 1699 | UMHSN00057732 | –9.7 | –9.8 |
| 1649 | UMHSN00016638 | –9.8 | –9.8 | 1700 | UMHSN00063439 | –9.8 | –9.8 |
| 1650 | UMHSN00053080 | –9.7 | –9.8 | 1701 | UMHSN00063489 | –9.4 | –9.8 |
| 1651 | UMHSN00058669 | –9.7 | –9.8 | 1702 | UMHSN00070605 | –9.4 | –9.8 |
| 1652 | UMHSN00076800 | –9.8 | –9.8 | 1703 | UMHSN00082689 | –9.8 | –9.8 |
| 1653 | UMHSN00083004 | –9.4 | –9.8 | 1704 | UMHSN00082707 | –9.8 | –9.8 |
| 1654 | UMHSN00084874 | –9.8 | –9.8 | 1705 | UMHSN00089363 | –9.8 | –9.8 |
| 1655 | UMHSN00085591 | –9.8 | –9.8 | 1706 | UMHSN00094081 | –9.7 | –9.8 |
| 1656 | UMHSN00091515 | –9.8 | –9.8 | 1707 | UMHSN00100219 | –9.7 | –9.8 |
| 1657 | UMHSN00149928 | –9.6 | –9.8 | 1708 | UMHSN00111971 | –9.7 | –9.8 |
| 1658 | UMHSN00150491 | –9.7 | –9.8 | 1709 | UMHSN00123512 | –9.7 | –9.8 |
| 1659 | UMHSN00240841 | –9.8 | –9.8 | 1710 | UMHSN00145267 | –9.8 | –9.8 |
| 1660 | UMHSN00241466 | –9.8 | –9.8 | 1711 | UMHSN00149947 | –9.7 | –9.8 |
| 1661 | UMHSN00246468 | –9.8 | –9.8 | 1712 | UMHSN00166660 | –9.8 | –9.8 |
| 1662 | UMHSN00274825 | –9.9 | –9.8 | 1713 | UMHSN00246318 | –9.7 | –9.8 |
| 1663 | UMHSN00288846 | –9.8 | –9.8 | 1714 | UMHSN00303804 | –9.8 | –9.8 |
| 1664 | UMHSN00291811 | –9.7 | –9.8 | 1715 | UMHSN00304629 | –9.4 | –9.8 |
| 1665 | UMHSN00370893 | –9.8 | –9.8 | 1716 | UMHSN00327191 | –9.8 | –9.8 |
| 1666 | UMHSN00384573 | –9.5 | –9.8 | 1717 | UMHSN00343839 | –9.8 | –9.8 |
| 1667 | UMHSN00403822 | –9.8 | –9.8 | 1718 | UMHSN00391888 | –9.4 | –9.8 |
| 1668 | UMHSN00008704 | –9.7 | –9.8 | 1719 | UMHSN00007125 | –9.8 | –9.8 |
| 1669 | UMHSN00009869 | –9.8 | –9.8 | 1720 | UMHSN00007696 | –9.5 | –9.8 |
| 1670 | UMHSN00011732 | –9.5 | –9.8 | 1721 | UMHSN00008357 | –9.8 | –9.8 |
| 1671 | UMHSN00011828 | –9.8 | –9.8 | 1722 | UMHSN00008362 | –9.8 | –9.8 |
| 1672 | UMHSN00014749 | –9.7 | –9.8 | 1723 | UMHSN00015789 | –9.6 | –9.8 |
| 1673 | UMHSN00019279 | –9.6 | –9.8 | 1724 | UMHSN00016029 | –9.8 | –9.8 |
| 1674 | UMHSN00024148 | –9.6 | –9.8 | 1725 | UMHSN00026293 | –10.0 | –9.8 |
| 1675 | UMHSN00026276 | –9.8 | –9.8 | 1726 | UMHSN00041532 | –9.6 | –9.8 |
| 1676 | UMHSN00057240 | –10.3 | –9.8 | 1727 | UMHSN00068077 | –9.7 | –9.8 |
| 1677 | UMHSN00080661 | –9.6 | –9.8 | 1728 | UMHSN00076938 | –9.7 | –9.8 |
| 1678 | UMHSN00081637 | –9.5 | –9.8 | 1729 | UMHSN00081857 | –9.8 | –9.8 |
| 1679 | UMHSN00081934 | –9.6 | –9.8 | 1730 | UMHSN00082663 | –9.6 | –9.8 |
| 1680 | UMHSN00092351 | –9.6 | –9.8 | 1731 | UMHSN00082806 | –9.4 | –9.8 |
| 1681 | UMHSN00105949 | –9.8 | –9.8 | 1732 | UMHSN00091599 | –9.7 | –9.8 |
| 1682 | UMHSN00123692 | –9.8 | –9.8 | 1733 | UMHSN00104012 | –9.8 | –9.8 |
| 1683 | UMHSN00128512 | –9.8 | –9.8 | 1734 | UMHSN00104349 | –9.7 | –9.8 |

| No. | SuperNatural II Code | Docking Score (kcal/mol) | | No. | SuperNatural II Code | Docking Score (kcal/mol) | |
| --- | --- | --- | --- | --- | --- | --- | --- |
|  |  | Std.^b^ | Mod.^c^ |  |  | Std.^b^ | Mod.^c^ |
| 1735 | UMHSN00104371 | –9.7 | –9.8 | 1786 | UMHSN00076964 | –9.7 | –9.8 |
| 1736 | UMHSN00132527 | –9.7 | –9.8 | 1787 | UMHSN00078407 | –9.4 | –9.8 |
| 1737 | UMHSN00153254 | –9.6 | –9.8 | 1788 | UMHSN00080337 | –9.4 | –9.8 |
| 1738 | UMHSN00170560 | –9.7 | –9.8 | 1789 | UMHSN00081181 | –9.7 | –9.8 |
| 1739 | UMHSN00254646 | –9.8 | –9.8 | 1790 | UMHSN00081675 | –9.8 | –9.8 |
| 1740 | UMHSN00308408 | –9.8 | –9.8 | 1791 | UMHSN00082712 | –9.7 | –9.8 |
| 1741 | UMHSN00326986 | –9.5 | –9.8 | 1792 | UMHSN00085005 | –9.7 | –9.8 |
| 1742 | UMHSN00348976 | –9.8 | –9.8 | 1793 | UMHSN00096599 | –9.7 | –9.8 |
| 1743 | UMHSN00356016 | –9.8 | –9.8 | 1794 | UMHSN00100695 | –9.5 | –9.8 |
| 1744 | UMHSN00356918 | –9.8 | –9.8 | 1795 | UMHSN00102411 | –9.8 | –9.8 |
| 1745 | UMHSN00427213 | –9.7 | –9.8 | 1796 | UMHSN00112410 | –10.0 | –9.8 |
| 1746 | UMHSN00008295 | –10.5 | –9.8 | 1797 | UMHSN00112699 | –9.6 | –9.8 |
| 1747 | UMHSN00008786 | –9.7 | –9.8 | 1798 | UMHSN00127141 | –9.6 | –9.8 |
| 1748 | UMHSN00009741 | –9.4 | –9.8 | 1799 | UMHSN00134085 | –9.6 | –9.8 |
| 1749 | UMHSN00010818 | –9.8 | –9.8 | 1800 | UMHSN00134173 | –9.4 | –9.8 |
| 1750 | UMHSN00010819 | –9.7 | –9.8 | 1801 | UMHSN00144521 | –9.7 | –9.8 |
| 1751 | UMHSN00011019 | –9.7 | –9.8 | 1802 | UMHSN00146274 | –9.4 | –9.8 |
| 1752 | UMHSN00012300 | –9.8 | –9.8 | 1803 | UMHSN00150642 | –9.5 | –9.8 |
| 1753 | UMHSN00014791 | –9.5 | –9.8 | 1804 | UMHSN00150712 | –9.4 | –9.8 |
| 1754 | UMHSN00014825 | –9.8 | –9.8 | 1805 | UMHSN00151766 | –9.8 | –9.8 |
| 1755 | UMHSN00021599 | –9.7 | –9.8 | 1806 | UMHSN00153869 | –9.6 | –9.8 |
| 1756 | UMHSN00048213 | –9.7 | –9.8 | 1807 | UMHSN00254925 | –9.6 | –9.8 |
| 1757 | UMHSN00062309 | –9.7 | –9.8 | 1808 | UMHSN00259284 | –9.8 | –9.8 |
| 1758 | UMHSN00076915 | –9.4 | –9.8 | 1809 | UMHSN00271795 | –9.8 | –9.8 |
| 1759 | UMHSN00079077 | –9.6 | –9.8 | 1810 | UMHSN00276692 | –9.6 | –9.8 |
| 1760 | UMHSN00100819 | –9.8 | –9.8 | 1811 | UMHSN00331975 | –9.8 | –9.8 |
| 1761 | UMHSN00104739 | –9.8 | –9.8 | 1812 | UMHSN00360523 | –9.8 | –9.8 |
| 1762 | UMHSN00106030 | –9.8 | –9.8 | 1813 | UMHSN00361631 | –9.5 | –9.8 |
| 1763 | UMHSN00115515 | –9.8 | –9.8 | 1814 | UMHSN00392641 | –9.7 | –9.8 |
| 1764 | UMHSN00148726 | –9.7 | –9.8 | 1815 | UMHSN00005123 | –9.6 | –9.8 |
| 1765 | UMHSN00148974 | –10.1 | –9.8 | 1816 | UMHSN00005500 | –9.8 | –9.8 |
| 1766 | UMHSN00150322 | –9.5 | –9.8 | 1817 | UMHSN00008981 | –9.4 | –9.8 |
| 1767 | UMHSN00163740 | –9.4 | –9.8 | 1818 | UMHSN00009055 | –9.7 | –9.8 |
| 1768 | UMHSN00240936 | –9.7 | –9.8 | 1819 | UMHSN00010585 | –9.6 | –9.8 |
| 1769 | UMHSN00332855 | –9.8 | –9.8 | 1820 | UMHSN00011883 | –9.6 | –9.8 |
| 1770 | UMHSN00337587 | –9.8 | –9.8 | 1821 | UMHSN00014897 | –9.7 | –9.8 |
| 1771 | UMHSN00345985 | –9.8 | –9.8 | 1822 | UMHSN00014998 | –10.3 | –9.8 |
| 1772 | UMHSN00427139 | –9.7 | –9.8 | 1823 | UMHSN00021874 | –9.8 | –9.8 |
| 1773 | UMHSN00004338 | –9.8 | –9.8 | 1824 | UMHSN00025056 | –9.5 | –9.8 |
| 1774 | UMHSN00008298 | –9.4 | –9.8 | 1825 | UMHSN00031385 | –9.6 | –9.8 |
| 1775 | UMHSN00008993 | –9.4 | –9.8 | 1826 | UMHSN00048264 | –9.7 | –9.8 |
| 1776 | UMHSN00012108 | –9.6 | –9.8 | 1827 | UMHSN00055961 | –9.5 | –9.8 |
| 1777 | UMHSN00014753 | –9.7 | –9.8 | 1828 | UMHSN00073901 | –9.7 | –9.8 |
| 1778 | UMHSN00015139 | –9.8 | –9.8 | 1829 | UMHSN00081634 | –9.7 | –9.8 |
| 1779 | UMHSN00017057 | –9.7 | –9.8 | 1830 | UMHSN00083515 | –9.8 | –9.8 |
| 1780 | UMHSN00019711 | –9.5 | –9.8 | 1831 | UMHSN00085382 | –9.8 | –9.8 |
| 1781 | UMHSN00053452 | –9.6 | –9.8 | 1832 | UMHSN00088365 | –9.6 | –9.8 |
| 1782 | UMHSN00053638 | –9.5 | –9.8 | 1833 | UMHSN00104016 | –9.7 | –9.8 |
| 1783 | UMHSN00055000 | –9.5 | –9.8 | 1834 | UMHSN00115184 | –9.7 | –9.8 |
| 1784 | UMHSN00062194 | –9.5 | –9.8 | 1835 | UMHSN00142398 | –9.7 | –9.8 |
| 1785 | UMHSN00062749 | –9.7 | –9.8 | 1836 | UMHSN00151420 | –9.7 | –9.8 |

| No. | SuperNatural II Code | Docking Score (kcal/mol) | | No. | SuperNatural II Code | Docking Score (kcal/mol) | |
| --- | --- | --- | --- | --- | --- | --- | --- |
|  |  | Std.^b^ | Mod.^c^ |  |  | Std.^b^ | Mod.^c^ |
| 1837 | UMHSN00153870 | –9.6 | –9.8 | 1888 | UMHSN00078569 | –10.3 | –9.7 |
| 1838 | UMHSN00162747 | –9.6 | –9.8 | 1889 | UMHSN00087596 | –9.5 | –9.7 |
| 1839 | UMHSN00168547 | –9.7 | –9.8 | 1890 | UMHSN00088478 | –10.9 | –9.7 |
| 1840 | UMHSN00262742 | –9.8 | –9.8 | 1891 | UMHSN00091432 | –9.4 | –9.7 |
| 1841 | UMHSN00381049 | –9.5 | –9.8 | 1892 | UMHSN00106557 | –9.6 | –9.7 |
| 1842 | UMHSN00403760 | –9.7 | –9.8 | 1893 | UMHSN00107489 | –9.7 | –9.7 |
| 1843 | UMHSN00003607 | –9.8 | –9.8 | 1894 | UMHSN00112370 | –9.8 | –9.7 |
| 1844 | UMHSN00008859 | –9.6 | –9.8 | 1895 | UMHSN00115753 | –9.7 | –9.7 |
| 1845 | UMHSN00013334 | –9.7 | –9.8 | 1896 | UMHSN00130002 | –9.7 | –9.7 |
| 1846 | UMHSN00014890 | –9.7 | –9.8 | 1897 | UMHSN00130899 | –9.7 | –9.7 |
| 1847 | UMHSN00019656 | –9.7 | –9.8 | 1898 | UMHSN00149935 | –9.5 | –9.7 |
| 1848 | UMHSN00038957 | –9.6 | –9.8 | 1899 | UMHSN00153294 | –9.7 | –9.7 |
| 1849 | UMHSN00048250 | –9.6 | –9.8 | 1900 | UMHSN00243675 | –9.4 | –9.7 |
| 1850 | UMHSN00055038 | –9.7 | –9.8 | 1901 | UMHSN00270060 | –9.7 | –9.7 |
| 1851 | UMHSN00055948 | –9.6 | –9.8 | 1902 | UMHSN00301961 | –9.7 | –9.7 |
| 1852 | UMHSN00056170 | –9.6 | –9.8 | 1903 | UMHSN00309387 | –9.7 | –9.7 |
| 1853 | UMHSN00084332 | –9.7 | –9.8 | 1904 | UMHSN00314141 | –9.7 | –9.7 |
| 1854 | UMHSN00091290 | –9.6 | –9.8 | 1905 | UMHSN00316585 | –9.7 | –9.7 |
| 1855 | UMHSN00091604 | –9.7 | –9.8 | 1906 | UMHSN00359879 | –9.7 | –9.7 |
| 1856 | UMHSN00104319 | –9.7 | –9.8 | 1907 | UMHSN00370510 | –9.4 | –9.7 |
| 1857 | UMHSN00110118 | –9.4 | –9.8 | 1908 | UMHSN00401535 | –9.7 | –9.7 |
| 1858 | UMHSN00150744 | –9.6 | –9.8 | 1909 | UMHSN00005429 | –9.7 | –9.7 |
| 1859 | UMHSN00153843 | –9.4 | –9.8 | 1910 | UMHSN00013965 | –9.7 | –9.7 |
| 1860 | UMHSN00154025 | –10.1 | –9.8 | 1911 | UMHSN00014850 | –9.7 | –9.7 |
| 1861 | UMHSN00230942 | –9.7 | –9.8 | 1912 | UMHSN00015679 | –9.7 | –9.7 |
| 1862 | UMHSN00248159 | –9.7 | –9.8 | 1913 | UMHSN00054794 | –9.7 | –9.7 |
| 1863 | UMHSN00250291 | –9.6 | –9.8 | 1914 | UMHSN00055261 | –9.6 | –9.7 |
| 1864 | UMHSN00256652 | –9.8 | –9.8 | 1915 | UMHSN00056472 | –9.5 | –9.7 |
| 1865 | UMHSN00261119 | –9.8 | –9.8 | 1916 | UMHSN00071362 | –9.7 | –9.7 |
| 1866 | UMHSN00309722 | –9.6 | –9.8 | 1917 | UMHSN00076365 | –9.6 | –9.7 |
| 1867 | UMHSN00356669 | –10.2 | –9.8 | 1918 | UMHSN00081774 | –9.5 | –9.7 |
| 1868 | UMHSN00370247 | –9.7 | –9.8 | 1919 | UMHSN00081831 | –9.7 | –9.7 |
| 1869 | UMHSN00004089 | –9.7 | –9.7 | 1920 | UMHSN00091525 | –9.8 | –9.7 |
| 1870 | UMHSN00005411 | –9.7 | –9.7 | 1921 | UMHSN00093994 | –9.6 | –9.7 |
| 1871 | UMHSN00008455 | –9.7 | –9.7 | 1922 | UMHSN00095838 | –9.7 | –9.7 |
| 1872 | UMHSN00010121 | –9.7 | –9.7 | 1923 | UMHSN00096506 | –9.7 | –9.7 |
| 1873 | UMHSN00011016 | –9.7 | –9.7 | 1924 | UMHSN00108243 | –9.7 | –9.7 |
| 1874 | UMHSN00012233 | –9.5 | –9.7 | 1925 | UMHSN00112071 | –9.5 | –9.7 |
| 1875 | UMHSN00013428 | –9.5 | –9.7 | 1926 | UMHSN00134066 | –9.7 | –9.7 |
| 1876 | UMHSN00014778 | –9.7 | –9.7 | 1927 | UMHSN00150871 | –9.5 | –9.7 |
| 1877 | UMHSN00015137 | –9.7 | –9.7 | 1928 | UMHSN00153486 | –9.7 | –9.7 |
| 1878 | UMHSN00016043 | –9.7 | –9.7 | 1929 | UMHSN00153828 | –9.4 | –9.7 |
| 1879 | UMHSN00019681 | –9.7 | –9.7 | 1930 | UMHSN00160733 | –9.5 | –9.7 |
| 1880 | UMHSN00021830 | –9.7 | –9.7 | 1931 | UMHSN00170567 | –9.6 | –9.7 |
| 1881 | UMHSN00026457 | –9.4 | –9.7 | 1932 | UMHSN00220497 | –9.7 | –9.7 |
| 1882 | UMHSN00035694 | –9.7 | –9.7 | 1933 | UMHSN00230200 | –9.5 | –9.7 |
| 1883 | UMHSN00050505 | –9.4 | –9.7 | 1934 | UMHSN00268506 | –9.5 | –9.7 |
| 1884 | UMHSN00057824 | –9.7 | –9.7 | 1935 | UMHSN00269865 | –9.7 | –9.7 |
| 1885 | UMHSN00058553 | –9.7 | –9.7 | 1936 | UMHSN00270286 | –9.7 | –9.7 |
| 1886 | UMHSN00059047 | –9.7 | –9.7 | 1937 | UMHSN00276896 | –9.8 | –9.7 |
| 1887 | UMHSN00078224 | –9.8 | –9.7 | 1938 | UMHSN00361466 | –9.7 | –9.7 |

| No. | SuperNatural II Code | Docking Score (kcal/mol) | | No. | SuperNatural II Code | Docking Score (kcal/mol) | |
| --- | --- | --- | --- | --- | --- | --- | --- |
|  |  | Std.^b^ | Mod.^c^ |  |  | Std.^b^ | Mod.^c^ |
| 1939 | UMHSN00373116 | –10.0 | –9.7 | 1990 | UMHSN00074739 | –9.7 | –9.7 |
| 1940 | UMHSN00388676 | –9.7 | –9.7 | 1991 | UMHSN00088701 | –9.4 | –9.7 |
| 1941 | UMHSN00391775 | –9.7 | –9.7 | 1992 | UMHSN00089683 | –9.6 | –9.7 |
| 1942 | UMHSN00394937 | –9.4 | –9.7 | 1993 | UMHSN00090513 | –9.4 | –9.7 |
| 1943 | UMHSN00004648 | –9.6 | –9.7 | 1994 | UMHSN00090642 | –9.4 | –9.7 |
| 1944 | UMHSN00005496 | –9.6 | –9.7 | 1995 | UMHSN00096756 | –9.7 | –9.7 |
| 1945 | UMHSN00007699 | –9.4 | –9.7 | 1996 | UMHSN00097807 | –9.5 | –9.7 |
| 1946 | UMHSN00008152 | –9.9 | –9.7 | 1997 | UMHSN00103936 | –9.4 | –9.7 |
| 1947 | UMHSN00009171 | –9.7 | –9.7 | 1998 | UMHSN00113403 | –10.1 | –9.7 |
| 1948 | UMHSN00009179 | –9.7 | –9.7 | 1999 | UMHSN00125495 | –9.4 | –9.7 |
| 1949 | UMHSN00009728 | –9.6 | –9.7 | 2000 | UMHSN00132091 | –9.7 | –9.7 |
| 1950 | UMHSN00012259 | –9.5 | –9.7 | 2001 | UMHSN00132334 | –9.7 | –9.7 |
| 1951 | UMHSN00017745 | –9.7 | –9.7 | 2002 | UMHSN00152361 | –9.7 | –9.7 |
| 1952 | UMHSN00020432 | –9.5 | –9.7 | 2003 | UMHSN00152447 | –9.7 | –9.7 |
| 1953 | UMHSN00026123 | –9.8 | –9.7 | 2004 | UMHSN00225959 | –9.5 | –9.7 |
| 1954 | UMHSN00039360 | –9.7 | –9.7 | 2005 | UMHSN00313669 | –10.8 | –9.7 |
| 1955 | UMHSN00076771 | –9.7 | –9.7 | 2006 | UMHSN00387131 | –9.7 | –9.7 |
| 1956 | UMHSN00082406 | –9.7 | –9.7 | 2007 | UMHSN00391382 | –9.7 | –9.7 |
| 1957 | UMHSN00083162 | –9.9 | –9.7 | 2008 | UMHSN00004342 | –9.6 | –9.7 |
| 1958 | UMHSN00084666 | –10.1 | –9.7 | 2009 | UMHSN00005412 | –9.7 | –9.7 |
| 1959 | UMHSN00089718 | –9.5 | –9.7 | 2010 | UMHSN00007006 | –9.7 | –9.7 |
| 1960 | UMHSN00090571 | –9.6 | –9.7 | 2011 | UMHSN00008147 | –9.7 | –9.7 |
| 1961 | UMHSN00094134 | –9.5 | –9.7 | 2012 | UMHSN00008389 | –9.7 | –9.7 |
| 1962 | UMHSN00097671 | –9.6 | –9.7 | 2013 | UMHSN00009727 | –9.7 | –9.7 |
| 1963 | UMHSN00104785 | –9.7 | –9.7 | 2014 | UMHSN00010809 | –9.7 | –9.7 |
| 1964 | UMHSN00108355 | –9.7 | –9.7 | 2015 | UMHSN00014560 | –9.5 | –9.7 |
| 1965 | UMHSN00132332 | –9.5 | –9.7 | 2016 | UMHSN00016560 | –9.5 | –9.7 |
| 1966 | UMHSN00133645 | –9.7 | –9.7 | 2017 | UMHSN00021561 | –9.4 | –9.7 |
| 1967 | UMHSN00151800 | –9.5 | –9.7 | 2018 | UMHSN00026297 | –9.7 | –9.7 |
| 1968 | UMHSN00215125 | –9.7 | –9.7 | 2019 | UMHSN00026347 | –9.7 | –9.7 |
| 1969 | UMHSN00242770 | –9.7 | –9.7 | 2020 | UMHSN00050479 | –9.4 | –9.7 |
| 1970 | UMHSN00282795 | –9.7 | –9.7 | 2021 | UMHSN00063292 | –9.7 | –9.7 |
| 1971 | UMHSN00295317 | –9.7 | –9.7 | 2022 | UMHSN00081547 | –9.7 | –9.7 |
| 1972 | UMHSN00386933 | –9.7 | –9.7 | 2023 | UMHSN00089721 | –9.7 | –9.7 |
| 1973 | UMHSN00404718 | –9.7 | –9.7 | 2024 | UMHSN00094306 | –9.5 | –9.7 |
| 1974 | UMHSN00004212 | –9.7 | –9.7 | 2025 | UMHSN00105813 | –9.6 | –9.7 |
| 1975 | UMHSN00008183 | –9.6 | –9.7 | 2026 | UMHSN00106601 | –9.6 | –9.7 |
| 1976 | UMHSN00010367 | –9.6 | –9.7 | 2027 | UMHSN00112402 | –9.7 | –9.7 |
| 1977 | UMHSN00012095 | –10.0 | –9.7 | 2028 | UMHSN00112740 | –9.4 | –9.7 |
| 1978 | UMHSN00015608 | –9.6 | –9.7 | 2029 | UMHSN00115143 | –9.6 | –9.7 |
| 1979 | UMHSN00016033 | –9.7 | –9.7 | 2030 | UMHSN00119229 | –9.7 | –9.7 |
| 1980 | UMHSN00016918 | –9.5 | –9.7 | 2031 | UMHSN00128590 | –9.7 | –9.7 |
| 1981 | UMHSN00019669 | –9.6 | –9.7 | 2032 | UMHSN00152188 | –10.9 | –9.7 |
| 1982 | UMHSN00022176 | –9.6 | –9.7 | 2033 | UMHSN00153418 | –9.5 | –9.7 |
| 1983 | UMHSN00029573 | –9.7 | –9.7 | 2034 | UMHSN00161762 | –9.5 | –9.7 |
| 1984 | UMHSN00052648 | –9.4 | –9.7 | 2035 | UMHSN00237684 | –9.6 | –9.7 |
| 1985 | UMHSN00052687 | –9.5 | –9.7 | 2036 | UMHSN00246111 | –9.7 | –9.7 |
| 1986 | UMHSN00056895 | –9.6 | –9.7 | 2037 | UMHSN00268761 | –9.7 | –9.7 |
| 1987 | UMHSN00057324 | –9.6 | –9.7 | 2038 | UMHSN00295476 | –9.7 | –9.7 |
| 1988 | UMHSN00063957 | –9.7 | –9.7 | 2039 | UMHSN00335502 | –9.7 | –9.7 |
| 1989 | UMHSN00064101 | –9.7 | –9.7 | 2040 | UMHSN00376893 | –9.7 | –9.7 |

| No. | SuperNatural II Code | Docking Score (kcal/mol) | | No. | SuperNatural II Code | Docking Score (kcal/mol) | |
| --- | --- | --- | --- | --- | --- | --- | --- |
|  |  | Std.^b^ | Mod.^c^ |  |  | Std.^b^ | Mod.^c^ |
| 2041 | UMHSN00381244 | –9.6 | –9.7 | 2092 | UMHSN00103368 | –9.6 | –9.7 |
| 2042 | UMHSN00427300 | –9.5 | –9.7 | 2093 | UMHSN00104782 | –9.6 | –9.7 |
| 2043 | UMHSN00005433 | –9.7 | –9.7 | 2094 | UMHSN00108312 | –9.6 | –9.7 |
| 2044 | UMHSN00008433 | –9.6 | –9.7 | 2095 | UMHSN00120526 | –9.6 | –9.7 |
| 2045 | UMHSN00009790 | –9.7 | –9.7 | 2096 | UMHSN00146845 | –9.6 | –9.7 |
| 2046 | UMHSN00015604 | –9.5 | –9.7 | 2097 | UMHSN00149951 | –9.4 | –9.7 |
| 2047 | UMHSN00016028 | –9.7 | –9.7 | 2098 | UMHSN00151762 | –9.6 | –9.7 |
| 2048 | UMHSN00016171 | –9.6 | –9.7 | 2099 | UMHSN00152427 | –9.6 | –9.7 |
| 2049 | UMHSN00022017 | –9.5 | –9.7 | 2100 | UMHSN00227207 | –9.7 | –9.7 |
| 2050 | UMHSN00024777 | –9.6 | –9.7 | 2101 | UMHSN00234035 | –9.5 | –9.7 |
| 2051 | UMHSN00029842 | –9.5 | –9.7 | 2102 | UMHSN00247588 | –9.5 | –9.7 |
| 2052 | UMHSN00069032 | –9.7 | –9.7 | 2103 | UMHSN00254011 | –9.7 | –9.7 |
| 2053 | UMHSN00097215 | –9.7 | –9.7 | 2104 | UMHSN00266293 | –9.5 | –9.7 |
| 2054 | UMHSN00115741 | –9.7 | –9.7 | 2105 | UMHSN00289010 | –9.7 | –9.7 |
| 2055 | UMHSN00127989 | –9.7 | –9.7 | 2106 | UMHSN00289430 | –9.6 | –9.7 |
| 2056 | UMHSN00139912 | –9.7 | –9.7 | 2107 | UMHSN00316831 | –9.7 | –9.7 |
| 2057 | UMHSN00150872 | –9.7 | –9.7 | 2108 | UMHSN00322875 | –9.6 | –9.7 |
| 2058 | UMHSN00151743 | –9.7 | –9.7 | 2109 | UMHSN00339988 | –9.7 | –9.7 |
| 2059 | UMHSN00153882 | –9.5 | –9.7 | 2110 | UMHSN00005614 | –9.6 | –9.7 |
| 2060 | UMHSN00166597 | –9.5 | –9.7 | 2111 | UMHSN00007440 | –9.6 | –9.7 |
| 2061 | UMHSN00215103 | –9.7 | –9.7 | 2112 | UMHSN00008410 | –9.7 | –9.7 |
| 2062 | UMHSN00275603 | –9.6 | –9.7 | 2113 | UMHSN00008591 | –9.4 | –9.7 |
| 2063 | UMHSN00291533 | –9.5 | –9.7 | 2114 | UMHSN00008695 | –9.7 | –9.7 |
| 2064 | UMHSN00292641 | –9.6 | –9.7 | 2115 | UMHSN00009832 | –9.7 | –9.7 |
| 2065 | UMHSN00307242 | –9.6 | –9.7 | 2116 | UMHSN00011173 | –9.7 | –9.7 |
| 2066 | UMHSN00339515 | –9.7 | –9.7 | 2117 | UMHSN00015392 | –9.6 | –9.7 |
| 2067 | UMHSN00402590 | –9.7 | –9.7 | 2118 | UMHSN00017243 | –9.5 | –9.7 |
| 2068 | UMHSN00004653 | –9.5 | –9.7 | 2119 | UMHSN00021576 | –9.7 | –9.7 |
| 2069 | UMHSN00005124 | –9.5 | –9.7 | 2120 | UMHSN00034351 | –9.7 | –9.7 |
| 2070 | UMHSN00007684 | –9.7 | –9.7 | 2121 | UMHSN00057442 | –9.4 | –9.7 |
| 2071 | UMHSN00008475 | –9.7 | –9.7 | 2122 | UMHSN00059248 | –9.5 | –9.7 |
| 2072 | UMHSN00009184 | –9.6 | –9.7 | 2123 | UMHSN00077194 | –9.6 | –9.7 |
| 2073 | UMHSN00009784 | –9.6 | –9.7 | 2124 | UMHSN00079515 | –9.6 | –9.7 |
| 2074 | UMHSN00012379 | –10.8 | –9.7 | 2125 | UMHSN00094023 | –9.6 | –9.7 |
| 2075 | UMHSN00012881 | –9.6 | –9.7 | 2126 | UMHSN00096719 | –9.6 | –9.7 |
| 2076 | UMHSN00013043 | –9.6 | –9.7 | 2127 | UMHSN00104008 | –9.6 | –9.7 |
| 2077 | UMHSN00014050 | –9.4 | –9.7 | 2128 | UMHSN00111973 | –9.6 | –9.7 |
| 2078 | UMHSN00015060 | –9.7 | –9.7 | 2129 | UMHSN00133643 | –9.7 | –9.7 |
| 2079 | UMHSN00015135 | –9.6 | –9.7 | 2130 | UMHSN00135504 | –9.6 | –9.7 |
| 2080 | UMHSN00024068 | –9.6 | –9.7 | 2131 | UMHSN00151753 | –9.6 | –9.7 |
| 2081 | UMHSN00050014 | –9.5 | –9.7 | 2132 | UMHSN00153841 | –9.6 | –9.7 |
| 2082 | UMHSN00055698 | –9.4 | –9.7 | 2133 | UMHSN00160117 | –9.5 | –9.7 |
| 2083 | UMHSN00058810 | –9.7 | –9.7 | 2134 | UMHSN00250980 | –9.7 | –9.7 |
| 2084 | UMHSN00063236 | –9.6 | –9.7 | 2135 | UMHSN00298107 | –9.7 | –9.7 |
| 2085 | UMHSN00072595 | –9.7 | –9.7 | 2136 | UMHSN00309649 | –9.7 | –9.7 |
| 2086 | UMHSN00079004 | –9.6 | –9.7 | 2137 | UMHSN00321671 | –9.7 | –9.7 |
| 2087 | UMHSN00083370 | –9.7 | –9.7 | 2138 | UMHSN00329868 | –9.6 | –9.7 |
| 2088 | UMHSN00084343 | –9.6 | –9.7 | 2139 | UMHSN00005172 | –9.5 | –9.7 |
| 2089 | UMHSN00088347 | –9.6 | –9.7 | 2140 | UMHSN00006400 | –9.6 | –9.7 |
| 2090 | UMHSN00091578 | –9.5 | –9.7 | 2141 | UMHSN00008509 | –9.7 | –9.7 |
| 2091 | UMHSN00100799 | –9.7 | –9.7 | 2142 | UMHSN00008709 | –9.6 | –9.7 |

| No. | SuperNatural II Code | Docking Score (kcal/mol) | | No. | SuperNatural II Code | Docking Score (kcal/mol) | |
| --- | --- | --- | --- | --- | --- | --- | --- |
|  |  | Std.^b^ | Mod.^c^ |  |  | Std.^b^ | Mod.^c^ |
| 2143 | UMHSN00008796 | –9.4 | –9.7 | 2194 | UMHSN00078394 | –9.5 | –9.7 |
| 2144 | UMHSN00009233 | –9.7 | –9.7 | 2195 | UMHSN00079130 | –9.6 | –9.7 |
| 2145 | UMHSN00009834 | –9.6 | –9.7 | 2196 | UMHSN00080926 | –9.5 | –9.7 |
| 2146 | UMHSN00014843 | –9.6 | –9.7 | 2197 | UMHSN00082118 | –9.7 | –9.7 |
| 2147 | UMHSN00014853 | –9.6 | –9.7 | 2198 | UMHSN00094040 | –9.5 | –9.7 |
| 2148 | UMHSN00015141 | –9.6 | –9.7 | 2199 | UMHSN00094693 | –9.4 | –9.7 |
| 2149 | UMHSN00016940 | –10.6 | –9.7 | 2200 | UMHSN00102809 | –9.5 | –9.7 |
| 2150 | UMHSN00020159 | –9.6 | –9.7 | 2201 | UMHSN00103881 | –9.6 | –9.7 |
| 2151 | UMHSN00053786 | –9.9 | –9.7 | 2202 | UMHSN00106597 | –9.6 | –9.7 |
| 2152 | UMHSN00074709 | –9.6 | –9.7 | 2203 | UMHSN00111861 | –9.6 | –9.7 |
| 2153 | UMHSN00083919 | –9.6 | –9.7 | 2204 | UMHSN00115949 | –9.6 | –9.7 |
| 2154 | UMHSN00088106 | –9.7 | –9.7 | 2205 | UMHSN00133837 | –9.6 | –9.7 |
| 2155 | UMHSN00088502 | –9.4 | –9.7 | 2206 | UMHSN00144688 | –9.7 | –9.7 |
| 2156 | UMHSN00090484 | –9.5 | –9.7 | 2207 | UMHSN00150309 | –9.5 | –9.7 |
| 2157 | UMHSN00095413 | –9.6 | –9.7 | 2208 | UMHSN00153822 | –9.4 | –9.7 |
| 2158 | UMHSN00096796 | –9.6 | –9.7 | 2209 | UMHSN00160615 | –9.6 | –9.7 |
| 2159 | UMHSN00102114 | –9.7 | –9.7 | 2210 | UMHSN00168537 | –9.6 | –9.7 |
| 2160 | UMHSN00102701 | –9.7 | –9.7 | 2211 | UMHSN00169261 | –9.5 | –9.7 |
| 2161 | UMHSN00106677 | –9.7 | –9.7 | 2212 | UMHSN00170104 | –9.7 | –9.7 |
| 2162 | UMHSN00111909 | –9.5 | –9.7 | 2213 | UMHSN00238504 | –9.7 | –9.7 |
| 2163 | UMHSN00121847 | –9.5 | –9.7 | 2214 | UMHSN00286656 | –9.6 | –9.7 |
| 2164 | UMHSN00123281 | –9.6 | –9.7 | 2215 | UMHSN00292755 | –9.5 | –9.7 |
| 2165 | UMHSN00128592 | –9.7 | –9.7 | 2216 | UMHSN00314276 | –9.5 | –9.7 |
| 2166 | UMHSN00154038 | –9.6 | –9.7 | 2217 | UMHSN00357020 | –9.5 | –9.7 |
| 2167 | UMHSN00158311 | –9.6 | –9.7 | 2218 | UMHSN00372317 | –9.7 | –9.7 |
| 2168 | UMHSN00162188 | –9.5 | –9.7 | 2219 | UMHSN00387243 | –9.6 | –9.7 |
| 2169 | UMHSN00169143 | –9.6 | –9.7 | 2220 | UMHSN00389537 | –9.6 | –9.7 |
| 2170 | UMHSN00228825 | –9.7 | –9.7 | 2221 | UMHSN00005293 | –9.6 | –9.6 |
| 2171 | UMHSN00236598 | –9.6 | –9.7 | 2222 | UMHSN00007114 | –9.6 | –9.6 |
| 2172 | UMHSN00244766 | –9.7 | –9.7 | 2223 | UMHSN00007678 | –9.6 | –9.6 |
| 2173 | UMHSN00250101 | –9.5 | –9.7 | 2224 | UMHSN00010226 | –9.7 | –9.6 |
| 2174 | UMHSN00254724 | –9.4 | –9.7 | 2225 | UMHSN00010808 | –9.6 | –9.6 |
| 2175 | UMHSN00270399 | –9.6 | –9.7 | 2226 | UMHSN00011821 | –9.6 | –9.6 |
| 2176 | UMHSN00280511 | –9.6 | –9.7 | 2227 | UMHSN00014896 | –9.6 | –9.6 |
| 2177 | UMHSN00334378 | –9.7 | –9.7 | 2228 | UMHSN00016064 | –9.6 | –9.6 |
| 2178 | UMHSN00352765 | –9.6 | –9.7 | 2229 | UMHSN00016452 | –9.6 | –9.6 |
| 2179 | UMHSN00381288 | –9.7 | –9.7 | 2230 | UMHSN00017265 | –9.4 | –9.6 |
| 2180 | UMHSN00005179 | –9.6 | –9.7 | 2231 | UMHSN00023739 | –9.6 | –9.6 |
| 2181 | UMHSN00005354 | –9.5 | –9.7 | 2232 | UMHSN00024159 | –9.5 | –9.6 |
| 2182 | UMHSN00006677 | –9.6 | –9.7 | 2233 | UMHSN00031880 | –9.5 | –9.6 |
| 2183 | UMHSN00009858 | –9.4 | –9.7 | 2234 | UMHSN00031881 | –9.5 | –9.6 |
| 2184 | UMHSN00010250 | –9.6 | –9.7 | 2235 | UMHSN00053525 | –9.6 | –9.6 |
| 2185 | UMHSN00011754 | –9.5 | –9.7 | 2236 | UMHSN00057139 | –9.6 | –9.6 |
| 2186 | UMHSN00014841 | –9.4 | –9.7 | 2237 | UMHSN00057230 | –9.5 | –9.6 |
| 2187 | UMHSN00017061 | –9.6 | –9.7 | 2238 | UMHSN00057581 | –9.4 | –9.6 |
| 2188 | UMHSN00026482 | –9.6 | –9.7 | 2239 | UMHSN00061575 | –9.4 | –9.6 |
| 2189 | UMHSN00053026 | –9.6 | –9.7 | 2240 | UMHSN00067023 | –9.6 | –9.6 |
| 2190 | UMHSN00053867 | –9.4 | –9.7 | 2241 | UMHSN00076696 | –9.8 | –9.6 |
| 2191 | UMHSN00055165 | –9.5 | –9.7 | 2242 | UMHSN00080542 | –9.6 | –9.6 |
| 2192 | UMHSN00058082 | –9.5 | –9.7 | 2243 | UMHSN00090056 | –9.5 | –9.6 |
| 2193 | UMHSN00065722 | –9.7 | –9.7 | 2244 | UMHSN00112447 | –9.6 | –9.6 |

| No. | SuperNatural II Code | Docking Score (kcal/mol) | | No. | SuperNatural II Code | Docking Score (kcal/mol) | |
| --- | --- | --- | --- | --- | --- | --- | --- |
|  |  | Std.^b^ | Mod.^c^ |  |  | Std.^b^ | Mod.^c^ |
| 2245 | UMHSN00124083 | –9.8 | –9.6 | 2296 | UMHSN00067971 | –9.4 | –9.6 |
| 2246 | UMHSN00125288 | –9.4 | –9.6 | 2297 | UMHSN00069821 | –9.6 | –9.6 |
| 2247 | UMHSN00152250 | –9.6 | –9.6 | 2298 | UMHSN00070567 | –9.5 | –9.6 |
| 2248 | UMHSN00171076 | –9.6 | –9.6 | 2299 | UMHSN00071485 | –10.2 | –9.6 |
| 2249 | UMHSN00247025 | –9.5 | –9.6 | 2300 | UMHSN00074431 | –9.6 | –9.6 |
| 2250 | UMHSN00262054 | –10.0 | –9.6 | 2301 | UMHSN00078135 | –9.5 | –9.6 |
| 2251 | UMHSN00277731 | –9.5 | –9.6 | 2302 | UMHSN00078222 | –9.5 | –9.6 |
| 2252 | UMHSN00300490 | –9.5 | –9.6 | 2303 | UMHSN00081162 | –9.5 | –9.6 |
| 2253 | UMHSN00305315 | –9.6 | –9.6 | 2304 | UMHSN00091333 | –9.6 | –9.6 |
| 2254 | UMHSN00337611 | –9.6 | –9.6 | 2305 | UMHSN00091636 | –10.1 | –9.6 |
| 2255 | UMHSN00345387 | –9.6 | –9.6 | 2306 | UMHSN00104005 | –9.6 | –9.6 |
| 2256 | UMHSN00376101 | –9.6 | –9.6 | 2307 | UMHSN00112455 | –9.4 | –9.6 |
| 2257 | UMHSN00014831 | –9.6 | –9.6 | 2308 | UMHSN00128673 | –9.6 | –9.6 |
| 2258 | UMHSN00015692 | –9.6 | –9.6 | 2309 | UMHSN00151747 | –9.6 | –9.6 |
| 2259 | UMHSN00017414 | –9.7 | –9.6 | 2310 | UMHSN00151797 | –9.6 | –9.6 |
| 2260 | UMHSN00019119 | –9.5 | –9.6 | 2311 | UMHSN00153854 | –9.4 | –9.6 |
| 2261 | UMHSN00019610 | –9.6 | –9.6 | 2312 | UMHSN00160111 | –9.4 | –9.6 |
| 2262 | UMHSN00021663 | –9.4 | –9.6 | 2313 | UMHSN00162550 | –9.5 | –9.6 |
| 2263 | UMHSN00022446 | –9.5 | –9.6 | 2314 | UMHSN00169527 | –9.5 | –9.6 |
| 2264 | UMHSN00051820 | –9.6 | –9.6 | 2315 | UMHSN00246073 | –9.6 | –9.6 |
| 2265 | UMHSN00052498 | –9.8 | –9.6 | 2316 | UMHSN00311953 | –9.6 | –9.6 |
| 2266 | UMHSN00058932 | –9.6 | –9.6 | 2317 | UMHSN00316975 | –9.5 | –9.6 |
| 2267 | UMHSN00058973 | –9.5 | –9.6 | 2318 | UMHSN00326236 | –9.6 | –9.6 |
| 2268 | UMHSN00063080 | –9.5 | –9.6 | 2319 | UMHSN00326370 | –9.6 | –9.6 |
| 2269 | UMHSN00071689 | –9.4 | –9.6 | 2320 | UMHSN00365195 | –9.7 | –9.6 |
| 2270 | UMHSN00076060 | –9.6 | –9.6 | 2321 | UMHSN00390817 | –9.5 | –9.6 |
| 2271 | UMHSN00085582 | –9.6 | –9.6 | 2322 | UMHSN00004657 | –9.6 | –9.6 |
| 2272 | UMHSN00090842 | –9.6 | –9.6 | 2323 | UMHSN00008480 | –9.6 | –9.6 |
| 2273 | UMHSN00090866 | –9.6 | –9.6 | 2324 | UMHSN00008495 | –9.6 | –9.6 |
| 2274 | UMHSN00112170 | –9.7 | –9.6 | 2325 | UMHSN00008498 | –9.6 | –9.6 |
| 2275 | UMHSN00117804 | –9.6 | –9.6 | 2326 | UMHSN00008499 | –9.6 | –9.6 |
| 2276 | UMHSN00142313 | –9.6 | –9.6 | 2327 | UMHSN00009064 | –9.8 | –9.6 |
| 2277 | UMHSN00153274 | –9.6 | –9.6 | 2328 | UMHSN00011717 | –9.6 | –9.6 |
| 2278 | UMHSN00155109 | –10.4 | –9.6 | 2329 | UMHSN00015030 | –10.4 | –9.6 |
| 2279 | UMHSN00214596 | –9.6 | –9.6 | 2330 | UMHSN00017275 | –9.6 | –9.6 |
| 2280 | UMHSN00220488 | –9.6 | –9.6 | 2331 | UMHSN00021471 | –9.5 | –9.6 |
| 2281 | UMHSN00224612 | –9.6 | –9.6 | 2332 | UMHSN00048083 | –9.4 | –9.6 |
| 2282 | UMHSN00228065 | –9.5 | –9.6 | 2333 | UMHSN00048420 | –9.6 | –9.6 |
| 2283 | UMHSN00233361 | –9.6 | –9.6 | 2334 | UMHSN00058587 | –9.6 | –9.6 |
| 2284 | UMHSN00312777 | –9.4 | –9.6 | 2335 | UMHSN00061801 | –9.5 | –9.6 |
| 2285 | UMHSN00387252 | –9.6 | –9.6 | 2336 | UMHSN00066594 | –9.6 | –9.6 |
| 2286 | UMHSN00004092 | –9.6 | –9.6 | 2337 | UMHSN00077182 | –9.6 | –9.6 |
| 2287 | UMHSN00005283 | –9.6 | –9.6 | 2338 | UMHSN00090322 | –9.5 | –9.6 |
| 2288 | UMHSN00005541 | –9.4 | –9.6 | 2339 | UMHSN00103546 | –9.5 | –9.6 |
| 2289 | UMHSN00008314 | –9.6 | –9.6 | 2340 | UMHSN00111505 | –9.6 | –9.6 |
| 2290 | UMHSN00013341 | –9.5 | –9.6 | 2341 | UMHSN00126042 | –9.6 | –9.6 |
| 2291 | UMHSN00014045 | –9.6 | –9.6 | 2342 | UMHSN00128374 | –9.6 | –9.6 |
| 2292 | UMHSN00015158 | –9.6 | –9.6 | 2343 | UMHSN00136100 | –9.6 | –9.6 |
| 2293 | UMHSN00016623 | –9.5 | –9.6 | 2344 | UMHSN00150623 | –9.6 | –9.6 |
| 2294 | UMHSN00053304 | –9.4 | –9.6 | 2345 | UMHSN00151179 | –9.5 | –9.6 |
| 2295 | UMHSN00063348 | –9.5 | –9.6 | 2346 | UMHSN00168566 | –9.6 | –9.6 |

| No. | SuperNatural II Code | Docking Score (kcal/mol) | | No. | SuperNatural II Code | Docking Score (kcal/mol) | |
| --- | --- | --- | --- | --- | --- | --- | --- |
|  |  | Std.^b^ | Mod.^c^ |  |  | Std.^b^ | Mod.^c^ |
| 2347 | UMHSN00213516 | –9.5 | –9.6 | 2398 | UMHSN00376176 | –9.5 | –9.6 |
| 2348 | UMHSN00244799 | –9.6 | –9.6 | 2399 | UMHSN00004659 | –9.6 | –9.6 |
| 2349 | UMHSN00253751 | –9.6 | –9.6 | 2400 | UMHSN00008466 | –9.6 | –9.6 |
| 2350 | UMHSN00254959 | –9.6 | –9.6 | 2401 | UMHSN00009830 | –9.6 | –9.6 |
| 2351 | UMHSN00266382 | –9.6 | –9.6 | 2402 | UMHSN00013454 | –9.6 | –9.6 |
| 2352 | UMHSN00279992 | –9.4 | –9.6 | 2403 | UMHSN00014997 | –9.4 | –9.6 |
| 2353 | UMHSN00300939 | –9.5 | –9.6 | 2404 | UMHSN00048858 | –9.5 | –9.6 |
| 2354 | UMHSN00332664 | –9.5 | –9.6 | 2405 | UMHSN00048871 | –9.9 | –9.6 |
| 2355 | UMHSN00353519 | –9.6 | –9.6 | 2406 | UMHSN00052707 | –9.5 | –9.6 |
| 2356 | UMHSN00379696 | –9.6 | –9.6 | 2407 | UMHSN00055481 | –9.6 | –9.6 |
| 2357 | UMHSN00399299 | –9.6 | –9.6 | 2408 | UMHSN00059485 | –9.5 | –9.6 |
| 2358 | UMHSN00428812 | –9.6 | –9.6 | 2409 | UMHSN00067264 | –9.6 | –9.6 |
| 2359 | UMHSN00001635 | –9.6 | –9.6 | 2410 | UMHSN00076337 | –9.7 | –9.6 |
| 2360 | UMHSN00004052 | –9.6 | –9.6 | 2411 | UMHSN00079876 | –9.6 | –9.6 |
| 2361 | UMHSN00007126 | –9.6 | –9.6 | 2412 | UMHSN00080640 | –9.6 | –9.6 |
| 2362 | UMHSN00008550 | –9.5 | –9.6 | 2413 | UMHSN00081859 | –9.4 | –9.6 |
| 2363 | UMHSN00009148 | –9.6 | –9.6 | 2414 | UMHSN00081918 | –9.4 | –9.6 |
| 2364 | UMHSN00011705 | –9.6 | –9.6 | 2415 | UMHSN00082267 | –9.6 | –9.6 |
| 2365 | UMHSN00012129 | –9.6 | –9.6 | 2416 | UMHSN00094026 | –9.5 | –9.6 |
| 2366 | UMHSN00014061 | –9.8 | –9.6 | 2417 | UMHSN00097190 | –9.6 | –9.6 |
| 2367 | UMHSN00015959 | –9.6 | –9.6 | 2418 | UMHSN00097243 | –9.4 | –9.6 |
| 2368 | UMHSN00019676 | –9.5 | –9.6 | 2419 | UMHSN00099315 | –9.6 | –9.6 |
| 2369 | UMHSN00019716 | –9.6 | –9.6 | 2420 | UMHSN00112435 | –9.6 | –9.6 |
| 2370 | UMHSN00024736 | –9.4 | –9.6 | 2421 | UMHSN00162899 | –9.6 | –9.6 |
| 2371 | UMHSN00035503 | –9.6 | –9.6 | 2422 | UMHSN00166590 | –9.5 | –9.6 |
| 2372 | UMHSN00057669 | –9.6 | –9.6 | 2423 | UMHSN00169286 | –9.4 | –9.6 |
| 2373 | UMHSN00071387 | –9.6 | –9.6 | 2424 | UMHSN00277895 | –9.4 | –9.6 |
| 2374 | UMHSN00077391 | –9.4 | –9.6 | 2425 | UMHSN00290123 | –9.4 | –9.6 |
| 2375 | UMHSN00081588 | –9.5 | –9.6 | 2426 | UMHSN00298088 | –9.6 | –9.6 |
| 2376 | UMHSN00086356 | –9.4 | –9.6 | 2427 | UMHSN00308705 | –9.6 | –9.6 |
| 2377 | UMHSN00091980 | –9.5 | –9.6 | 2428 | UMHSN00316303 | –9.6 | –9.6 |
| 2378 | UMHSN00097393 | –9.4 | –9.6 | 2429 | UMHSN00317816 | –9.6 | –9.6 |
| 2379 | UMHSN00098315 | –9.4 | –9.6 | 2430 | UMHSN00426852 | –9.4 | –9.6 |
| 2380 | UMHSN00103887 | –9.4 | –9.6 | 2431 | UMHSN00008479 | –9.5 | –9.6 |
| 2381 | UMHSN00112083 | –9.5 | –9.6 | 2432 | UMHSN00011757 | –9.6 | –9.6 |
| 2382 | UMHSN00113128 | –9.5 | –9.6 | 2433 | UMHSN00015636 | –9.5 | –9.6 |
| 2383 | UMHSN00144555 | –9.6 | –9.6 | 2434 | UMHSN00017325 | –9.5 | –9.6 |
| 2384 | UMHSN00148438 | –9.7 | –9.6 | 2435 | UMHSN00020475 | –9.6 | –9.6 |
| 2385 | UMHSN00150077 | –9.6 | –9.6 | 2436 | UMHSN00049427 | –9.5 | –9.6 |
| 2386 | UMHSN00152101 | –9.5 | –9.6 | 2437 | UMHSN00065959 | –9.5 | –9.6 |
| 2387 | UMHSN00152230 | –9.5 | –9.6 | 2438 | UMHSN00073712 | –9.5 | –9.6 |
| 2388 | UMHSN00152266 | –9.6 | –9.6 | 2439 | UMHSN00078573 | –9.4 | –9.6 |
| 2389 | UMHSN00157921 | –9.6 | –9.6 | 2440 | UMHSN00081820 | –9.4 | –9.6 |
| 2390 | UMHSN00167055 | –9.6 | –9.6 | 2441 | UMHSN00085738 | –9.6 | –9.6 |
| 2391 | UMHSN00170102 | –9.5 | –9.6 | 2442 | UMHSN00096037 | –9.6 | –9.6 |
| 2392 | UMHSN00271133 | –9.5 | –9.6 | 2443 | UMHSN00101191 | –9.6 | –9.6 |
| 2393 | UMHSN00294856 | –9.4 | –9.6 | 2444 | UMHSN00103270 | –9.4 | –9.6 |
| 2394 | UMHSN00304652 | –9.6 | –9.6 | 2445 | UMHSN00103991 | –9.5 | –9.6 |
| 2395 | UMHSN00319811 | –9.6 | –9.6 | 2446 | UMHSN00104802 | –9.5 | –9.6 |
| 2396 | UMHSN00335910 | –9.6 | –9.6 | 2447 | UMHSN00139905 | –9.6 | –9.6 |
| 2397 | UMHSN00350516 | –9.6 | –9.6 | 2448 | UMHSN00153905 | –9.4 | –9.6 |

| No. | SuperNatural II Code | Docking Score (kcal/mol) | | No. | SuperNatural II Code | Docking Score (kcal/mol) | |
| --- | --- | --- | --- | --- | --- | --- | --- |
|  |  | Std.^b^ | Mod.^c^ |  |  | Std.^b^ | Mod.^c^ |
| 2449 | UMHSN00162551 | –9.5 | –9.6 | 2500 | UMHSN00150814 | –9.4 | –9.6 |
| 2450 | UMHSN00163077 | –9.5 | –9.6 | 2501 | UMHSN00152563 | –9.5 | –9.6 |
| 2451 | UMHSN00242295 | –9.6 | –9.6 | 2502 | UMHSN00153441 | –9.4 | –9.6 |
| 2452 | UMHSN00285311 | –9.5 | –9.6 | 2503 | UMHSN00154984 | –9.6 | –9.6 |
| 2453 | UMHSN00305123 | –9.6 | –9.6 | 2504 | UMHSN00155054 | –9.6 | –9.6 |
| 2454 | UMHSN00313275 | –9.6 | –9.6 | 2505 | UMHSN00169302 | –9.6 | –9.6 |
| 2455 | UMHSN00328882 | –9.6 | –9.6 | 2506 | UMHSN00169510 | –9.4 | –9.6 |
| 2456 | UMHSN00368798 | –9.5 | –9.6 | 2507 | UMHSN00215081 | –9.6 | –9.6 |
| 2457 | UMHSN00369764 | –9.6 | –9.6 | 2508 | UMHSN00229549 | –9.5 | –9.6 |
| 2458 | UMHSN00379330 | –9.4 | –9.6 | 2509 | UMHSN00233368 | –10.1 | –9.6 |
| 2459 | UMHSN00004112 | –9.5 | –9.6 | 2510 | UMHSN00262823 | –9.6 | –9.6 |
| 2460 | UMHSN00004151 | –9.5 | –9.6 | 2511 | UMHSN00267856 | –9.5 | –9.6 |
| 2461 | UMHSN00005180 | –9.4 | –9.6 | 2512 | UMHSN00364040 | –9.5 | –9.6 |
| 2462 | UMHSN00005211 | –9.4 | –9.6 | 2513 | UMHSN00380488 | –9.4 | –9.6 |
| 2463 | UMHSN00008380 | –9.6 | –9.6 | 2514 | UMHSN00004349 | –9.6 | –9.6 |
| 2464 | UMHSN00008485 | –9.4 | –9.6 | 2515 | UMHSN00005161 | –9.4 | –9.6 |
| 2465 | UMHSN00009250 | –9.6 | –9.6 | 2516 | UMHSN00008230 | –9.4 | –9.6 |
| 2466 | UMHSN00009525 | –9.6 | –9.6 | 2517 | UMHSN00008370 | –9.5 | –9.6 |
| 2467 | UMHSN00009743 | –9.5 | –9.6 | 2518 | UMHSN00008694 | –9.5 | –9.6 |
| 2468 | UMHSN00009884 | –9.4 | –9.6 | 2519 | UMHSN00010159 | –9.5 | –9.6 |
| 2469 | UMHSN00009906 | –9.4 | –9.6 | 2520 | UMHSN00011824 | –9.5 | –9.6 |
| 2470 | UMHSN00011644 | –9.5 | –9.6 | 2521 | UMHSN00014894 | –9.5 | –9.6 |
| 2471 | UMHSN00012304 | –9.5 | –9.6 | 2522 | UMHSN00015059 | –9.6 | –9.6 |
| 2472 | UMHSN00012614 | –9.6 | –9.6 | 2523 | UMHSN00015168 | –9.5 | –9.6 |
| 2473 | UMHSN00013339 | –9.5 | –9.6 | 2524 | UMHSN00015673 | –9.5 | –9.6 |
| 2474 | UMHSN00014814 | –9.5 | –9.6 | 2525 | UMHSN00016015 | –10.0 | –9.6 |
| 2475 | UMHSN00019626 | –9.9 | –9.6 | 2526 | UMHSN00024166 | –9.6 | –9.6 |
| 2476 | UMHSN00033663 | –9.5 | –9.6 | 2527 | UMHSN00053057 | –9.5 | –9.6 |
| 2477 | UMHSN00050334 | –9.4 | –9.6 | 2528 | UMHSN00059142 | –9.5 | –9.6 |
| 2478 | UMHSN00054017 | –9.7 | –9.6 | 2529 | UMHSN00063250 | –9.5 | –9.6 |
| 2479 | UMHSN00056636 | –9.5 | –9.6 | 2530 | UMHSN00080874 | –9.6 | –9.6 |
| 2480 | UMHSN00058813 | –9.4 | –9.6 | 2531 | UMHSN00083384 | –9.6 | –9.6 |
| 2481 | UMHSN00058890 | –9.6 | –9.6 | 2532 | UMHSN00084621 | –9.5 | –9.6 |
| 2482 | UMHSN00073044 | –9.6 | –9.6 | 2533 | UMHSN00090094 | –9.6 | –9.6 |
| 2483 | UMHSN00076022 | –10.5 | –9.6 | 2534 | UMHSN00095442 | –9.6 | –9.6 |
| 2484 | UMHSN00076704 | –9.5 | –9.6 | 2535 | UMHSN00096023 | –9.6 | –9.6 |
| 2485 | UMHSN00078525 | –9.4 | –9.6 | 2536 | UMHSN00104459 | –9.4 | –9.6 |
| 2486 | UMHSN00079412 | –9.4 | –9.6 | 2537 | UMHSN00151649 | –9.5 | –9.6 |
| 2487 | UMHSN00084579 | –9.5 | –9.6 | 2538 | UMHSN00154633 | –9.4 | –9.6 |
| 2488 | UMHSN00084679 | –9.5 | –9.6 | 2539 | UMHSN00161320 | –9.6 | –9.6 |
| 2489 | UMHSN00097364 | –9.5 | –9.6 | 2540 | UMHSN00220489 | –9.5 | –9.6 |
| 2490 | UMHSN00098899 | –9.5 | –9.6 | 2541 | UMHSN00226960 | –9.5 | –9.6 |
| 2491 | UMHSN00100775 | –9.5 | –9.6 | 2542 | UMHSN00229167 | –9.6 | –9.6 |
| 2492 | UMHSN00103392 | –9.7 | –9.6 | 2543 | UMHSN00242091 | –9.5 | –9.6 |
| 2493 | UMHSN00106199 | –9.4 | –9.6 | 2544 | UMHSN00259236 | –9.4 | –9.6 |
| 2494 | UMHSN00115237 | –9.6 | –9.6 | 2545 | UMHSN00272463 | –9.5 | –9.6 |
| 2495 | UMHSN00115523 | –9.6 | –9.6 | 2546 | UMHSN00295277 | –9.6 | –9.6 |
| 2496 | UMHSN00121440 | –9.6 | –9.6 | 2547 | UMHSN00338563 | –9.6 | –9.6 |
| 2497 | UMHSN00123877 | –9.6 | –9.6 | 2548 | UMHSN00350747 | –9.5 | –9.6 |
| 2498 | UMHSN00142312 | –9.6 | –9.6 | 2549 | UMHSN00359498 | –9.6 | –9.6 |
| 2499 | UMHSN00150795 | –9.5 | –9.6 | 2550 | UMHSN00376623 | –9.5 | –9.6 |

| No. | SuperNatural II Code | Docking Score (kcal/mol) | | No. | SuperNatural II Code | Docking Score (kcal/mol) | |
| --- | --- | --- | --- | --- | --- | --- | --- |
|  |  | Std.^b^ | Mod.^c^ |  |  | Std.^b^ | Mod.^c^ |
| 2551 | UMHSN00427308 | –9.4 | –9.6 | 2602 | UMHSN00095986 | –9.4 | –9.5 |
| 2552 | UMHSN00005306 | –10.0 | –9.6 | 2603 | UMHSN00106719 | –9.5 | –9.5 |
| 2553 | UMHSN00007099 | –10.2 | –9.6 | 2604 | UMHSN00119439 | –9.5 | –9.5 |
| 2554 | UMHSN00008379 | –9.5 | –9.6 | 2605 | UMHSN00123840 | –9.5 | –9.5 |
| 2555 | UMHSN00008562 | –9.5 | –9.6 | 2606 | UMHSN00124081 | –9.5 | –9.5 |
| 2556 | UMHSN00009707 | –9.6 | –9.6 | 2607 | UMHSN00142267 | –9.4 | –9.5 |
| 2557 | UMHSN00010320 | –9.5 | –9.6 | 2608 | UMHSN00145675 | –9.5 | –9.5 |
| 2558 | UMHSN00014913 | –9.5 | –9.6 | 2609 | UMHSN00150647 | –9.5 | –9.5 |
| 2559 | UMHSN00016074 | –9.6 | –9.6 | 2610 | UMHSN00153241 | –9.5 | –9.5 |
| 2560 | UMHSN00021565 | –9.6 | –9.6 | 2611 | UMHSN00154170 | –9.4 | –9.5 |
| 2561 | UMHSN00033161 | –9.5 | –9.6 | 2612 | UMHSN00166163 | –9.4 | –9.5 |
| 2562 | UMHSN00052969 | –9.8 | –9.6 | 2613 | UMHSN00166808 | –9.5 | –9.5 |
| 2563 | UMHSN00054733 | –9.5 | –9.6 | 2614 | UMHSN00224110 | –9.4 | –9.5 |
| 2564 | UMHSN00055452 | –9.6 | –9.6 | 2615 | UMHSN00232855 | –9.5 | –9.5 |
| 2565 | UMHSN00057604 | –9.5 | –9.6 | 2616 | UMHSN00236951 | –9.4 | –9.5 |
| 2566 | UMHSN00063703 | –9.5 | –9.6 | 2617 | UMHSN00259189 | –9.5 | –9.5 |
| 2567 | UMHSN00071051 | –9.4 | –9.6 | 2618 | UMHSN00262021 | –9.5 | –9.5 |
| 2568 | UMHSN00079602 | –9.5 | –9.6 | 2619 | UMHSN00272178 | –9.5 | –9.5 |
| 2569 | UMHSN00080570 | –9.6 | –9.6 | 2620 | UMHSN00290072 | –9.5 | –9.5 |
| 2570 | UMHSN00081075 | –9.7 | –9.6 | 2621 | UMHSN00329485 | –9.5 | –9.5 |
| 2571 | UMHSN00096630 | –9.5 | –9.6 | 2622 | UMHSN00335389 | –9.5 | –9.5 |
| 2572 | UMHSN00097203 | –9.5 | –9.6 | 2623 | UMHSN00354961 | –9.5 | –9.5 |
| 2573 | UMHSN00125292 | –9.5 | –9.6 | 2624 | UMHSN00364510 | –9.5 | –9.5 |
| 2574 | UMHSN00135505 | –9.4 | –9.6 | 2625 | UMHSN00383074 | –9.5 | –9.5 |
| 2575 | UMHSN00150188 | –9.4 | –9.6 | 2626 | UMHSN00389010 | –9.5 | –9.5 |
| 2576 | UMHSN00150646 | –9.5 | –9.6 | 2627 | UMHSN00428858 | –9.5 | –9.5 |
| 2577 | UMHSN00152531 | –9.5 | –9.6 | 2628 | UMHSN00009065 | –9.5 | –9.5 |
| 2578 | UMHSN00173522 | –9.5 | –9.6 | 2629 | UMHSN00009838 | –9.4 | –9.5 |
| 2579 | UMHSN00236024 | –9.5 | –9.6 | 2630 | UMHSN00012650 | –9.5 | –9.5 |
| 2580 | UMHSN00244002 | –9.5 | –9.6 | 2631 | UMHSN00014005 | –9.5 | –9.5 |
| 2581 | UMHSN00256181 | –9.6 | –9.6 | 2632 | UMHSN00014772 | –9.5 | –9.5 |
| 2582 | UMHSN00328094 | –9.5 | –9.6 | 2633 | UMHSN00016032 | –9.5 | –9.5 |
| 2583 | UMHSN00005175 | –9.5 | –9.5 | 2634 | UMHSN00019653 | –9.5 | –9.5 |
| 2584 | UMHSN00005543 | –9.4 | –9.5 | 2635 | UMHSN00030888 | –9.5 | –9.5 |
| 2585 | UMHSN00008461 | –9.5 | –9.5 | 2636 | UMHSN00054722 | –9.6 | –9.5 |
| 2586 | UMHSN00010012 | –10.1 | –9.5 | 2637 | UMHSN00056850 | –9.5 | –9.5 |
| 2587 | UMHSN00022765 | –9.4 | –9.5 | 2638 | UMHSN00059840 | –9.6 | –9.5 |
| 2588 | UMHSN00024010 | –9.5 | –9.5 | 2639 | UMHSN00063506 | –9.5 | –9.5 |
| 2589 | UMHSN00032303 | –9.7 | –9.5 | 2640 | UMHSN00067084 | –9.4 | –9.5 |
| 2590 | UMHSN00052410 | –9.4 | –9.5 | 2641 | UMHSN00069109 | –9.4 | –9.5 |
| 2591 | UMHSN00055367 | –9.5 | –9.5 | 2642 | UMHSN00072341 | –9.5 | –9.5 |
| 2592 | UMHSN00057856 | –9.5 | –9.5 | 2643 | UMHSN00073703 | –9.5 | –9.5 |
| 2593 | UMHSN00062282 | –10.0 | –9.5 | 2644 | UMHSN00076813 | –9.5 | –9.5 |
| 2594 | UMHSN00062724 | –9.9 | –9.5 | 2645 | UMHSN00076838 | –9.4 | –9.5 |
| 2595 | UMHSN00063826 | –9.5 | –9.5 | 2646 | UMHSN00080489 | –9.4 | –9.5 |
| 2596 | UMHSN00065958 | –10.3 | –9.5 | 2647 | UMHSN00081562 | –9.5 | –9.5 |
| 2597 | UMHSN00078675 | –9.5 | –9.5 | 2648 | UMHSN00081877 | –9.4 | –9.5 |
| 2598 | UMHSN00081555 | –9.5 | –9.5 | 2649 | UMHSN00082420 | –9.4 | –9.5 |
| 2599 | UMHSN00083571 | –9.4 | –9.5 | 2650 | UMHSN00082737 | –9.5 | –9.5 |
| 2600 | UMHSN00085667 | –9.5 | –9.5 | 2651 | UMHSN00088130 | –9.5 | –9.5 |
| 2601 | UMHSN00093836 | –9.4 | –9.5 | 2652 | UMHSN00095873 | –9.4 | –9.5 |

| No. | SuperNatural II Code | Docking Score (kcal/mol) | | No. | SuperNatural II Code | Docking Score (kcal/mol) | |
| --- | --- | --- | --- | --- | --- | --- | --- |
|  |  | Std.^b^ | Mod.^c^ |  |  | Std.^b^ | Mod.^c^ |
| 2653 | UMHSN00100152 | –9.5 | –9.5 | 2704 | UMHSN00277284 | –9.5 | –9.5 |
| 2654 | UMHSN00101063 | –9.5 | –9.5 | 2705 | UMHSN00290095 | –9.5 | –9.5 |
| 2655 | UMHSN00103402 | –9.5 | –9.5 | 2706 | UMHSN00296507 | –9.5 | –9.5 |
| 2656 | UMHSN00103517 | –9.4 | –9.5 | 2707 | UMHSN00306599 | –9.5 | –9.5 |
| 2657 | UMHSN00105924 | –9.5 | –9.5 | 2708 | UMHSN00313660 | –9.5 | –9.5 |
| 2658 | UMHSN00112323 | –9.4 | –9.5 | 2709 | UMHSN00329073 | –9.7 | –9.5 |
| 2659 | UMHSN00128965 | –9.5 | –9.5 | 2710 | UMHSN00356172 | –9.5 | –9.5 |
| 2660 | UMHSN00134210 | –9.5 | –9.5 | 2711 | UMHSN00363328 | –9.5 | –9.5 |
| 2661 | UMHSN00145209 | –10.2 | –9.5 | 2712 | UMHSN00383767 | –9.5 | –9.5 |
| 2662 | UMHSN00147077 | –9.6 | –9.5 | 2713 | UMHSN00385647 | –9.4 | –9.5 |
| 2663 | UMHSN00151764 | –9.5 | –9.5 | 2714 | UMHSN00399682 | –9.5 | –9.5 |
| 2664 | UMHSN00230934 | –9.5 | –9.5 | 2715 | UMHSN00008396 | –9.5 | –9.5 |
| 2665 | UMHSN00231240 | –9.5 | –9.5 | 2716 | UMHSN00008473 | –9.4 | –9.5 |
| 2666 | UMHSN00256287 | –9.5 | –9.5 | 2717 | UMHSN00008607 | –9.4 | –9.5 |
| 2667 | UMHSN00274507 | –9.5 | –9.5 | 2718 | UMHSN00008795 | –9.5 | –9.5 |
| 2668 | UMHSN00277380 | –9.5 | –9.5 | 2719 | UMHSN00009822 | –9.6 | –9.5 |
| 2669 | UMHSN00289058 | –9.5 | –9.5 | 2720 | UMHSN00010286 | –9.5 | –9.5 |
| 2670 | UMHSN00297702 | –9.5 | –9.5 | 2721 | UMHSN00012301 | –9.5 | –9.5 |
| 2671 | UMHSN00312034 | –9.5 | –9.5 | 2722 | UMHSN00012598 | –9.5 | –9.5 |
| 2672 | UMHSN00355723 | –9.5 | –9.5 | 2723 | UMHSN00014583 | –9.5 | –9.5 |
| 2673 | UMHSN00365770 | –9.5 | –9.5 | 2724 | UMHSN00019829 | –9.4 | –9.5 |
| 2674 | UMHSN00005153 | –10.1 | –9.5 | 2725 | UMHSN00048060 | –9.4 | –9.5 |
| 2675 | UMHSN00009045 | –9.4 | –9.5 | 2726 | UMHSN00056507 | –9.8 | –9.5 |
| 2676 | UMHSN00009755 | –9.5 | –9.5 | 2727 | UMHSN00060235 | –9.4 | –9.5 |
| 2677 | UMHSN00010590 | –9.6 | –9.5 | 2728 | UMHSN00071541 | –9.5 | –9.5 |
| 2678 | UMHSN00016050 | –9.5 | –9.5 | 2729 | UMHSN00077121 | –9.4 | –9.5 |
| 2679 | UMHSN00048733 | –9.5 | –9.5 | 2730 | UMHSN00078750 | –9.5 | –9.5 |
| 2680 | UMHSN00049532 | –9.4 | –9.5 | 2731 | UMHSN00078887 | –9.4 | –9.5 |
| 2681 | UMHSN00050095 | –9.4 | –9.5 | 2732 | UMHSN00081042 | –9.5 | –9.5 |
| 2682 | UMHSN00056963 | –9.5 | –9.5 | 2733 | UMHSN00081433 | –9.5 | –9.5 |
| 2683 | UMHSN00063246 | –9.5 | –9.5 | 2734 | UMHSN00081674 | –9.6 | –9.5 |
| 2684 | UMHSN00072079 | –9.4 | –9.5 | 2735 | UMHSN00090533 | –9.4 | –9.5 |
| 2685 | UMHSN00074932 | –9.4 | –9.5 | 2736 | UMHSN00090626 | –9.5 | –9.5 |
| 2686 | UMHSN00081326 | –9.8 | –9.5 | 2737 | UMHSN00094331 | –9.4 | –9.5 |
| 2687 | UMHSN00083202 | –9.5 | –9.5 | 2738 | UMHSN00097416 | –9.5 | –9.5 |
| 2688 | UMHSN00084665 | –9.5 | –9.5 | 2739 | UMHSN00104293 | –9.5 | –9.5 |
| 2689 | UMHSN00091331 | –9.4 | –9.5 | 2740 | UMHSN00111872 | –9.5 | –9.5 |
| 2690 | UMHSN00098563 | –9.4 | –9.5 | 2741 | UMHSN00113576 | –9.4 | –9.5 |
| 2691 | UMHSN00100847 | –9.5 | –9.5 | 2742 | UMHSN00115326 | –9.5 | –9.5 |
| 2692 | UMHSN00103566 | –9.4 | –9.5 | 2743 | UMHSN00115849 | –9.5 | –9.5 |
| 2693 | UMHSN00103794 | –9.5 | –9.5 | 2744 | UMHSN00140848 | –9.4 | –9.5 |
| 2694 | UMHSN00104875 | –9.4 | –9.5 | 2745 | UMHSN00144700 | –9.5 | –9.5 |
| 2695 | UMHSN00116037 | –9.5 | –9.5 | 2746 | UMHSN00152453 | –9.5 | –9.5 |
| 2696 | UMHSN00146637 | –9.4 | –9.5 | 2747 | UMHSN00153499 | –9.4 | –9.5 |
| 2697 | UMHSN00150670 | –9.5 | –9.5 | 2748 | UMHSN00153567 | –9.4 | –9.5 |
| 2698 | UMHSN00153998 | –9.6 | –9.5 | 2749 | UMHSN00160924 | –9.5 | –9.5 |
| 2699 | UMHSN00154701 | –9.4 | –9.5 | 2750 | UMHSN00162707 | –9.5 | –9.5 |
| 2700 | UMHSN00160614 | –9.4 | –9.5 | 2751 | UMHSN00220556 | –9.5 | –9.5 |
| 2701 | UMHSN00227290 | –9.5 | –9.5 | 2752 | UMHSN00254067 | –9.5 | –9.5 |
| 2702 | UMHSN00228565 | –9.5 | –9.5 | 2753 | UMHSN00277058 | –9.5 | –9.5 |
| 2703 | UMHSN00241323 | –9.5 | –9.5 | 2754 | UMHSN00278257 | –9.5 | –9.5 |

**Table.** *Continued.*

| No. | SuperNatural II Code | Docking Score (kcal/mol) | | No. | SuperNatural II Code | Docking Score (kcal/mol) | |
| --- | --- | --- | --- | --- | --- | --- | --- |
|  |  | Std.^b^ | Mod.^c^ |  |  | Std.^b^ | Mod.^c^ |
| 2755 | UMHSN00296312 | –9.4 | –9.5 | 2806 | UMHSN00076790 | –9.9 | –9.5 |
| 2756 | UMHSN00297513 | –9.4 | –9.5 | 2807 | UMHSN00078423 | –9.4 | –9.5 |
| 2757 | UMHSN00351836 | –9.5 | –9.5 | 2808 | UMHSN00081082 | –9.7 | –9.5 |
| 2758 | UMHSN00372252 | –9.5 | –9.5 | 2809 | UMHSN00084105 | –9.9 | –9.5 |
| 2759 | UMHSN00400101 | –9.4 | –9.5 | 2810 | UMHSN00086444 | –9.5 | –9.5 |
| 2760 | UMHSN00004560 | –9.4 | –9.5 | 2811 | UMHSN00087978 | –9.4 | –9.5 |
| 2761 | UMHSN00009200 | –9.5 | –9.5 | 2812 | UMHSN00096076 | –9.4 | –9.5 |
| 2762 | UMHSN00009364 | –9.5 | –9.5 | 2813 | UMHSN00096487 | –9.4 | –9.5 |
| 2763 | UMHSN00009800 | –9.5 | –9.5 | 2814 | UMHSN00098458 | –9.5 | –9.5 |
| 2764 | UMHSN00012292 | –9.5 | –9.5 | 2815 | UMHSN00116130 | –9.5 | –9.5 |
| 2765 | UMHSN00014344 | –9.4 | –9.5 | 2816 | UMHSN00120521 | –9.4 | –9.5 |
| 2766 | UMHSN00015033 | –9.5 | –9.5 | 2817 | UMHSN00132523 | –9.4 | –9.5 |
| 2767 | UMHSN00021340 | –9.5 | –9.5 | 2818 | UMHSN00147858 | –9.5 | –9.5 |
| 2768 | UMHSN00063992 | –9.5 | –9.5 | 2819 | UMHSN00150648 | –9.5 | –9.5 |
| 2769 | UMHSN00069558 | –9.5 | –9.5 | 2820 | UMHSN00154279 | –9.4 | –9.5 |
| 2770 | UMHSN00086277 | –9.4 | –9.5 | 2821 | UMHSN00178199 | –9.5 | –9.5 |
| 2771 | UMHSN00090407 | –9.4 | –9.5 | 2822 | UMHSN00224646 | –9.5 | –9.5 |
| 2772 | UMHSN00094321 | –9.5 | –9.5 | 2823 | UMHSN00228913 | –9.4 | –9.5 |
| 2773 | UMHSN00095425 | –9.4 | –9.5 | 2824 | UMHSN00235331 | –9.5 | –9.5 |
| 2774 | UMHSN00096214 | –9.4 | –9.5 | 2825 | UMHSN00259408 | –9.5 | –9.5 |
| 2775 | UMHSN00101735 | –9.4 | –9.5 | 2826 | UMHSN00273397 | –9.4 | –9.5 |
| 2776 | UMHSN00102102 | –9.5 | –9.5 | 2827 | UMHSN00276563 | –9.5 | –9.5 |
| 2777 | UMHSN00104030 | –9.5 | –9.5 | 2828 | UMHSN00297902 | –9.5 | –9.5 |
| 2778 | UMHSN00104588 | –9.4 | –9.5 | 2829 | UMHSN00298029 | –9.5 | –9.5 |
| 2779 | UMHSN00114072 | –9.4 | –9.5 | 2830 | UMHSN00317034 | –9.4 | –9.5 |
| 2780 | UMHSN00132053 | –9.4 | –9.5 | 2831 | UMHSN00331556 | –9.4 | –9.5 |
| 2781 | UMHSN00150743 | –9.5 | –9.5 | 2832 | UMHSN00338792 | –9.4 | –9.5 |
| 2782 | UMHSN00151975 | –9.4 | –9.5 | 2833 | UMHSN00341514 | –9.6 | –9.5 |
| 2783 | UMHSN00153292 | –9.5 | –9.5 | 2834 | UMHSN00342689 | –9.5 | –9.5 |
| 2784 | UMHSN00154745 | –9.5 | –9.5 | 2835 | UMHSN00343969 | –9.5 | –9.5 |
| 2785 | UMHSN00161049 | –9.4 | –9.5 | 2836 | UMHSN00350337 | –9.4 | –9.5 |
| 2786 | UMHSN00259817 | –9.5 | –9.5 | 2837 | UMHSN00396441 | –9.5 | –9.5 |
| 2787 | UMHSN00271036 | –9.4 | –9.5 | 2838 | UMHSN00397874 | –9.4 | –9.5 |
| 2788 | UMHSN00283379 | –9.5 | –9.5 | 2839 | UMHSN00004645 | –9.4 | –9.5 |
| 2789 | UMHSN00292475 | –9.4 | –9.5 | 2840 | UMHSN00008139 | –9.5 | –9.5 |
| 2790 | UMHSN00292700 | –9.5 | –9.5 | 2841 | UMHSN00008477 | –9.5 | –9.5 |
| 2791 | UMHSN00372938 | –9.5 | –9.5 | 2842 | UMHSN00012316 | –9.4 | –9.5 |
| 2792 | UMHSN00393747 | –9.4 | –9.5 | 2843 | UMHSN00014442 | –9.4 | –9.5 |
| 2793 | UMHSN00008339 | –9.5 | –9.5 | 2844 | UMHSN00014564 | –9.4 | –9.5 |
| 2794 | UMHSN00010275 | –9.5 | –9.5 | 2845 | UMHSN00014932 | –9.4 | –9.5 |
| 2795 | UMHSN00010824 | –9.4 | –9.5 | 2846 | UMHSN00014933 | –9.5 | –9.5 |
| 2796 | UMHSN00012116 | –9.5 | –9.5 | 2847 | UMHSN00026342 | –9.5 | –9.5 |
| 2797 | UMHSN00012814 | –9.7 | –9.5 | 2848 | UMHSN00035502 | –9.4 | –9.5 |
| 2798 | UMHSN00013929 | –9.4 | –9.5 | 2849 | UMHSN00041077 | –9.5 | –9.5 |
| 2799 | UMHSN00028304 | –9.4 | –9.5 | 2850 | UMHSN00056977 | –9.4 | –9.5 |
| 2800 | UMHSN00031215 | –10.0 | –9.5 | 2851 | UMHSN00058581 | –9.5 | –9.5 |
| 2801 | UMHSN00031976 | –9.4 | –9.5 | 2852 | UMHSN00063672 | –9.4 | –9.5 |
| 2802 | UMHSN00054556 | –9.4 | –9.5 | 2853 | UMHSN00066557 | –9.4 | –9.5 |
| 2803 | UMHSN00057152 | –9.4 | –9.5 | 2854 | UMHSN00071788 | –9.4 | –9.5 |
| 2804 | UMHSN00057667 | –9.5 | –9.5 | 2855 | UMHSN00078808 | –9.5 | –9.5 |
| 2805 | UMHSN00063484 | –9.5 | –9.5 | 2856 | UMHSN00078883 | –9.4 | –9.5 |

| No. | SuperNatural II Code | Docking Score (kcal/mol) | | No. | SuperNatural II Code | Docking Score (kcal/mol) | |
| --- | --- | --- | --- | --- | --- | --- | --- |
|  |  | Std.^b^ | Mod.^c^ |  |  | Std.^b^ | Mod.^c^ |
| 2857 | UMHSN00081632 | –9.5 | –9.5 | 2908 | UMHSN00342287 | –9.5 | –9.5 |
| 2858 | UMHSN00104729 | –9.6 | –9.5 | 2909 | UMHSN00343073 | –9.5 | –9.5 |
| 2859 | UMHSN00113244 | –9.8 | –9.5 | 2910 | UMHSN00348137 | –9.5 | –9.5 |
| 2860 | UMHSN00126506 | –9.4 | –9.5 | 2911 | UMHSN00359248 | –9.5 | –9.5 |
| 2861 | UMHSN00152708 | –9.4 | –9.5 | 2912 | UMHSN00384019 | –9.5 | –9.5 |
| 2862 | UMHSN00153299 | –9.5 | –9.5 | 2913 | UMHSN00398501 | –9.5 | –9.5 |
| 2863 | UMHSN00162816 | –9.4 | –9.5 | 2914 | UMHSN00400339 | –9.5 | –9.5 |
| 2864 | UMHSN00164889 | –9.5 | –9.5 | 2915 | UMHSN00008505 | –9.4 | –9.5 |
| 2865 | UMHSN00169252 | –9.5 | –9.5 | 2916 | UMHSN00011737 | –9.4 | –9.5 |
| 2866 | UMHSN00215198 | –9.5 | –9.5 | 2917 | UMHSN00014540 | –9.4 | –9.5 |
| 2867 | UMHSN00229677 | –9.5 | –9.5 | 2918 | UMHSN00014840 | –9.4 | –9.5 |
| 2868 | UMHSN00256398 | –9.5 | –9.5 | 2919 | UMHSN00015950 | –9.5 | –9.5 |
| 2869 | UMHSN00258084 | –9.5 | –9.5 | 2920 | UMHSN00054867 | –9.5 | –9.5 |
| 2870 | UMHSN00280397 | –9.5 | –9.5 | 2921 | UMHSN00056642 | –9.4 | –9.5 |
| 2871 | UMHSN00280456 | –9.5 | –9.5 | 2922 | UMHSN00062941 | –9.4 | –9.5 |
| 2872 | UMHSN00308385 | –9.5 | –9.5 | 2923 | UMHSN00086434 | –9.4 | –9.5 |
| 2873 | UMHSN00338279 | –9.5 | –9.5 | 2924 | UMHSN00089691 | –9.4 | –9.5 |
| 2874 | UMHSN00340420 | –9.5 | –9.5 | 2925 | UMHSN00089806 | –9.4 | –9.5 |
| 2875 | UMHSN00347168 | –9.4 | –9.5 | 2926 | UMHSN00091380 | –9.4 | –9.5 |
| 2876 | UMHSN00372826 | –9.5 | –9.5 | 2927 | UMHSN00091770 | –9.5 | –9.5 |
| 2877 | UMHSN00005426 | –9.4 | –9.5 | 2928 | UMHSN00094291 | –9.4 | –9.5 |
| 2878 | UMHSN00008497 | –9.5 | –9.5 | 2929 | UMHSN00105808 | –9.5 | –9.5 |
| 2879 | UMHSN00009249 | –9.5 | –9.5 | 2930 | UMHSN00107136 | –9.5 | –9.5 |
| 2880 | UMHSN00009288 | –9.4 | –9.5 | 2931 | UMHSN00112192 | –9.4 | –9.5 |
| 2881 | UMHSN00015055 | –9.4 | –9.5 | 2932 | UMHSN00136317 | –9.5 | –9.5 |
| 2882 | UMHSN00015855 | –9.5 | –9.5 | 2933 | UMHSN00150072 | –10.0 | –9.5 |
| 2883 | UMHSN00017635 | –9.4 | –9.5 | 2934 | UMHSN00150349 | –9.5 | –9.5 |
| 2884 | UMHSN00021752 | –9.4 | –9.5 | 2935 | UMHSN00153478 | –9.4 | –9.5 |
| 2885 | UMHSN00026479 | –9.5 | –9.5 | 2936 | UMHSN00154045 | –9.4 | –9.5 |
| 2886 | UMHSN00058432 | –9.5 | –9.5 | 2937 | UMHSN00167057 | –9.5 | –9.5 |
| 2887 | UMHSN00064912 | –9.5 | –9.5 | 2938 | UMHSN00170112 | –9.5 | –9.5 |
| 2888 | UMHSN00073794 | –9.4 | –9.5 | 2939 | UMHSN00174040 | –9.5 | –9.5 |
| 2889 | UMHSN00074447 | –9.5 | –9.5 | 2940 | UMHSN00215031 | –9.5 | –9.5 |
| 2890 | UMHSN00081494 | –9.4 | –9.5 | 2941 | UMHSN00220501 | –9.5 | –9.5 |
| 2891 | UMHSN00082411 | –9.5 | –9.5 | 2942 | UMHSN00246275 | –9.5 | –9.5 |
| 2892 | UMHSN00088515 | –9.4 | –9.5 | 2943 | UMHSN00264415 | –9.6 | –9.5 |
| 2893 | UMHSN00089690 | –9.4 | –9.5 | 2944 | UMHSN00272953 | –10.0 | –9.5 |
| 2894 | UMHSN00100813 | –9.5 | –9.5 | 2945 | UMHSN00274189 | –9.7 | –9.5 |
| 2895 | UMHSN00103822 | –9.5 | –9.5 | 2946 | UMHSN00280412 | –9.4 | –9.5 |
| 2896 | UMHSN00103996 | –9.6 | –9.5 | 2947 | UMHSN00293412 | –9.4 | –9.5 |
| 2897 | UMHSN00104284 | –9.5 | –9.5 | 2948 | UMHSN00297951 | –9.4 | –9.5 |
| 2898 | UMHSN00104661 | –9.5 | –9.5 | 2949 | UMHSN00339005 | –9.5 | –9.5 |
| 2899 | UMHSN00112038 | –9.4 | –9.5 | 2950 | UMHSN00380142 | –9.5 | –9.5 |
| 2900 | UMHSN00121637 | –9.5 | –9.5 | 2951 | UMHSN00401533 | –9.5 | –9.5 |
| 2901 | UMHSN00151751 | –9.4 | –9.5 | 2952 | UMHSN00008048 | –9.4 | –9.5 |
| 2902 | UMHSN00168438 | –9.5 | –9.5 | 2953 | UMHSN00010242 | –9.4 | –9.5 |
| 2903 | UMHSN00214026 | –9.4 | –9.5 | 2954 | UMHSN00010281 | –9.4 | –9.5 |
| 2904 | UMHSN00214736 | –9.5 | –9.5 | 2955 | UMHSN00013530 | –9.6 | –9.5 |
| 2905 | UMHSN00303446 | –9.5 | –9.5 | 2956 | UMHSN00014018 | –9.4 | –9.5 |
| 2906 | UMHSN00328995 | –10.4 | –9.5 | 2957 | UMHSN00017268 | –9.4 | –9.5 |
| 2907 | UMHSN00338181 | –9.5 | –9.5 | 2958 | UMHSN00024183 | –9.4 | –9.5 |

| No. | SuperNatural II Code | Docking Score (kcal/mol) | | No. | SuperNatural II Code | Docking Score (kcal/mol) | |
| --- | --- | --- | --- | --- | --- | --- | --- |
|  |  | Std.^b^ | Mod.^c^ |  |  | Std.^b^ | Mod.^c^ |
| 2959 | UMHSN00026492 | –9.4 | –9.5 | 3010 | UMHSN00150189 | –9.6 | –9.4 |
| 2960 | UMHSN00049751 | –9.4 | –9.5 | 3011 | UMHSN00153382 | –9.4 | –9.4 |
| 2961 | UMHSN00059252 | –9.4 | –9.5 | 3012 | UMHSN00154931 | –9.4 | –9.4 |
| 2962 | UMHSN00070622 | –9.4 | –9.5 | 3013 | UMHSN00154946 | –9.4 | –9.4 |
| 2963 | UMHSN00071322 | –9.5 | –9.5 | 3014 | UMHSN00155448 | –9.4 | –9.4 |
| 2964 | UMHSN00074708 | –9.4 | –9.5 | 3015 | UMHSN00231491 | –9.4 | –9.4 |
| 2965 | UMHSN00076593 | –9.4 | –9.5 | 3016 | UMHSN00241469 | –9.5 | –9.4 |
| 2966 | UMHSN00076723 | –9.5 | –9.5 | 3017 | UMHSN00251080 | –9.4 | –9.4 |
| 2967 | UMHSN00078534 | –10.5 | –9.5 | 3018 | UMHSN00269093 | –9.4 | –9.4 |
| 2968 | UMHSN00085398 | –9.4 | –9.5 | 3019 | UMHSN00271427 | –9.4 | –9.4 |
| 2969 | UMHSN00121445 | –9.4 | –9.5 | 3020 | UMHSN00297190 | –9.4 | –9.4 |
| 2970 | UMHSN00125290 | –9.4 | –9.5 | 3021 | UMHSN00313726 | –9.4 | –9.4 |
| 2971 | UMHSN00162359 | –9.8 | –9.5 | 3022 | UMHSN00343711 | –9.4 | –9.4 |
| 2972 | UMHSN00255145 | –9.4 | –9.5 | 3023 | UMHSN00366028 | –9.4 | –9.4 |
| 2973 | UMHSN00290746 | –9.4 | –9.5 | 3024 | UMHSN00373499 | –9.7 | –9.4 |
| 2974 | UMHSN00298878 | –9.4 | –9.5 | 3025 | UMHSN00003688 | –9.4 | –9.4 |
| 2975 | UMHSN00323841 | –9.5 | –9.5 | 3026 | UMHSN00008776 | –9.4 | –9.4 |
| 2976 | UMHSN00371146 | –9.5 | –9.5 | 3027 | UMHSN00015866 | –9.4 | –9.4 |
| 2977 | UMHSN00391788 | –9.4 | –9.5 | 3028 | UMHSN00019658 | –9.4 | –9.4 |
| 2978 | UMHSN00400243 | –9.4 | –9.5 | 3029 | UMHSN00045331 | –9.4 | –9.4 |
| 2979 | UMHSN00401038 | –9.4 | –9.5 | 3030 | UMHSN00055445 | –9.4 | –9.4 |
| 2980 | UMHSN00004381 | –9.4 | –9.4 | 3031 | UMHSN00058000 | –9.4 | –9.4 |
| 2981 | UMHSN00005278 | –9.7 | –9.4 | 3032 | UMHSN00060878 | –9.4 | –9.4 |
| 2982 | UMHSN00005491 | –9.4 | –9.4 | 3033 | UMHSN00068508 | –9.4 | –9.4 |
| 2983 | UMHSN00008490 | –9.4 | –9.4 | 3034 | UMHSN00073789 | –9.4 | –9.4 |
| 2984 | UMHSN00008592 | –9.4 | –9.4 | 3035 | UMHSN00081662 | –9.4 | –9.4 |
| 2985 | UMHSN00008659 | –9.4 | –9.4 | 3036 | UMHSN00100801 | –9.4 | –9.4 |
| 2986 | UMHSN00008707 | –9.4 | –9.4 | 3037 | UMHSN00104429 | –9.4 | –9.4 |
| 2987 | UMHSN00008782 | –9.4 | –9.4 | 3038 | UMHSN00134071 | –9.4 | –9.4 |
| 2988 | UMHSN00009157 | –9.4 | –9.4 | 3039 | UMHSN00148898 | –9.4 | –9.4 |
| 2989 | UMHSN00012565 | –9.4 | –9.4 | 3040 | UMHSN00149918 | –9.6 | –9.4 |
| 2990 | UMHSN00014813 | –9.4 | –9.4 | 3041 | UMHSN00160759 | –9.4 | –9.4 |
| 2991 | UMHSN00025760 | –9.5 | –9.4 | 3042 | UMHSN00224365 | –9.4 | –9.4 |
| 2992 | UMHSN00026483 | –9.4 | –9.4 | 3043 | UMHSN00231590 | –9.4 | –9.4 |
| 2993 | UMHSN00042160 | –9.4 | –9.4 | 3044 | UMHSN00236450 | –9.4 | –9.4 |
| 2994 | UMHSN00052193 | –10.0 | –9.4 | 3045 | UMHSN00263765 | –9.4 | –9.4 |
| 2995 | UMHSN00053338 | –9.4 | –9.4 | 3046 | UMHSN00271721 | –9.4 | –9.4 |
| 2996 | UMHSN00055045 | –9.5 | –9.4 | 3047 | UMHSN00290889 | –9.4 | –9.4 |
| 2997 | UMHSN00056533 | –9.4 | –9.4 | 3048 | UMHSN00308517 | –9.4 | –9.4 |
| 2998 | UMHSN00063661 | –9.4 | –9.4 | 3049 | UMHSN00322795 | –9.4 | –9.4 |
| 2999 | UMHSN00065438 | –9.4 | –9.4 | 3050 | UMHSN00376955 | –9.4 | –9.4 |
| 3000 | UMHSN00070321 | –9.4 | –9.4 | 3051 | UMHSN00387544 | –9.4 | –9.4 |
| 3001 | UMHSN00074006 | –9.4 | –9.4 | 3052 | UMHSN00007964 | –9.4 | –9.4 |
| 3002 | UMHSN00076850 | –9.4 | –9.4 | 3053 | UMHSN00008464 | –9.4 | –9.4 |
| 3003 | UMHSN00077986 | –9.4 | –9.4 | 3054 | UMHSN00008612 | –9.5 | –9.4 |
| 3004 | UMHSN00089309 | –9.4 | –9.4 | 3055 | UMHSN00010827 | –9.4 | –9.4 |
| 3005 | UMHSN00090269 | –9.5 | –9.4 | 3056 | UMHSN00011013 | –9.4 | –9.4 |
| 3006 | UMHSN00091550 | –9.4 | –9.4 | 3057 | UMHSN00014879 | –9.6 | –9.4 |
| 3007 | UMHSN00091903 | –9.4 | –9.4 | 3058 | UMHSN00014990 | –9.9 | –9.4 |
| 3008 | UMHSN00112615 | –9.4 | –9.4 | 3059 | UMHSN00015022 | –9.7 | –9.4 |
| 3009 | UMHSN00116162 | –10.4 | –9.4 | 3060 | UMHSN00015462 | –9.4 | –9.4 |

| No. | SuperNatural II Code | Docking Score (kcal/mol) | | No. | SuperNatural II Code | Docking Score (kcal/mol) | |
| --- | --- | --- | --- | --- | --- | --- | --- |
|  |  | Std.^b^ | Mod.^c^ |  |  | Std.^b^ | Mod.^c^ |
| 3061 | UMHSN00018620 | –9.4 | –9.4 | 3112 | UMHSN00078209 | –9.6 | –9.4 |
| 3062 | UMHSN00019407 | –9.4 | –9.4 | 3113 | UMHSN00081810 | –9.4 | –9.4 |
| 3063 | UMHSN00021937 | –9.4 | –9.4 | 3114 | UMHSN00089796 | –9.4 | –9.4 |
| 3064 | UMHSN00050934 | –9.4 | –9.4 | 3115 | UMHSN00126762 | –9.4 | –9.4 |
| 3065 | UMHSN00054529 | –9.4 | –9.4 | 3116 | UMHSN00128371 | –9.4 | –9.4 |
| 3066 | UMHSN00058233 | –9.4 | –9.4 | 3117 | UMHSN00152382 | –9.4 | –9.4 |
| 3067 | UMHSN00060346 | –9.4 | –9.4 | 3118 | UMHSN00161565 | –9.4 | –9.4 |
| 3068 | UMHSN00064570 | –9.4 | –9.4 | 3119 | UMHSN00168471 | –9.4 | –9.4 |
| 3069 | UMHSN00071510 | –9.4 | –9.4 | 3120 | UMHSN00169531 | –9.7 | –9.4 |
| 3070 | UMHSN00072386 | –9.4 | –9.4 | 3121 | UMHSN00232410 | –9.4 | –9.4 |
| 3071 | UMHSN00072645 | –10.1 | –9.4 | 3122 | UMHSN00236504 | –9.4 | –9.4 |
| 3072 | UMHSN00082982 | –9.4 | –9.4 | 3123 | UMHSN00251095 | –9.4 | –9.4 |
| 3073 | UMHSN00087352 | –9.4 | –9.4 | 3124 | UMHSN00267881 | –9.4 | –9.4 |
| 3074 | UMHSN00090818 | –9.4 | –9.4 | 3125 | UMHSN00277628 | –9.4 | –9.4 |
| 3075 | UMHSN00115130 | –9.5 | –9.4 | 3126 | UMHSN00277999 | –9.4 | –9.4 |
| 3076 | UMHSN00115256 | –9.4 | –9.4 | 3127 | UMHSN00281431 | –9.4 | –9.4 |
| 3077 | UMHSN00115932 | –9.4 | –9.4 | 3128 | UMHSN00285055 | –9.4 | –9.4 |
| 3078 | UMHSN00125661 | –9.4 | –9.4 | 3129 | UMHSN00427169 | –9.6 | –9.4 |
| 3079 | UMHSN00131383 | –9.4 | –9.4 | 3130 | UMHSN00004344 | –9.4 | –9.4 |
| 3080 | UMHSN00146856 | –9.4 | –9.4 | 3131 | UMHSN00008504 | –9.4 | –9.4 |
| 3081 | UMHSN00150720 | –9.4 | –9.4 | 3132 | UMHSN00008506 | –9.5 | –9.4 |
| 3082 | UMHSN00150977 | –10.4 | –9.4 | 3133 | UMHSN00010810 | –9.4 | –9.4 |
| 3083 | UMHSN00152541 | –9.4 | –9.4 | 3134 | UMHSN00011843 | –9.4 | –9.4 |
| 3084 | UMHSN00220487 | –9.4 | –9.4 | 3135 | UMHSN00012508 | –9.5 | –9.4 |
| 3085 | UMHSN00235961 | –9.4 | –9.4 | 3136 | UMHSN00013133 | –9.4 | –9.4 |
| 3086 | UMHSN00243188 | –9.4 | –9.4 | 3137 | UMHSN00013308 | –9.4 | –9.4 |
| 3087 | UMHSN00259067 | –9.5 | –9.4 | 3138 | UMHSN00015036 | –9.4 | –9.4 |
| 3088 | UMHSN00264529 | –9.5 | –9.4 | 3139 | UMHSN00015971 | –9.4 | –9.4 |
| 3089 | UMHSN00267489 | –9.4 | –9.4 | 3140 | UMHSN00022705 | –9.4 | –9.4 |
| 3090 | UMHSN00332409 | –9.4 | –9.4 | 3141 | UMHSN00024146 | –9.4 | –9.4 |
| 3091 | UMHSN00338086 | –9.4 | –9.4 | 3142 | UMHSN00028274 | –9.5 | –9.4 |
| 3092 | UMHSN00344432 | –9.4 | –9.4 | 3143 | UMHSN00030620 | –9.4 | –9.4 |
| 3093 | UMHSN00349823 | –9.4 | –9.4 | 3144 | UMHSN00039189 | –9.4 | –9.4 |
| 3094 | UMHSN00365567 | –9.4 | –9.4 | 3145 | UMHSN00076423 | –9.5 | –9.4 |
| 3095 | UMHSN00386943 | –9.4 | –9.4 | 3146 | UMHSN00076875 | –9.4 | –9.4 |
| 3096 | UMHSN00387843 | –9.4 | –9.4 | 3147 | UMHSN00091564 | –9.4 | –9.4 |
| 3097 | UMHSN00004382 | –9.4 | –9.4 | 3148 | UMHSN00102280 | –9.4 | –9.4 |
| 3098 | UMHSN00007968 | –9.4 | –9.4 | 3149 | UMHSN00106798 | –9.4 | –9.4 |
| 3099 | UMHSN00008657 | –9.4 | –9.4 | 3150 | UMHSN00115421 | –9.4 | –9.4 |
| 3100 | UMHSN00009186 | –9.4 | –9.4 | 3151 | UMHSN00118904 | –9.4 | –9.4 |
| 3101 | UMHSN00009740 | –9.4 | –9.4 | 3152 | UMHSN00132371 | –9.4 | –9.4 |
| 3102 | UMHSN00009968 | –9.4 | –9.4 | 3153 | UMHSN00147819 | –9.4 | –9.4 |
| 3103 | UMHSN00011916 | –9.4 | –9.4 | 3154 | UMHSN00161184 | –9.4 | –9.4 |
| 3104 | UMHSN00015052 | –9.4 | –9.4 | 3155 | UMHSN00227114 | –9.4 | –9.4 |
| 3105 | UMHSN00016233 | –9.4 | –9.4 | 3156 | UMHSN00229905 | –9.4 | –9.4 |
| 3106 | UMHSN00017629 | –9.4 | –9.4 | 3157 | UMHSN00269492 | –9.4 | –9.4 |
| 3107 | UMHSN00033199 | –9.4 | –9.4 | 3158 | UMHSN00277048 | –9.4 | –9.4 |
| 3108 | UMHSN00048203 | –9.4 | –9.4 | 3159 | UMHSN00318426 | –9.4 | –9.4 |
| 3109 | UMHSN00053300 | –9.5 | –9.4 | 3160 | UMHSN00330281 | –9.4 | –9.4 |
| 3110 | UMHSN00065272 | –9.4 | –9.4 | 3161 | UMHSN00343138 | –9.4 | –9.4 |
| 3111 | UMHSN00078176 | –9.8 | –9.4 | 3162 | UMHSN00371648 | –9.4 | –9.4 |

| No. | SuperNatural II Code | Docking Score (kcal/mol) | | No. | SuperNatural II Code | Docking Score (kcal/mol) | |
| --- | --- | --- | --- | --- | --- | --- | --- |
|  |  | Std.^b^ | Mod.^c^ |  |  | Std.^b^ | Mod.^c^ |
| 3163 | UMHSN00380428 | –9.4 | –9.4 | 3214 | UMHSN00336129 | –9.4 | –9.4 |
| 3164 | UMHSN00004379 | –9.4 | –9.4 | 3215 | UMHSN00351256 | –9.4 | –9.4 |
| 3165 | UMHSN00008163 | –9.7 | –9.4 | 3216 | UMHSN00360708 | –9.4 | –9.4 |
| 3166 | UMHSN00008488 | –9.4 | –9.4 | 3217 | UMHSN00362649 | –9.4 | –9.4 |
| 3167 | UMHSN00009255 | –9.4 | –9.4 | 3218 | UMHSN00396500 | –9.5 | –9.4 |
| 3168 | UMHSN00012293 | –9.4 | –9.4 | 3219 | UMHSN00011695 | –9.4 | –9.4 |
| 3169 | UMHSN00013565 | –9.4 | –9.4 | 3220 | UMHSN00015947 | –9.4 | –9.4 |
| 3170 | UMHSN00015867 | –10.1 | –9.4 | 3221 | UMHSN00018205 | –10.1 | –9.4 |
| 3171 | UMHSN00017986 | –9.4 | –9.4 | 3222 | UMHSN00028915 | –9.4 | –9.4 |
| 3172 | UMHSN00019434 | –9.4 | –9.4 | 3223 | UMHSN00034963 | –9.4 | –9.4 |
| 3173 | UMHSN00024163 | –9.4 | –9.4 | 3224 | UMHSN00057112 | –9.6 | –9.4 |
| 3174 | UMHSN00063483 | –9.4 | –9.4 | 3225 | UMHSN00057660 | –9.4 | –9.4 |
| 3175 | UMHSN00078030 | –9.7 | –9.4 | 3226 | UMHSN00067043 | –9.4 | –9.4 |
| 3176 | UMHSN00081618 | –9.4 | –9.4 | 3227 | UMHSN00069644 | –9.4 | –9.4 |
| 3177 | UMHSN00085082 | –9.4 | –9.4 | 3228 | UMHSN00076736 | –9.4 | –9.4 |
| 3178 | UMHSN00092221 | –9.6 | –9.4 | 3229 | UMHSN00084340 | –9.4 | –9.4 |
| 3179 | UMHSN00103919 | –9.9 | –9.4 | 3230 | UMHSN00095672 | –9.4 | –9.4 |
| 3180 | UMHSN00106467 | –9.4 | –9.4 | 3231 | UMHSN00100130 | –9.4 | –9.4 |
| 3181 | UMHSN00126808 | –9.4 | –9.4 | 3232 | UMHSN00104023 | –9.4 | –9.4 |
| 3182 | UMHSN00148809 | –9.6 | –9.4 | 3233 | UMHSN00105953 | –9.4 | –9.4 |
| 3183 | UMHSN00149538 | –9.4 | –9.4 | 3234 | UMHSN00106545 | –9.4 | –9.4 |
| 3184 | UMHSN00170968 | –9.4 | –9.4 | 3235 | UMHSN00148240 | –9.5 | –9.4 |
| 3185 | UMHSN00251952 | –9.4 | –9.4 | 3236 | UMHSN00151771 | –9.4 | –9.4 |
| 3186 | UMHSN00359019 | –9.4 | –9.4 | 3237 | UMHSN00154922 | –9.4 | –9.4 |
| 3187 | UMHSN00369434 | –9.4 | –9.4 | 3238 | UMHSN00159953 | –9.4 | –9.4 |
| 3188 | UMHSN00391849 | –10.6 | –9.4 | 3239 | UMHSN00160251 | –9.6 | –9.4 |
| 3189 | UMHSN00428467 | –9.4 | –9.4 | 3240 | UMHSN00230699 | –9.4 | –9.4 |
| 3190 | UMHSN00005183 | –9.4 | –9.4 | 3241 | UMHSN00251798 | –9.4 | –9.4 |
| 3191 | UMHSN00008465 | –9.4 | –9.4 | 3242 | UMHSN00263701 | –9.4 | –9.4 |
| 3192 | UMHSN00012122 | –9.4 | –9.4 | 3243 | UMHSN00325691 | –9.7 | –9.4 |
| 3193 | UMHSN00012137 | –9.4 | –9.4 | 3244 | UMHSN00374495 | –9.4 | –9.4 |
| 3194 | UMHSN00014051 | –9.7 | –9.4 | 3245 | UMHSN00427152 | –10.1 | –9.4 |
| 3195 | UMHSN00014074 | –9.4 | –9.4 | 3246 | UMHSN00008140 | –9.5 | –9.4 |
| 3196 | UMHSN00014765 | –9.4 | –9.4 | 3247 | UMHSN00031211 | –9.5 | –9.4 |
| 3197 | UMHSN00019765 | –9.4 | –9.4 | 3248 | UMHSN00063529 | –9.6 | –9.4 |
| 3198 | UMHSN00033676 | –9.4 | –9.4 | 3249 | UMHSN00094322 | –9.4 | –9.4 |
| 3199 | UMHSN00050650 | –9.4 | –9.4 | 3250 | UMHSN00104724 | –9.4 | –9.4 |
| 3200 | UMHSN00074019 | –9.4 | –9.4 | 3251 | UMHSN00148805 | –9.4 | –9.4 |
| 3201 | UMHSN00076776 | –9.4 | –9.4 | 3252 | UMHSN00150222 | –9.5 | –9.4 |
| 3202 | UMHSN00078074 | –9.6 | –9.4 | 3253 | UMHSN00230318 | –9.4 | –9.4 |
| 3203 | UMHSN00078486 | –9.4 | –9.4 | 3254 | UMHSN00262564 | –9.4 | –9.4 |
| 3204 | UMHSN00081622 | –9.4 | –9.4 | 3255 | UMHSN00287503 | –9.4 | –9.4 |
| 3205 | UMHSN00130445 | –9.4 | –9.4 | 3256 | UMHSN00288084 | –9.4 | –9.4 |
| 3206 | UMHSN00140947 | –9.4 | –9.4 | 3257 | UMHSN00013344 | –9.4 | –9.4 |
| 3207 | UMHSN00162323 | –9.4 | –9.4 | 3258 | UMHSN00057076 | –9.4 | –9.4 |
| 3208 | UMHSN00216238 | –9.4 | –9.4 | 3259 | UMHSN00112463 | –9.4 | –9.4 |
| 3209 | UMHSN00263252 | –9.4 | –9.4 | 3260 | UMHSN00136916 | –9.4 | –9.4 |
| 3210 | UMHSN00266252 | –9.4 | –9.4 | 3261 | UMHSN00146841 | –9.7 | –9.4 |
| 3211 | UMHSN00281316 | –9.4 | –9.4 | 3262 | UMHSN00150722 | –9.6 | –9.4 |
| 3212 | UMHSN00284706 | –9.4 | –9.4 | 3263 | UMHSN00173531 | –9.4 | –9.4 |
| 3213 | UMHSN00310076 | –9.4 | –9.4 | 3264 | UMHSN00225209 | –9.4 | –9.4 |

| No. | SuperNatural II Code | Docking Score (kcal/mol) | | No. | SuperNatural II Code | Docking Score (kcal/mol) | |
| --- | --- | --- | --- | --- | --- | --- | --- |
|  |  | Std.^b^ | Mod.^c^ |  |  | Std.^b^ | Mod.^c^ |
| 3265 | UMHSN00283247 | –9.4 | –9.4 | 3316 | UMHSN00011780 | –9.4 | –9.3 |
| 3266 | UMHSN00285945 | –9.4 | –9.4 | 3317 | UMHSN00015533 | –9.8 | –9.3 |
| 3267 | UMHSN00291790 | –9.4 | –9.4 | 3318 | UMHSN00031533 | –9.4 | –9.3 |
| 3268 | UMHSN00014872 | –9.7 | –9.3 | 3319 | UMHSN00057704 | –9.4 | –9.3 |
| 3269 | UMHSN00019102 | –9.5 | –9.3 | 3320 | UMHSN00057922 | –9.7 | –9.3 |
| 3270 | UMHSN00026143 | –9.4 | –9.3 | 3321 | UMHSN00174378 | –9.5 | –9.3 |
| 3271 | UMHSN00058008 | –10.3 | –9.3 | 3322 | UMHSN00241659 | –9.4 | –9.3 |
| 3272 | UMHSN00078344 | –9.5 | –9.3 | 3323 | UMHSN00018072 | –9.7 | –9.3 |
| 3273 | UMHSN00104316 | –9.7 | –9.3 | 3324 | UMHSN00026955 | –9.7 | –9.3 |
| 3274 | UMHSN00149211 | –9.4 | –9.3 | 3325 | UMHSN00053178 | –9.5 | –9.3 |
| 3275 | UMHSN00239350 | –10.2 | –9.3 | 3326 | UMHSN00089414 | –9.4 | –9.3 |
| 3276 | UMHSN00288305 | –9.4 | –9.3 | 3327 | UMHSN00095414 | –9.5 | –9.3 |
| 3277 | UMHSN00008858 | –9.7 | –9.3 | 3328 | UMHSN00366258 | –9.7 | –9.3 |
| 3278 | UMHSN00065952 | –10.1 | –9.3 | 3329 | UMHSN00011888 | –9.6 | –9.3 |
| 3279 | UMHSN00080931 | –9.9 | –9.3 | 3330 | UMHSN00013044 | –9.4 | –9.3 |
| 3280 | UMHSN00085038 | –9.8 | –9.3 | 3331 | UMHSN00055097 | –9.5 | –9.3 |
| 3281 | UMHSN00251776 | –9.4 | –9.3 | 3332 | UMHSN00062241 | –9.6 | –9.3 |
| 3282 | UMHSN00026672 | –10.1 | –9.3 | 3333 | UMHSN00080004 | –9.4 | –9.3 |
| 3283 | UMHSN00081189 | –9.9 | –9.3 | 3334 | UMHSN00153349 | –9.4 | –9.3 |
| 3284 | UMHSN00095109 | –9.5 | –9.3 | 3335 | UMHSN00009149 | –10.0 | –9.2 |
| 3285 | UMHSN00150142 | –9.4 | –9.3 | 3336 | UMHSN00009270 | –9.5 | –9.2 |
| 3286 | UMHSN00151643 | –10.1 | –9.3 | 3337 | UMHSN00024651 | –10.1 | –9.2 |
| 3287 | UMHSN00287652 | –9.4 | –9.3 | 3338 | UMHSN00050377 | –9.5 | –9.2 |
| 3288 | UMHSN00012109 | –9.5 | –9.3 | 3339 | UMHSN00094496 | –9.8 | –9.2 |
| 3289 | UMHSN00027752 | –9.4 | –9.3 | 3340 | UMHSN00150259 | –9.5 | –9.2 |
| 3290 | UMHSN00063660 | –9.4 | –9.3 | 3341 | UMHSN00242953 | –9.4 | –9.2 |
| 3291 | UMHSN00083454 | –10.2 | –9.3 | 3342 | UMHSN00010074 | –10.3 | –9.2 |
| 3292 | UMHSN00096893 | –9.5 | –9.3 | 3343 | UMHSN00052438 | –9.5 | –9.2 |
| 3293 | UMHSN00355578 | –9.5 | –9.3 | 3344 | UMHSN00054720 | –9.5 | –9.2 |
| 3294 | UMHSN00016047 | –9.5 | –9.3 | 3345 | UMHSN00064527 | –9.4 | –9.2 |
| 3295 | UMHSN00082505 | –9.4 | –9.3 | 3346 | UMHSN00150359 | –10.2 | –9.2 |
| 3296 | UMHSN00102241 | –9.4 | –9.3 | 3347 | UMHSN00215170 | –10.3 | –9.2 |
| 3297 | UMHSN00151971 | –9.4 | –9.3 | 3348 | UMHSN00027399 | –9.6 | –9.2 |
| 3298 | UMHSN00152110 | –9.5 | –9.3 | 3349 | UMHSN00068014 | –9.6 | –9.2 |
| 3299 | UMHSN00335363 | –9.4 | –9.3 | 3350 | UMHSN00104811 | –10.0 | –9.2 |
| 3300 | UMHSN00016474 | –9.8 | –9.3 | 3351 | UMHSN00153853 | –9.4 | –9.2 |
| 3301 | UMHSN00061967 | –10.1 | –9.3 | 3352 | UMHSN00007086 | –9.8 | –9.2 |
| 3302 | UMHSN00081882 | –9.4 | –9.3 | 3353 | UMHSN00015966 | –9.4 | –9.2 |
| 3303 | UMHSN00082180 | –9.6 | –9.3 | 3354 | UMHSN00018071 | –9.6 | –9.2 |
| 3304 | UMHSN00090964 | –9.4 | –9.3 | 3355 | UMHSN00085560 | –9.4 | –9.2 |
| 3305 | UMHSN00109990 | –10.0 | –9.3 | 3356 | UMHSN00091878 | –9.4 | –9.2 |
| 3306 | UMHSN00270276 | –9.5 | –9.3 | 3357 | UMHSN00100817 | –9.4 | –9.2 |
| 3307 | UMHSN00013141 | –9.5 | –9.3 | 3358 | UMHSN00104917 | –9.4 | –9.2 |
| 3308 | UMHSN00017157 | –9.4 | –9.3 | 3359 | UMHSN00300598 | –9.6 | –9.2 |
| 3309 | UMHSN00071256 | –9.6 | –9.3 | 3360 | UMHSN00004656 | –9.4 | –9.2 |
| 3310 | UMHSN00148437 | –9.6 | –9.3 | 3361 | UMHSN00012099 | –9.6 | –9.2 |
| 3311 | UMHSN00161493 | –9.5 | –9.3 | 3362 | UMHSN00015133 | –9.5 | –9.2 |
| 3312 | UMHSN00261447 | –9.4 | –9.3 | 3363 | UMHSN00057373 | –10.3 | –9.2 |
| 3313 | UMHSN00269812 | –9.6 | –9.3 | 3364 | UMHSN00065816 | –9.8 | –9.2 |
| 3314 | UMHSN00427289 | –9.6 | –9.3 | 3365 | UMHSN00078247 | –10.2 | –9.2 |
| 3315 | UMHSN00008565 | –9.4 | –9.3 | 3366 | UMHSN00084664 | –9.8 | –9.2 |

| No. | SuperNatural II Code | Docking Score (kcal/mol) | | No. | SuperNatural II Code | Docking Score (kcal/mol) | |
| --- | --- | --- | --- | --- | --- | --- | --- |
|  |  | Std.^b^ | Mod.^c^ |  |  | Std.^b^ | Mod.^c^ |
| 3367 | UMHSN00053384 | –9.5 | –9.2 | 3418 | UMHSN00080958 | –9.5 | –9.1 |
| 3368 | UMHSN00061623 | –9.5 | –9.2 | 3419 | UMHSN00112272 | –9.4 | –9.1 |
| 3369 | UMHSN00078618 | –9.9 | –9.2 | 3420 | UMHSN00150291 | –9.7 | –9.1 |
| 3370 | UMHSN00342908 | –9.6 | –9.2 | 3421 | UMHSN00161152 | –9.5 | –9.1 |
| 3371 | UMHSN00009252 | –9.5 | –9.2 | 3422 | UMHSN00007083 | –9.8 | –9.1 |
| 3372 | UMHSN00054120 | –9.9 | –9.2 | 3423 | UMHSN00009446 | –9.4 | –9.1 |
| 3373 | UMHSN00073691 | –10.1 | –9.2 | 3424 | UMHSN00061939 | –10.3 | –9.1 |
| 3374 | UMHSN00063089 | –9.4 | –9.2 | 3425 | UMHSN00062178 | –10.5 | –9.1 |
| 3375 | UMHSN00090917 | –9.5 | –9.2 | 3426 | UMHSN00073828 | –9.6 | –9.1 |
| 3376 | UMHSN00150443 | –9.7 | –9.2 | 3427 | UMHSN00095755 | –9.5 | –9.1 |
| 3377 | UMHSN00168421 | –9.6 | –9.2 | 3428 | UMHSN00103910 | –9.8 | –9.1 |
| 3378 | UMHSN00261623 | –9.5 | –9.2 | 3429 | UMHSN00154614 | –9.5 | –9.1 |
| 3379 | UMHSN00031088 | –9.6 | –9.2 | 3430 | UMHSN00264808 | –9.4 | –9.1 |
| 3380 | UMHSN00031974 | –9.6 | –9.2 | 3431 | UMHSN00028339 | –9.7 | –9.1 |
| 3381 | UMHSN00058755 | –9.9 | –9.2 | 3432 | UMHSN00090215 | –9.5 | –9.1 |
| 3382 | UMHSN00075116 | –9.4 | –9.2 | 3433 | UMHSN00057100 | –9.7 | –9.0 |
| 3383 | UMHSN00081975 | –9.4 | –9.2 | 3434 | UMHSN00077385 | –9.4 | –9.0 |
| 3384 | UMHSN00082282 | –9.4 | –9.2 | 3435 | UMHSN00080606 | –9.4 | –9.0 |
| 3385 | UMHSN00091915 | –9.5 | –9.2 | 3436 | UMHSN00099102 | –10.0 | –9.0 |
| 3386 | UMHSN00343628 | –9.6 | –9.2 | 3437 | UMHSN00113622 | –9.4 | –9.0 |
| 3387 | UMHSN00052353 | –9.4 | –9.2 | 3438 | UMHSN00154642 | –9.5 | –9.0 |
| 3388 | UMHSN00059990 | –10.0 | –9.2 | 3439 | UMHSN00078920 | –10.1 | –9.0 |
| 3389 | UMHSN00076701 | –9.5 | –9.2 | 3440 | UMHSN00081615 | –9.9 | –9.0 |
| 3390 | UMHSN00172106 | –9.6 | –9.2 | 3441 | UMHSN00014320 | –10.0 | –9.0 |
| 3391 | UMHSN00010448 | –9.9 | –9.1 | 3442 | UMHSN00087356 | –9.7 | –9.0 |
| 3392 | UMHSN00004979 | –9.5 | –9.1 | 3443 | UMHSN00153859 | –9.4 | –9.0 |
| 3393 | UMHSN00005077 | –9.5 | –9.1 | 3444 | UMHSN00351955 | –9.7 | –9.0 |
| 3394 | UMHSN00005271 | –9.6 | –9.1 | 3445 | UMHSN00374875 | –9.4 | –9.0 |
| 3395 | UMHSN00025820 | –10.1 | –9.1 | 3446 | UMHSN00078316 | –9.4 | –9.0 |
| 3396 | UMHSN00057175 | –9.6 | –9.1 | 3447 | UMHSN00171973 | –9.6 | –9.0 |
| 3397 | UMHSN00090919 | –9.7 | –9.1 | 3448 | UMHSN00057919 | –9.4 | –9.0 |
| 3398 | UMHSN00009984 | –9.7 | –9.1 | 3449 | UMHSN00073909 | –9.7 | –9.0 |
| 3399 | UMHSN00014543 | –9.6 | –9.1 | 3450 | UMHSN00015136 | –9.9 | –9.0 |
| 3400 | UMHSN00014835 | –9.9 | –9.1 | 3451 | UMHSN00021596 | –10.2 | –9.0 |
| 3401 | UMHSN00062695 | –9.5 | –9.1 | 3452 | UMHSN00027894 | –9.6 | –9.0 |
| 3402 | UMHSN00145254 | –10.1 | –9.1 | 3453 | UMHSN00052691 | –9.6 | –9.0 |
| 3403 | UMHSN00012184 | –9.4 | –9.1 | 3454 | UMHSN00154286 | –10.5 | –9.0 |
| 3404 | UMHSN00112246 | –9.4 | –9.1 | 3455 | UMHSN00013031 | –9.6 | –9.0 |
| 3405 | UMHSN00154284 | –9.6 | –9.1 | 3456 | UMHSN00054118 | –9.5 | –9.0 |
| 3406 | UMHSN00053520 | –9.7 | –9.1 | 3457 | UMHSN00078597 | –9.8 | –9.0 |
| 3407 | UMHSN00091812 | –9.5 | –9.1 | 3458 | UMHSN00080981 | –9.5 | –9.0 |
| 3408 | UMHSN00427146 | –10.4 | –9.1 | 3459 | UMHSN00084591 | –9.4 | –9.0 |
| 3409 | UMHSN00004110 | –9.7 | –9.1 | 3460 | UMHSN00270474 | –9.5 | –9.0 |
| 3410 | UMHSN00076369 | –9.4 | –9.1 | 3461 | UMHSN00403356 | –9.5 | –9.0 |
| 3411 | UMHSN00054753 | –9.6 | –9.1 | 3462 | UMHSN00001643 | –9.6 | –8.9 |
| 3412 | UMHSN00072145 | –9.5 | –9.1 | 3463 | UMHSN00082823 | –9.6 | –8.9 |
| 3413 | UMHSN00080048 | –9.4 | –9.1 | 3464 | UMHSN00090781 | –9.7 | –8.9 |
| 3414 | UMHSN00083206 | –9.8 | –9.1 | 3465 | UMHSN00115639 | –10.3 | –8.9 |
| 3415 | UMHSN00098572 | –9.5 | –9.1 | 3466 | UMHSN00056844 | –10.2 | –8.9 |
| 3416 | UMHSN00170575 | –9.5 | –9.1 | 3467 | UMHSN00058992 | –9.5 | –8.9 |
| 3417 | UMHSN00031969 | –9.4 | –9.1 | 3468 | UMHSN00109742 | –9.9 | –8.9 |

| No. | SuperNatural II Code | Docking Score (kcal/mol) | | No. | SuperNatural II Code | Docking Score (kcal/mol) | |
| --- | --- | --- | --- | --- | --- | --- | --- |
|  |  | Std.^b^ | Mod.^c^ |  |  | Std.^b^ | Mod.^c^ |
| 3469 | UMHSN00154565 | –9.6 | –8.9 | 3520 | UMHSN00428156 | –9.8 | –8.8 |
| 3470 | UMHSN00072350 | –9.6 | –8.9 | 3521 | UMHSN00081281 | –9.6 | –8.8 |
| 3471 | UMHSN00427088 | –9.5 | –8.9 | 3522 | UMHSN00103882 | –9.6 | –8.8 |
| 3472 | UMHSN00072740 | –9.5 | –8.9 | 3523 | UMHSN00005000 | –9.7 | –8.8 |
| 3473 | UMHSN00248091 | –9.4 | –8.9 | 3524 | UMHSN00007077 | –10.3 | –8.8 |
| 3474 | UMHSN00018011 | –9.5 | –8.9 | 3525 | UMHSN00057642 | –9.4 | –8.8 |
| 3475 | UMHSN00077375 | –10.9 | –8.9 | 3526 | UMHSN00080093 | –9.6 | –8.8 |
| 3476 | UMHSN00010075 | –10.0 | –8.9 | 3527 | UMHSN00127637 | –9.4 | –8.8 |
| 3477 | UMHSN00028280 | –9.4 | –8.9 | 3528 | UMHSN00014372 | –9.6 | –8.8 |
| 3478 | UMHSN00076753 | –9.6 | –8.9 | 3529 | UMHSN00078420 | –9.4 | –8.8 |
| 3479 | UMHSN00060980 | –9.5 | –8.9 | 3530 | UMHSN00328968 | –9.7 | –8.8 |
| 3480 | UMHSN00082627 | –9.6 | –8.9 | 3531 | UMHSN00362129 | –9.7 | –8.8 |
| 3481 | UMHSN00319611 | –9.4 | –8.9 | 3532 | UMHSN00050376 | –9.4 | –8.7 |
| 3482 | UMHSN00109433 | –10.3 | –8.9 | 3533 | UMHSN00022053 | –9.8 | –8.7 |
| 3483 | UMHSN00307769 | –9.9 | –8.9 | 3534 | UMHSN00052555 | –9.4 | –8.7 |
| 3484 | UMHSN00013955 | –9.4 | –8.9 | 3535 | UMHSN00060857 | –10.4 | –8.7 |
| 3485 | UMHSN00026805 | –9.4 | –8.9 | 3536 | UMHSN00062793 | –10.7 | –8.7 |
| 3486 | UMHSN00102069 | –9.4 | –8.9 | 3537 | UMHSN00076053 | –9.4 | –8.7 |
| 3487 | UMHSN00153721 | –10.0 | –8.9 | 3538 | UMHSN00089789 | –9.9 | –8.7 |
| 3488 | UMHSN00008116 | –9.4 | –8.9 | 3539 | UMHSN00300309 | –9.4 | –8.7 |
| 3489 | UMHSN00012383 | –9.8 | –8.9 | 3540 | UMHSN00009995 | –10.5 | –8.7 |
| 3490 | UMHSN00018114 | –9.6 | –8.9 | 3541 | UMHSN00022496 | –9.5 | –8.7 |
| 3491 | UMHSN00057607 | –9.4 | –8.9 | 3542 | UMHSN00050378 | –9.6 | –8.7 |
| 3492 | UMHSN00077618 | –9.9 | –8.9 | 3543 | UMHSN00053020 | –9.4 | –8.7 |
| 3493 | UMHSN00112302 | –9.7 | –8.9 | 3544 | UMHSN00098590 | –9.9 | –8.7 |
| 3494 | UMHSN00021614 | –9.4 | –8.8 | 3545 | UMHSN00427278 | –9.5 | –8.7 |
| 3495 | UMHSN00058539 | –9.7 | –8.8 | 3546 | UMHSN00089204 | –9.5 | –8.7 |
| 3496 | UMHSN00168441 | –9.4 | –8.8 | 3547 | UMHSN00081007 | –9.8 | –8.7 |
| 3497 | UMHSN00236043 | –9.6 | –8.8 | 3548 | UMHSN00081956 | –9.9 | –8.7 |
| 3498 | UMHSN00008213 | –9.4 | –8.8 | 3549 | UMHSN00085262 | –9.5 | –8.7 |
| 3499 | UMHSN00008818 | –9.4 | –8.8 | 3550 | UMHSN00253333 | –9.5 | –8.7 |
| 3500 | UMHSN00005203 | –9.7 | –8.8 | 3551 | UMHSN00012832 | –9.5 | –8.7 |
| 3501 | UMHSN00365270 | –9.6 | –8.8 | 3552 | UMHSN00003801 | –9.5 | –8.7 |
| 3502 | UMHSN00070960 | –9.9 | –8.8 | 3553 | UMHSN00014014 | –9.4 | –8.7 |
| 3503 | UMHSN00010864 | –9.4 | –8.8 | 3554 | UMHSN00062019 | –9.5 | –8.7 |
| 3504 | UMHSN00024602 | –9.5 | –8.8 | 3555 | UMHSN00090711 | –9.4 | –8.7 |
| 3505 | UMHSN00061885 | –9.9 | –8.8 | 3556 | UMHSN00008164 | –9.7 | –8.7 |
| 3506 | UMHSN00072313 | –9.5 | –8.8 | 3557 | UMHSN00018159 | –9.5 | –8.7 |
| 3507 | UMHSN00077246 | –10.0 | –8.8 | 3558 | UMHSN00024046 | –9.9 | –8.7 |
| 3508 | UMHSN00112163 | –9.4 | –8.8 | 3559 | UMHSN00009791 | –9.8 | –8.6 |
| 3509 | UMHSN00302153 | –9.4 | –8.8 | 3560 | UMHSN00154571 | –10.7 | –8.6 |
| 3510 | UMHSN00007142 | –10.2 | –8.8 | 3561 | UMHSN00018630 | –9.6 | –8.6 |
| 3511 | UMHSN00008093 | –10.3 | –8.8 | 3562 | UMHSN00056888 | –10.1 | –8.6 |
| 3512 | UMHSN00013074 | –10.0 | –8.8 | 3563 | UMHSN00057857 | –9.5 | –8.6 |
| 3513 | UMHSN00014780 | –9.6 | –8.8 | 3564 | UMHSN00100334 | –9.8 | –8.6 |
| 3514 | UMHSN00080163 | –9.5 | –8.8 | 3565 | UMHSN00154044 | –9.6 | –8.6 |
| 3515 | UMHSN00082596 | –10.5 | –8.8 | 3566 | UMHSN00018210 | –9.6 | –8.6 |
| 3516 | UMHSN00015697 | –9.6 | –8.8 | 3567 | UMHSN00059333 | –9.8 | –8.6 |
| 3517 | UMHSN00078359 | –9.4 | –8.8 | 3568 | UMHSN00034910 | –9.4 | –8.6 |
| 3518 | UMHSN00081721 | –10.2 | –8.8 | 3569 | UMHSN00007424 | –9.8 | –8.6 |
| 3519 | UMHSN00279512 | –9.7 | –8.8 | 3570 | UMHSN00017994 | –9.5 | –8.6 |

| No. | SuperNatural II Code | Docking Score (kcal/mol) | | No. | SuperNatural II Code | Docking Score (kcal/mol) | |
| --- | --- | --- | --- | --- | --- | --- | --- |
|  |  | Std.^b^ | Mod.^c^ |  |  | Std.^b^ | Mod.^c^ |
| 3571 | UMHSN00053441 | –9.6 | –8.6 | 3622 | UMHSN00427476 | –9.8 | –8.3 |
| 3572 | UMHSN00154516 | –9.6 | –8.6 | 3623 | UMHSN00013423 | –9.4 | –8.3 |
| 3573 | UMHSN00170302 | –10.2 | –8.6 | 3624 | UMHSN00016189 | –9.6 | –8.3 |
| 3574 | UMHSN00057608 | –10.0 | –8.6 | 3625 | UMHSN00019278 | –9.5 | –8.3 |
| 3575 | UMHSN00078954 | –10.2 | –8.6 | 3626 | UMHSN00053762 | –9.4 | –8.3 |
| 3576 | UMHSN00014752 | –9.9 | –8.6 | 3627 | UMHSN00063387 | –9.7 | –8.3 |
| 3577 | UMHSN00355363 | –9.8 | –8.6 | 3628 | UMHSN00079092 | –10.6 | –8.3 |
| 3578 | UMHSN00059961 | –9.6 | –8.6 | 3629 | UMHSN00010610 | –10.0 | –8.3 |
| 3579 | UMHSN00060371 | –10.5 | –8.6 | 3630 | UMHSN00090163 | –9.4 | –8.3 |
| 3580 | UMHSN00079514 | –9.4 | –8.6 | 3631 | UMHSN00151603 | –9.9 | –8.3 |
| 3581 | UMHSN00013001 | –9.5 | –8.6 | 3632 | UMHSN00007082 | –9.6 | –8.3 |
| 3582 | UMHSN00110171 | –9.6 | –8.6 | 3633 | UMHSN00059366 | –9.9 | –8.3 |
| 3583 | UMHSN00005277 | –9.6 | –8.5 | 3634 | UMHSN00079559 | –9.6 | –8.3 |
| 3584 | UMHSN00077094 | –9.4 | –8.5 | 3635 | UMHSN00089694 | –9.7 | –8.3 |
| 3585 | UMHSN00316795 | –9.4 | –8.5 | 3636 | UMHSN00015583 | –10.4 | –8.3 |
| 3586 | UMHSN00063109 | –10.1 | –8.5 | 3637 | UMHSN00018211 | –9.8 | –8.3 |
| 3587 | UMHSN00012826 | –9.5 | –8.5 | 3638 | UMHSN00082592 | –10.0 | –8.3 |
| 3588 | UMHSN00018370 | –9.4 | –8.5 | 3639 | UMHSN00112138 | –9.4 | –8.3 |
| 3589 | UMHSN00019009 | –9.7 | –8.5 | 3640 | UMHSN00006428 | –10.4 | –8.2 |
| 3590 | UMHSN00024812 | –9.5 | –8.5 | 3641 | UMHSN00013003 | –9.9 | –8.2 |
| 3591 | UMHSN00035477 | –9.5 | –8.5 | 3642 | UMHSN00061066 | –10.3 | –8.2 |
| 3592 | UMHSN00060747 | –9.4 | –8.5 | 3643 | UMHSN00084627 | –9.4 | –8.2 |
| 3593 | UMHSN00105277 | –9.5 | –8.5 | 3644 | UMHSN00170111 | –9.6 | –8.2 |
| 3594 | UMHSN00023783 | –9.6 | –8.5 | 3645 | UMHSN00024765 | –9.5 | –8.2 |
| 3595 | UMHSN00052861 | –10.2 | –8.5 | 3646 | UMHSN00058524 | –10.2 | –8.2 |
| 3596 | UMHSN00015049 | –9.4 | –8.5 | 3647 | UMHSN00082681 | –10.4 | –8.2 |
| 3597 | UMHSN00016395 | –9.5 | –8.5 | 3648 | UMHSN00097855 | –9.4 | –8.2 |
| 3598 | UMHSN00023798 | –9.9 | –8.5 | 3649 | UMHSN00022079 | –10.1 | –8.2 |
| 3599 | UMHSN00027945 | –9.6 | –8.5 | 3650 | UMHSN00293060 | –10.0 | –8.2 |
| 3600 | UMHSN00063270 | –10.3 | –8.5 | 3651 | UMHSN00005458 | –9.5 | –8.2 |
| 3601 | UMHSN00024770 | –9.5 | –8.4 | 3652 | UMHSN00057893 | –9.5 | –8.2 |
| 3602 | UMHSN00076190 | –9.4 | –8.4 | 3653 | UMHSN00022073 | –9.8 | –8.2 |
| 3603 | UMHSN00083138 | –9.5 | –8.4 | 3654 | UMHSN00034913 | –9.4 | –8.2 |
| 3604 | UMHSN00058386 | –9.9 | –8.4 | 3655 | UMHSN00059003 | –10.2 | –8.2 |
| 3605 | UMHSN00261393 | –9.4 | –8.4 | 3656 | UMHSN00070417 | –9.8 | –8.2 |
| 3606 | UMHSN00022144 | –9.4 | –8.4 | 3657 | UMHSN00026504 | –9.7 | –8.1 |
| 3607 | UMHSN00112144 | –9.5 | –8.4 | 3658 | UMHSN00081182 | –9.4 | –8.1 |
| 3608 | UMHSN00016390 | –9.7 | –8.4 | 3659 | UMHSN00106598 | –9.7 | –8.1 |
| 3609 | UMHSN00113414 | –10.0 | –8.4 | 3660 | UMHSN00024786 | –9.9 | –8.1 |
| 3610 | UMHSN00027652 | –9.4 | –8.4 | 3661 | UMHSN00080046 | –9.7 | –8.1 |
| 3611 | UMHSN00032122 | –10.6 | –8.4 | 3662 | UMHSN00098596 | –9.4 | –8.1 |
| 3612 | UMHSN00016880 | –9.4 | –8.4 | 3663 | UMHSN00009938 | –10.2 | –8.1 |
| 3613 | UMHSN00081761 | –9.8 | –8.4 | 3664 | UMHSN00077393 | –10.0 | –8.1 |
| 3614 | UMHSN00098616 | –9.4 | –8.4 | 3665 | UMHSN00007454 | –9.4 | –8.1 |
| 3615 | UMHSN00353715 | –9.5 | –8.4 | 3666 | UMHSN00017240 | –9.4 | –8.1 |
| 3616 | UMHSN00057149 | –9.7 | –8.3 | 3667 | UMHSN00173399 | –10.0 | –8.1 |
| 3617 | UMHSN00005286 | –9.6 | –8.3 | 3668 | UMHSN00022063 | –9.5 | –8.1 |
| 3618 | UMHSN00025118 | –9.5 | –8.3 | 3669 | UMHSN00061789 | –9.6 | –8.1 |
| 3619 | UMHSN00031527 | –9.7 | –8.3 | 3670 | UMHSN00081441 | –10.0 | –8.1 |
| 3620 | UMHSN00081988 | –10.0 | –8.3 | 3671 | UMHSN00173787 | –9.7 | –8.1 |
| 3621 | UMHSN00006744 | –9.7 | –8.3 | 3672 | UMHSN00249385 | –9.6 | –8.1 |

| No. | SuperNatural II Code | Docking Score (kcal/mol) | | No. | SuperNatural II Code | Docking Score (kcal/mol) | |
| --- | --- | --- | --- | --- | --- | --- | --- |
|  |  | Std.^b^ | Mod.^c^ |  |  | Std.^b^ | Mod.^c^ |
| 3673 | UMHSN00097238 | –9.8 | –8.1 | 3713 | UMHSN00027211 | –9.6 | –7.7 |
| 3674 | UMHSN00108961 | –9.9 | –8.1 | 3714 | UMHSN00024703 | –9.5 | –7.7 |
| 3675 | UMHSN00259338 | –9.4 | –8.1 | 3715 | UMHSN00320484 | –9.6 | –7.7 |
| 3676 | UMHSN00010614 | –9.9 | –8.1 | 3716 | UMHSN00094019 | –9.6 | –7.7 |
| 3677 | UMHSN00244684 | –9.5 | –8.1 | 3717 | UMHSN00056395 | –9.7 | –7.6 |
| 3678 | UMHSN00083866 | –9.5 | –8.0 | 3718 | UMHSN00076491 | –9.4 | –7.6 |
| 3679 | UMHSN00018439 | –9.8 | –8.0 | 3719 | UMHSN00080761 | –9.5 | –7.6 |
| 3680 | UMHSN00076603 | –10.2 | –8.0 | 3720 | UMHSN00014651 | –9.7 | –7.6 |
| 3681 | UMHSN00017981 | –9.6 | –8.0 | 3721 | UMHSN00016460 | –9.8 | –7.6 |
| 3682 | UMHSN00073738 | –9.5 | –8.0 | 3722 | UMHSN00021681 | –9.4 | –7.6 |
| 3683 | UMHSN00005072 | –9.6 | –8.0 | 3723 | UMHSN00027232 | –9.6 | –7.6 |
| 3684 | UMHSN00057444 | –10.1 | –8.0 | 3724 | UMHSN00082917 | –9.8 | –7.6 |
| 3685 | UMHSN00097248 | –9.9 | –8.0 | 3725 | UMHSN00083987 | –9.7 | –7.5 |
| 3686 | UMHSN00376421 | –10.2 | –8.0 | 3726 | UMHSN00078981 | –9.4 | –7.5 |
| 3687 | UMHSN00018423 | –9.4 | –8.0 | 3727 | UMHSN00080772 | –9.7 | –7.5 |
| 3688 | UMHSN00084914 | –9.5 | –7.9 | 3728 | UMHSN00032177 | –9.8 | –7.4 |
| 3689 | UMHSN00016457 | –9.6 | –7.9 | 3729 | UMHSN00016473 | –9.7 | –7.4 |
| 3690 | UMHSN00081401 | –9.4 | –7.9 | 3730 | UMHSN00028188 | –9.8 | –7.4 |
| 3691 | UMHSN00101472 | –9.9 | –7.9 | 3731 | UMHSN00427441 | –9.7 | –7.4 |
| 3692 | UMHSN00016662 | –9.4 | –7.9 | 3732 | UMHSN00362642 | –9.4 | –7.3 |
| 3693 | UMHSN00427378 | –11.4 | –7.9 | 3733 | UMHSN00063129 | –9.4 | –7.3 |
| 3694 | UMHSN00008070 | –9.7 | –7.9 | 3734 | UMHSN00013143 | –10.0 | –7.2 |
| 3695 | UMHSN00297689 | –10.0 | –7.9 | 3735 | UMHSN00361725 | –9.5 | –7.2 |
| 3696 | UMHSN00053516 | –9.7 | –7.9 | 3736 | UMHSN00267551 | –9.4 | –7.2 |
| 3697 | UMHSN00083059 | –9.6 | –7.8 | 3737 | UMHSN00012247 | –9.4 | –7.2 |
| 3698 | UMHSN00085063 | –9.5 | –7.8 | 3738 | UMHSN00048830 | –10.4 | –7.2 |
| 3699 | UMHSN00333556 | –9.4 | –7.8 | 3739 | UMHSN00063558 | –9.4 | –7.2 |
| 3700 | UMHSN00014662 | –9.7 | –7.8 | 3740 | UMHSN00013054 | –9.5 | –7.2 |
| 3701 | UMHSN00024695 | –9.5 | –7.8 | 3741 | UMHSN00057111 | –9.9 | –7.2 |
| 3702 | UMHSN00017978 | –9.7 | –7.8 | 3742 | UMHSN00059509 | –10.0 | –7.2 |
| 3703 | UMHSN00081010 | –10.1 | –7.8 | 3743 | UMHSN00013132 | –10.0 | –7.1 |
| 3704 | UMHSN00080504 | –9.9 | –7.8 | 3744 | UMHSN00089404 | –9.6 | –7.0 |
| 3705 | UMHSN00015693 | –9.6 | –7.8 | 3745 | UMHSN00016705 | –9.5 | –6.9 |
| 3706 | UMHSN00077374 | –9.4 | –7.8 | 3746 | UMHSN00027224 | –9.5 | –6.9 |
| 3707 | UMHSN00112675 | –9.7 | –7.8 | 3747 | UMHSN00154172 | –9.9 | –6.7 |
| 3708 | UMHSN00151598 | –9.5 | –7.8 | 3748 | UMHSN00387424 | –9.5 | –6.6 |
| 3709 | UMHSN00299166 | –9.5 | –7.8 | 3749 | UMHSN00017969 | –9.5 | –6.1 |
| 3710 | UMHSN00018014 | –10.7 | –7.8 | 3750 | UMHSN00105242 | –9.4 | –6.0 |
| 3711 | UMHSN00026344 | –9.6 | –7.8 | 3751 | UMHSN00177500 | –9.5 | –5.7 |
| 3712 | UMHSN00081353 | –9.8 | –7.8 |  |  |  |  |

^a^ Data ranked based on the moderate docking calculations.

^b^ Std. refers to standard docking calculations.

^c^ Mod. refers to moderate docking calculations.

^d^ Exp. refers to expensive docking calculations.
