# Supplementary material for: SuperNatural inhibitors to reverse multidrug resistance emerged by ABCB1 transporter: Database mining, lipid-mediated molecular dynamics, and pharmacokinetics study
Source: PLoS One. 2023 Jul 26;18(7):e0288919. doi: 10.1371/journal.pone.0288919 (PMC10370898; doi:10.1371/journal.pone.0288919)
Supplement: S2 Table — (DOCX) [file pone.0288919.s003.docx]

### S2 Table. Estimated standard, moderate, and expensive docking scores (in kcal/mol) for the top 376 compounds and ZQU within the ABCB1 binding pocket. ^a^

| No. | SuperNatural II Code | Docking Score (kcal/mol) | | |
| --- | --- | --- | --- | --- |
|  |  | Std.^b^ | Mod.^c^ | Exp.^d^ |
|  | **ZQU** | –8.4 | –8.2 | –8.3 |
| 1 | UMHSN00009999 | –12.5 | –13.1 | –12.6 |
| 2 | UMHSN00054807 | –12.5 | –13.1 | –12.6 |
| 3 | UMHSN00081043 | –12.5 | –12.6 | –12.5 |
| 4 | UMHSN00097206 | –12.5 | –11.5 | –12.5 |
| 5 | UMHSN00062899 | –12.1 | –12.4 | –12.4 |
| 6 | UMHSN00066079 | –12.4 | –12.4 | –12.4 |
| 7 | UMHSN00008763 | –10.4 | –11.9 | –12.1 |
| 8 | UMHSN00302140 | –12.1 | –12.1 | –12.1 |
| 9 | UMHSN00081081 | –10.4 | –10.6 | –12.1 |
| 10 | UMHSN00080936 | –11.9 | –11.9 | –11.9 |
| 11 | UMHSN00011720 | –12.7 | –12.8 | –11.9 |
| 12 | UMHSN00059546 | –10.8 | –11.6 | –11.8 |
| 13 | UMHSN00080939 | –11.8 | –11.8 | –11.8 |
| 14 | UMHSN00081067 | –11.8 | –11.8 | –11.8 |
| 15 | UMHSN00058310 | –10.5 | –11.8 | –11.8 |
| 16 | UMHSN00337215 | –11.8 | –11.8 | –11.8 |
| 17 | UMHSN00380932 | –11.6 | –11.8 | –11.8 |
| 18 | UMHSN00089274 | –11.5 | –11.8 | –11.8 |
| 19 | UMHSN00317150 | –11.8 | –11.8 | –11.8 |
| 20 | UMHSN00081079 | –11.7 | –11.7 | –11.7 |
| 21 | UMHSN00004668 | –11.0 | –11.6 | –11.6 |
| 22 | UMHSN00008381 | –10.0 | –11.5 | –11.6 |
| 23 | UMHSN00009954 | –11.4 | –11.5 | –11.6 |
| 24 | UMHSN00360652 | –11.5 | –11.5 | –11.5 |
| 25 | UMHSN00007945 | –10.2 | –11.5 | –11.5 |
| 26 | UMHSN00260998 | –10.9 | –11.5 | –11.5 |
| 27 | UMHSN00265972 | –11.5 | –11.5 | –11.5 |
| 28 | UMHSN00079222 | –11.2 | –11.4 | –11.5 |
| 29 | UMHSN00054684 | –11.4 | –11.5 | –11.5 |
| 30 | UMHSN00009897 | –11.2 | –11.8 | –11.5 |
| 31 | UMHSN00084553 | –11.1 | –11.4 | –11.5 |
| 32 | UMHSN00010807 | –11.3 | –11.4 | –11.4 |
| 33 | UMHSN00062975 | –11.5 | –11.6 | –11.4 |
| 34 | UMHSN00249525 | –11.4 | –11.4 | –11.4 |
| 35 | UMHSN00139755 | –9.7 | –11.4 | –11.4 |
| 36 | UMHSN00377659 | –11.4 | –11.4 | –11.4 |
| 37 | UMHSN00050809 | –9.9 | –11.6 | –11.3 |
| 38 | UMHSN00427183 | –10.9 | –11.4 | –11.2 |
| 39 | UMHSN00079819 | –10.3 | –11.0 | –11.1 |
| 40 | UMHSN00010270 | –9.4 | –11.0 | –11.0 |
| 41 | UMHSN00014785 | –11.0 | –11.0 | –11.0 |
| 42 | UMHSN00004210 | –11.0 | –11.0 | –11.0 |
| 43 | UMHSN00291292 | –11.0 | –11.0 | –11.0 |
| 44 | UMHSN00007078 | –11.0 | –11.2 | –11.0 |
| 45 | UMHSN00008336 | –11.2 | –11.2 | –11.0 |
| 46 | UMHSN00008385 | –11.1 | –11.3 | –11.0 |

| No. | SuperNatural II Code | Docking Score (kcal/mol) | | |
| --- | --- | --- | --- | --- |
|  |  | Std.^b^ | Mod.^c^ | Exp.^d^ |
| 47 | UMHSN00008510 | –11.2 | –11.2 | –11.0 |
| 48 | UMHSN00008771 | –10.5 | –11.0 | –11.0 |
| 49 | UMHSN00009953 | –11.2 | –11.3 | –11.0 |
| 50 | UMHSN00009957 | –10.4 | –11.1 | –11.0 |
| 51 | UMHSN00048096 | –10.9 | –11.1 | –11.0 |
| 52 | UMHSN00053680 | –9.5 | –11.0 | –11.0 |
| 53 | UMHSN00059415 | –10.8 | –11.1 | –11.0 |
| 54 | UMHSN00080511 | –11.1 | –11.2 | –11.0 |
| 55 | UMHSN00080908 | –9.7 | –11.3 | –11.0 |
| 56 | UMHSN00081514 | –10.7 | –11.1 | –11.0 |
| 57 | UMHSN00081749 | –11.3 | –11.3 | –11.0 |
| 58 | UMHSN00084545 | –10.8 | –11.1 | –11.0 |
| 59 | UMHSN00089524 | –9.5 | –11.2 | –11.0 |
| 60 | UMHSN00089681 | –10.3 | –11.3 | –11.0 |
| 61 | UMHSN00089689 | –10.8 | –11.0 | –11.0 |
| 62 | UMHSN00133181 | –10.5 | –11.0 | –11.0 |
| 63 | UMHSN00168544 | –11.0 | –11.0 | –11.0 |
| 64 | UMHSN00265761 | –11.1 | –11.2 | –11.0 |
| 65 | UMHSN00286721 | –10.0 | –11.0 | –11.0 |
| 66 | UMHSN00321430 | –11.0 | –11.1 | –11.0 |
| 67 | UMHSN00335397 | –10.9 | –11.1 | –11.0 |
| 68 | UMHSN00342900 | –11.2 | –11.3 | –11.0 |
| 69 | UMHSN00345449 | –11.2 | –11.2 | –11.0 |
| 70 | UMHSN00378848 | –11.4 | –11.4 | –11.0 |
| 71 | UMHSN00006367 | –9.4 | –11.0 | –11.0 |
| 72 | UMHSN00014817 | –10.8 | –11.0 | –11.0 |
| 73 | UMHSN00008774 | –11.0 | –11.0 | –11.0 |
| 74 | UMHSN00295529 | –10.9 | –11.0 | –11.0 |
| 75 | UMHSN00011707 | –10.9 | –11.0 | –11.0 |
| 76 | UMHSN00101579 | –11.0 | –11.0 | –11.0 |
| 77 | UMHSN00148374 | –10.3 | –10.9 | –11.0 |
| 78 | UMHSN00055285 | –10.8 | –10.9 | –11.0 |
| 79 | UMHSN00008340 | –10.9 | –10.9 | –10.9 |
| 80 | UMHSN00009729 | –11.0 | –11.0 | –10.9 |
| 81 | UMHSN00061743 | –10.1 | –10.9 | –10.9 |
| 82 | UMHSN00127113 | –9.6 | –10.9 | –10.9 |
| 83 | UMHSN00082123 | –11.0 | –11.0 | –10.9 |
| 84 | UMHSN00150166 | –10.8 | –10.9 | –10.9 |
| 85 | UMHSN00013456 | –10.5 | –11.0 | –10.9 |
| 86 | UMHSN00081767 | –10.9 | –10.9 | –10.9 |
| 87 | UMHSN00004196 | –10.9 | –10.9 | –10.9 |
| 88 | UMHSN00005185 | –11.3 | –11.3 | –10.9 |
| 89 | UMHSN00008793 | –10.7 | –10.9 | –10.9 |
| 90 | UMHSN00009721 | –11.0 | –11.0 | –10.9 |
| 91 | UMHSN00009733 | –10.5 | –11.2 | –10.9 |
| 92 | UMHSN00010794 | –11.2 | –11.2 | –10.9 |
| 93 | UMHSN00011710 | –11.0 | –11.0 | –10.9 |
| 94 | UMHSN00018080 | –10.0 | –10.8 | –10.9 |
| 95 | UMHSN00052580 | –9.5 | –10.9 | –10.9 |
| 96 | UMHSN00059308 | –10.8 | –10.8 | –10.9 |
| 97 | UMHSN00060131 | –10.7 | –11.2 | –10.9 |
| 98 | UMHSN00080836 | –10.9 | –11.1 | –10.9 |
| 99 | UMHSN00080951 | –9.8 | –11.2 | –10.9 |
| 100 | UMHSN00081530 | –10.7 | –11.3 | –10.9 |
| 101 | UMHSN00084547 | –10.9 | –11.3 | –10.9 |
| 102 | UMHSN00091326 | –10.9 | –10.9 | –10.9 |
| 103 | UMHSN00097225 | –11.1 | –11.1 | –10.9 |

| No. | SuperNatural II Code | Docking Score (kcal/mol) | | |
| --- | --- | --- | --- | --- |
|  |  | Std.^b^ | Mod.^c^ | Exp.^d^ |
| 104 | UMHSN00151994 | –10.1 | –10.9 | –10.9 |
| 105 | UMHSN00153504 | –10.8 | –10.9 | –10.9 |
| 106 | UMHSN00234079 | –9.8 | –11.1 | –10.9 |
| 107 | UMHSN00274123 | –11.0 | –11.1 | –10.9 |
| 108 | UMHSN00294698 | –10.8 | –10.9 | –10.9 |
| 109 | UMHSN00005497 | –10.7 | –10.9 | –10.9 |
| 110 | UMHSN00009839 | –10.9 | –10.9 | –10.9 |
| 111 | UMHSN00049070 | –10.7 | –10.8 | –10.9 |
| 112 | UMHSN00081583 | –10.7 | –11.0 | –10.9 |
| 113 | UMHSN00057710 | –10.8 | –10.8 | –10.9 |
| 114 | UMHSN00151996 | –9.7 | –10.8 | –10.9 |
| 115 | UMHSN00150154 | –10.7 | –10.8 | –10.9 |
| 116 | UMHSN00163698 | –9.6 | –10.8 | –10.9 |
| 117 | UMHSN00081578 | –10.8 | –10.9 | –10.9 |
| 118 | UMHSN00297776 | –10.9 | –10.9 | –10.9 |
| 119 | UMHSN00338707 | –10.8 | –10.9 | –10.9 |
| 120 | UMHSN00007954 | –10.7 | –10.8 | –10.9 |
| 121 | UMHSN00081712 | –9.7 | –10.8 | –10.8 |
| 122 | UMHSN00016061 | –11.0 | –10.8 | –10.8 |
| 123 | UMHSN00018045 | –9.4 | –10.6 | –10.8 |
| 124 | UMHSN00303503 | –10.8 | –10.8 | –10.8 |
| 125 | UMHSN00008441 | –10.8 | –10.8 | –10.8 |
| 126 | UMHSN00108809 | –10.5 | –10.8 | –10.8 |
| 127 | UMHSN00151984 | –10.4 | –10.7 | –10.8 |
| 128 | UMHSN00311964 | –9.7 | –10.8 | –10.8 |
| 129 | UMHSN00059472 | –10.2 | –10.8 | –10.8 |
| 130 | UMHSN00151328 | –10.3 | –10.8 | –10.8 |
| 131 | UMHSN00272837 | –10.8 | –10.8 | –10.8 |
| 132 | UMHSN00298864 | –10.8 | –10.8 | –10.8 |
| 133 | UMHSN00348298 | –10.8 | –10.8 | –10.8 |
| 134 | UMHSN00004205 | –10.8 | –10.8 | –10.8 |
| 135 | UMHSN00004639 | –10.7 | –10.8 | –10.8 |
| 136 | UMHSN00005544 | –9.6 | –10.8 | –10.8 |
| 137 | UMHSN00009039 | –10.3 | –11.2 | –10.8 |
| 138 | UMHSN00014834 | –11.0 | –11.0 | –10.8 |
| 139 | UMHSN00063515 | –10.4 | –11.0 | –10.8 |
| 140 | UMHSN00081691 | –10.4 | –11.1 | –10.8 |
| 141 | UMHSN00084550 | –11.2 | –11.3 | –10.8 |
| 142 | UMHSN00093977 | –10.1 | –10.6 | –10.8 |
| 143 | UMHSN00106227 | –10.8 | –10.9 | –10.8 |
| 144 | UMHSN00119051 | –10.6 | –11.0 | –10.8 |
| 145 | UMHSN00151638 | –10.3 | –10.9 | –10.8 |
| 146 | UMHSN00249560 | –11.3 | –11.3 | –10.8 |
| 147 | UMHSN00004200 | –10.8 | –10.8 | –10.8 |
| 148 | UMHSN00008338 | –10.8 | –10.8 | –10.8 |
| 149 | UMHSN00011744 | –10.3 | –10.8 | –10.8 |
| 150 | UMHSN00011773 | –10.6 | –10.8 | –10.8 |
| 151 | UMHSN00014784 | –10.7 | –10.8 | –10.8 |
| 152 | UMHSN00081754 | –10.7 | –10.8 | –10.8 |
| 153 | UMHSN00082803 | –10.8 | –10.8 | –10.8 |
| 154 | UMHSN00129049 | –10.4 | –10.8 | –10.8 |
| 155 | UMHSN00008406 | –10.7 | –10.8 | –10.8 |
| 156 | UMHSN00151994 | –10.1 | –10.9 | –10.9 |
| 157 | UMHSN00153504 | –10.8 | –10.9 | –10.9 |
| 153 | UMHSN00234079 | –9.8 | –11.1 | –10.9 |
| 154 | UMHSN00274123 | –11.0 | –11.1 | –10.9 |
| 155 | UMHSN00294698 | –10.8 | –10.9 | –10.9 |

| No. | SuperNatural II Code | Docking Score (kcal/mol) | | |
| --- | --- | --- | --- | --- |
|  |  | Std.^b^ | Mod.^c^ | Exp.^d^ |
| 156 | UMHSN00014909 | –10.8 | –10.8 | –10.8 |
| 157 | UMHSN00054815 | –10.4 | –10.7 | –10.8 |
| 158 | UMHSN00253790 | –10.7 | –10.8 | –10.8 |
| 159 | UMHSN00336301 | –9.7 | –10.8 | –10.8 |
| 160 | UMHSN00352446 | –10.8 | –10.8 | –10.8 |
| 161 | UMHSN00008384 | –10.6 | –10.8 | –10.8 |
| 162 | UMHSN00115247 | –10.5 | –10.6 | –10.8 |
| 163 | UMHSN00323517 | –10.8 | –10.8 | –10.8 |
| 164 | UMHSN00330061 | –10.8 | –10.8 | –10.8 |
| 165 | UMHSN00008429 | –10.7 | –10.8 | –10.8 |
| 166 | UMHSN00008443 | –10.8 | –10.8 | –10.8 |
| 167 | UMHSN00014899 | –10.7 | –10.8 | –10.8 |
| 168 | UMHSN00080671 | –10.6 | –10.8 | –10.8 |
| 169 | UMHSN00335968 | –10.7 | –10.8 | –10.8 |
| 170 | UMHSN00008157 | –10.5 | –10.6 | –10.8 |
| 171 | UMHSN00009723 | –10.3 | –10.8 | –10.8 |
| 172 | UMHSN00053252 | –9.6 | –10.8 | –10.8 |
| 173 | UMHSN00063180 | –9.5 | –10.7 | –10.8 |
| 174 | UMHSN00081550 | –10.4 | –10.6 | –10.8 |
| 175 | UMHSN00081783 | –10.5 | –10.7 | –10.8 |
| 176 | UMHSN00115760 | –10.7 | –10.8 | –10.8 |
| 177 | UMHSN00009829 | –9.6 | –10.7 | –10.7 |
| 178 | UMHSN00052398 | –10.2 | –10.7 | –10.7 |
| 179 | UMHSN00081740 | –10.7 | –10.7 | –10.7 |
| 180 | UMHSN00135413 | –10.7 | –10.8 | –10.7 |
| 181 | UMHSN00004204 | –10.8 | –10.7 | –10.7 |
| 182 | UMHSN00005427 | –10.6 | –10.7 | –10.7 |
| 183 | UMHSN00008792 | –10.5 | –10.7 | –10.7 |
| 184 | UMHSN00063317 | –10.7 | –10.7 | –10.7 |
| 185 | UMHSN00080018 | –10.6 | –10.7 | –10.7 |
| 186 | UMHSN00086813 | –10.6 | –10.7 | –10.7 |
| 187 | UMHSN00099052 | –10.5 | –10.6 | –10.7 |
| 188 | UMHSN00169956 | –10.7 | –10.7 | –10.7 |
| 189 | UMHSN00005492 | –10.7 | –10.7 | –10.7 |
| 190 | UMHSN00081763 | –10.7 | –10.7 | –10.7 |
| 191 | UMHSN00088967 | –11.4 | –10.6 | –10.7 |
| 192 | UMHSN00115713 | –10.7 | –10.7 | –10.7 |
| 193 | UMHSN00148369 | –10.4 | –10.6 | –10.7 |
| 194 | UMHSN00333049 | –10.7 | –10.7 | –10.7 |
| 195 | UMHSN00004667 | –10.7 | –10.7 | –10.7 |
| 196 | UMHSN00007947 | –11.7 | –10.7 | –10.7 |
| 197 | UMHSN00009979 | –10.4 | –10.7 | –10.7 |
| 198 | UMHSN00014810 | –10.7 | –10.7 | –10.7 |
| 199 | UMHSN00082600 | –9.5 | –10.9 | –10.7 |
| 200 | UMHSN00094477 | –10.2 | –10.6 | –10.7 |
| 201 | UMHSN00150295 | –9.7 | –10.7 | –10.7 |
| 202 | UMHSN00362600 | –10.2 | –10.7 | –10.7 |
| 203 | UMHSN00009895 | –9.8 | –10.7 | –10.7 |
| 204 | UMHSN00081048 | –10.4 | –10.7 | –10.7 |
| 205 | UMHSN00081544 | –10.2 | –10.7 | –10.7 |
| 206 | UMHSN00081681 | –10.8 | –11.1 | –10.7 |
| 207 | UMHSN00081816 | –10.7 | –10.7 | –10.7 |
| 208 | UMHSN00151329 | –10.0 | –10.8 | –10.7 |
| 209 | UMHSN00247576 | –10.7 | –10.7 | –10.7 |
| 210 | UMHSN00365861 | –10.7 | –10.7 | –10.7 |
| 211 | UMHSN00004206 | –10.7 | –10.7 | –10.7 |
| 212 | UMHSN00008597 | –10.5 | –10.7 | –10.7 |

| No. | SuperNatural II Code | Docking Score (kcal/mol) | | |
| --- | --- | --- | --- | --- |
|  |  | Std.^b^ | Mod.^c^ | Exp.^d^ |
| 213 | UMHSN00009187 | –10.7 | –10.7 | –10.7 |
| 214 | UMHSN00014892 | –10.7 | –10.7 | –10.7 |
| 215 | UMHSN00091498 | –10.6 | –10.7 | –10.7 |
| 216 | UMHSN00168542 | –10.6 | –10.6 | –10.7 |
| 217 | UMHSN00302589 | –9.9 | –10.7 | –10.7 |
| 218 | UMHSN00012291 | –9.9 | –10.7 | –10.7 |
| 219 | UMHSN00016053 | –10.6 | –10.7 | –10.7 |
| 220 | UMHSN00062722 | –9.5 | –10.5 | –10.7 |
| 221 | UMHSN00081546 | –10.6 | –10.7 | –10.7 |
| 222 | UMHSN00081699 | –10.0 | –10.5 | –10.7 |
| 223 | UMHSN00009725 | –10.8 | –10.7 | –10.7 |
| 224 | UMHSN00024180 | –9.4 | –10.7 | –10.7 |
| 225 | UMHSN00092866 | –10.5 | –10.7 | –10.7 |
| 226 | UMHSN00130521 | –10.4 | –10.7 | –10.7 |
| 227 | UMHSN00318850 | –10.6 | –10.7 | –10.7 |
| 228 | UMHSN00336354 | –9.8 | –10.7 | –10.7 |
| 229 | UMHSN00383584 | –10.7 | –10.7 | –10.7 |
| 230 | UMHSN00060276 | –9.9 | –10.6 | –10.7 |
| 231 | UMHSN00227821 | –10.5 | –10.7 | –10.7 |
| 232 | UMHSN00263341 | –10.7 | –10.7 | –10.7 |
| 233 | UMHSN00366902 | –10.7 | –10.7 | –10.7 |
| 234 | UMHSN00008335 | –10.7 | –10.7 | –10.7 |
| 235 | UMHSN00009841 | –10.5 | –10.7 | –10.7 |
| 236 | UMHSN00010013 | –10.2 | –10.6 | –10.7 |
| 237 | UMHSN00014025 | –10.5 | –10.6 | –10.7 |
| 238 | UMHSN00080976 | –10.4 | –10.6 | –10.7 |
| 239 | UMHSN00278326 | –10.6 | –10.8 | –10.7 |
| 240 | UMHSN00014775 | –10.9 | –11.0 | –10.6 |
| 241 | UMHSN00053443 | –10.3 | –10.6 | –10.6 |
| 242 | UMHSN00089817 | –10.4 | –10.6 | –10.6 |
| 243 | UMHSN00260534 | –10.6 | –10.6 | –10.6 |
| 244 | UMHSN00264160 | –10.5 | –10.6 | –10.6 |
| 245 | UMHSN00310526 | –10.7 | –10.7 | –10.6 |
| 246 | UMHSN00005177 | –9.4 | –10.6 | –10.6 |
| 247 | UMHSN00009049 | –10.5 | –10.6 | –10.6 |
| 248 | UMHSN00057413 | –10.6 | –10.6 | –10.6 |
| 249 | UMHSN00082747 | –10.6 | –10.6 | –10.6 |
| 250 | UMHSN00087820 | –10.6 | –10.6 | –10.6 |
| 251 | UMHSN00112571 | –10.2 | –10.6 | –10.6 |
| 252 | UMHSN00269911 | –10.6 | –10.6 | –10.6 |
| 253 | UMHSN00338048 | –10.6 | –10.6 | –10.6 |
| 254 | UMHSN00382692 | –10.5 | –10.6 | –10.6 |
| 255 | UMHSN00004207 | –10.6 | –10.6 | –10.6 |
| 256 | UMHSN00008556 | –10.5 | –10.6 | –10.6 |
| 257 | UMHSN00019643 | –10.6 | –10.6 | –10.6 |
| 258 | UMHSN00057073 | –10.6 | –10.6 | –10.6 |
| 259 | UMHSN00062775 | –10.0 | –10.6 | –10.6 |
| 260 | UMHSN00150153 | –10.0 | –10.6 | –10.6 |
| 261 | UMHSN00236730 | –10.6 | –10.6 | –10.6 |
| 262 | UMHSN00328645 | –10.6 | –10.6 | –10.6 |
| 263 | UMHSN00352569 | –9.9 | –10.6 | –10.6 |
| 264 | UMHSN00011706 | –10.6 | –10.6 | –10.6 |
| 265 | UMHSN00153950 | –10.0 | –10.7 | –10.6 |
| 266 | UMHSN00244941 | –10.6 | –10.6 | –10.6 |
| 267 | UMHSN00004203 | –10.6 | –10.6 | –10.6 |
| 268 | UMHSN00005184 | –10.6 | –10.6 | –10.6 |
| 269 | UMHSN00010230 | –10.6 | –10.6 | –10.6 |

| No. | SuperNatural II Code | Docking Score (kcal/mol) | | |
| --- | --- | --- | --- | --- |
|  |  | Std.^b^ | Mod.^c^ | Exp.^d^ |
| 270 | UMHSN00059350 | –10.6 | –10.6 | –10.6 |
| 271 | UMHSN00059532 | –10.1 | –10.6 | –10.6 |
| 272 | UMHSN00062729 | –9.5 | –10.7 | –10.6 |
| 273 | UMHSN00078033 | –10.4 | –10.6 | –10.6 |
| 274 | UMHSN00083478 | –9.9 | –10.6 | –10.6 |
| 275 | UMHSN00084548 | –10.5 | –10.6 | –10.6 |
| 276 | UMHSN00330614 | –9.7 | –10.6 | –10.6 |
| 277 | UMHSN00342018 | –10.5 | –10.7 | –10.6 |
| 278 | UMHSN00013285 | –9.5 | –10.6 | –10.6 |
| 279 | UMHSN00014022 | –10.6 | –10.6 | –10.6 |
| 280 | UMHSN00014824 | –10.6 | –10.6 | –10.6 |
| 281 | UMHSN00098733 | –10.5 | –10.6 | –10.6 |
| 282 | UMHSN00105115 | –10.2 | –10.6 | –10.6 |
| 283 | UMHSN00329053 | –10.6 | –10.6 | –10.6 |
| 284 | UMHSN00053405 | –10.4 | –10.6 | –10.6 |
| 285 | UMHSN00062422 | –10.3 | –10.6 | –10.6 |
| 286 | UMHSN00091401 | –10.5 | –10.6 | –10.6 |
| 287 | UMHSN00148371 | –10.3 | –10.5 | –10.6 |
| 288 | UMHSN00169963 | –10.8 | –10.6 | –10.6 |
| 289 | UMHSN00170518 | –10.3 | –10.5 | –10.6 |
| 290 | UMHSN00236373 | –10.5 | –10.6 | –10.6 |
| 291 | UMHSN00308859 | –10.6 | –10.6 | –10.6 |
| 292 | UMHSN00342832 | –10.5 | –10.6 | –10.6 |
| 293 | UMHSN00008382 | –11.1 | –10.6 | –10.6 |
| 294 | UMHSN00008769 | –10.5 | –10.6 | –10.6 |
| 295 | UMHSN00016037 | –9.9 | –10.6 | –10.6 |
| 296 | UMHSN00024014 | –10.5 | –10.6 | –10.6 |
| 297 | UMHSN00054768 | –10.5 | –10.6 | –10.6 |
| 298 | UMHSN00081558 | –10.4 | –10.5 | –10.6 |
| 299 | UMHSN00151590 | –9.9 | –10.5 | –10.6 |
| 300 | UMHSN00361740 | –10.6 | –10.6 | –10.6 |
| 301 | UMHSN00057168 | –10.5 | –10.6 | –10.6 |
| 302 | UMHSN00081096 | –10.6 | –10.6 | –10.6 |
| 303 | UMHSN00234454 | –9.8 | –10.6 | –10.6 |
| 304 | UMHSN00324042 | –10.6 | –10.6 | –10.6 |
| 305 | UMHSN00004199 | –10.6 | –10.6 | –10.6 |
| 306 | UMHSN00080062 | –10.3 | –10.5 | –10.6 |
| 307 | UMHSN00080972 | –9.5 | –10.6 | –10.6 |
| 308 | UMHSN00271512 | –10.5 | –10.6 | –10.6 |
| 309 | UMHSN00291822 | –10.6 | –10.6 | –10.6 |
| 310 | UMHSN00342220 | –10.5 | –10.5 | –10.6 |
| 311 | UMHSN00354461 | –10.5 | –10.6 | –10.6 |
| 312 | UMHSN00013286 | –9.4 | –10.6 | –10.5 |
| 313 | UMHSN00096723 | –9.6 | –10.5 | –10.5 |
| 314 | UMHSN00150297 | –10.5 | –10.5 | –10.5 |
| 315 | UMHSN00294502 | –10.5 | –10.6 | –10.5 |
| 316 | UMHSN00305204 | –10.5 | –10.6 | –10.5 |
| 317 | UMHSN00009939 | –10.4 | –10.5 | –10.5 |
| 318 | UMHSN00010257 | –10.3 | –10.6 | –10.5 |
| 319 | UMHSN00011723 | –10.5 | –10.5 | –10.5 |
| 320 | UMHSN00054061 | –10.5 | –10.5 | –10.5 |
| 321 | UMHSN00054437 | –10.1 | –11.0 | –10.5 |
| 322 | UMHSN00152286 | –10.3 | –10.5 | –10.5 |
| 323 | UMHSN00008439 | –10.5 | –10.5 | –10.5 |
| 324 | UMHSN00081911 | –10.3 | –10.5 | –10.5 |
| 325 | UMHSN00084341 | –10.4 | –10.5 | –10.5 |
| 326 | UMHSN00162447 | –10.5 | –10.5 | –10.5 |

| No. | SuperNatural II Code | Docking Score (kcal/mol) | | |
| --- | --- | --- | --- | --- |
|  |  | Std.^b^ | Mod.^c^ | Exp.^d^ |
| 327 | UMHSN00227072 | –10.5 | –10.5 | –10.5 |
| 328 | UMHSN00332256 | –10.5 | –10.5 | –10.5 |
| 329 | UMHSN00010271 | –10.5 | –10.5 | –10.5 |
| 330 | UMHSN00080923 | –10.4 | –10.5 | –10.5 |
| 331 | UMHSN00150281 | –10.4 | –10.5 | –10.5 |
| 332 | UMHSN00062283 | –10.3 | –10.5 | –10.5 |
| 333 | UMHSN00081092 | –10.5 | –10.5 | –10.5 |
| 334 | UMHSN00095412 | –10.4 | –10.5 | –10.5 |
| 335 | UMHSN00009786 | –9.7 | –10.5 | –10.5 |
| 336 | UMHSN00089693 | –10.5 | –10.5 | –10.5 |
| 337 | UMHSN00261116 | –10.5 | –10.5 | –10.5 |
| 338 | UMHSN00009824 | –10.5 | –10.5 | –10.5 |
| 339 | UMHSN00055512 | –9.6 | –10.5 | –10.5 |
| 340 | UMHSN00235196 | –10.5 | –11.0 | –10.5 |
| 341 | UMHSN00262800 | –10.2 | –10.5 | –10.5 |
| 342 | UMHSN00061044 | –10.5 | –11.1 | –10.5 |
| 343 | UMHSN00089397 | –10.5 | –10.5 | –10.5 |
| 344 | UMHSN00014774 | –10.8 | –10.8 | –10.5 |
| 345 | UMHSN00288287 | –10.6 | –10.7 | –10.4 |
| 346 | UMHSN00341811 | –10.4 | –10.7 | –10.4 |
| 347 | UMHSN00007058 | –10.3 | –10.5 | –10.4 |
| 348 | UMHSN00008214 | –10.5 | –10.6 | –10.4 |
| 349 | UMHSN00317979 | –10.4 | –10.5 | –10.4 |
| 350 | UMHSN00025039 | –10.0 | –10.6 | –10.4 |
| 351 | UMHSN00004201 | –10.7 | –10.9 | –10.3 |
| 352 | UMHSN00008071 | –9.6 | –10.7 | –10.3 |
| 353 | UMHSN00005221 | –9.9 | –10.9 | –10.3 |
| 354 | UMHSN00081735 | –11.8 | –12.0 | –10.2 |
| 355 | UMHSN00082732 | –11.1 | –10.6 | –10.2 |
| 356 | UMHSN00008800 | –10.5 | –10.6 | –10.2 |
| 357 | UMHSN00110070 | –9.9 | –10.5 | –10.1 |
| 358 | UMHSN00009059 | –10.5 | –10.6 | –10.0 |
| 359 | UMHSN00005268 | –10.5 | –10.6 | –10.0 |
| 360 | UMHSN00079235 | –10.9 | –11.0 | –10.0 |
| 361 | UMHSN00022500 | –9.4 | –10.6 | –10.0 |
| 362 | UMHSN00082210 | –10.3 | –10.5 | –9.9 |
| 363 | UMHSN00335595 | –9.8 | –10.8 | –9.9 |
| 364 | UMHSN00008854 | –10.4 | –10.6 | –9.8 |
| 365 | UMHSN00078921 | –10.4 | –10.8 | –9.8 |
| 366 | UMHSN00152431 | –10.3 | –10.6 | –9.7 |
| 367 | UMHSN00087888 | –10.5 | –11.3 | –9.7 |
| 368 | UMHSN00007116 | –10.4 | –10.6 | –9.6 |
| 369 | UMHSN00087720 | –10.5 | –10.6 | –9.6 |
| 370 | UMHSN00062547 | –10.4 | –10.8 | –9.6 |
| 371 | UMHSN00078865 | –10.6 | –10.6 | –9.6 |
| 372 | UMHSN00025816 | –10.3 | –10.9 | –9.5 |
| 373 | UMHSN00371674 | –10.4 | –10.6 | –9.3 |
| 374 | UMHSN00030737 | –9.6 | –10.5 | –9.1 |
| 375 | UMHSN00061021 | –10.2 | –10.5 | –9.0 |
| 376 | UMHSN00060190 | –10.6 | –10.9 | –8.9 |

^a^ Data ranked based on the expensive docking calculations.

^b^ Std. refers to standard docking calculations.

^c^ Mod. refers to moderate docking calculations.

^d^ Exp. refers to expensive docking calculations.
