# Supplementary material for: SuperNatural inhibitors to reverse multidrug resistance emerged by ABCB1 transporter: Database mining, lipid-mediated molecular dynamics, and pharmacokinetics study
Source: PLoS One. 2023 Jul 26;18(7):e0288919. doi: 10.1371/journal.pone.0288919 (PMC10370898; doi:10.1371/journal.pone.0288919)
Supplement: S3 Table — (DOCX) [file pone.0288919.s004.docx]

### S3 Table. Evaluated standard, moderate, and expensive docking scores and MM-GBSA over 5 ns MD simulations (in kcal/mol) for the top 39 compounds and ZQU within the ABCB1 binding pocket. ^a^

| No. | SuperNatural II Code | Docking Score (kcal/mol) | | | MM-GBSA (kcal/mol) |
| --- | --- | --- | --- | --- | --- |
|  |  | Std.^b^ | Mod.^c^ | Exp.^d^ | 5 ns |
|  | **ZQU** | –8.4 | –8.2 | –8.3 | –49.7 |
| 1 | UMHSN00054684 | –11.4 | –11.5 | –11.5 | –68.9 |
| 2 | UMHSN00009999 | –12.5 | –13.1 | –12.6 | –68.6 |
| 3 | UMHSN00097206 | –12.5 | –11.5 | –12.5 | –67.3 |
| 4 | UMHSN00054807 | –12.5 | –13.1 | –12.6 | –60.8 |
| 5 | UMHSN00008763 | –10.4 | –11.9 | –12.1 | –59.9 |
| 6 | UMHSN00081081 | –10.4 | –10.6 | –12.1 | –59.7 |
| 7 | UMHSN00062899 | –12.1 | –12.4 | –12.4 | –59.3 |
| 8 | UMHSN00380932 | –11.6 | –11.8 | –11.8 | –59.2 |
| 9 | UMHSN00059546 | –10.8 | –11.6 | –11.8 | –58.3 |
| 10 | UMHSN00265972 | –11.5 | –11.5 | –11.5 | –58.0 |
| 11 | UMHSN00004668 | –11.0 | –11.6 | –11.6 | –56.7 |
| 12 | UMHSN00249525 | –11.4 | –11.4 | –11.4 | –56.2 |
| 13 | UMHSN00139755 | –9.7 | –11.4 | –11.4 | –55.4 |
| 14 | UMHSN00066079 | –12.4 | –12.4 | –12.4 | –55.2 |
| 15 | UMHSN00377659 | –11.4 | –11.4 | –11.4 | –55.1 |
| 16 | UMHSN00081079 | –11.7 | –11.7 | –11.7 | –54.6 |
| 17 | UMHSN00080936 | –11.9 | –11.9 | –11.9 | –54.5 |
| 18 | UMHSN00007945 | –10.2 | –11.5 | –11.5 | –54.2 |
| 19 | UMHSN00317150 | –11.8 | –11.8 | –11.8 | –54.0 |
| 20 | UMHSN00081067 | –11.8 | –11.8 | –11.8 | –53.9 |
| 21 | UMHSN00079222 | –11.2 | –11.4 | –11.5 | –53.6 |
| 22 | UMHSN00360652 | –11.5 | –11.5 | –11.5 | –53.0 |
| 23 | UMHSN00081043 | –12.5 | –12.6 | –12.5 | –51.8 |
| 24 | UMHSN00009897 | –11.2 | –11.8 | –11.5 | –50.9 |
| 25 | UMHSN00337215 | –11.8 | –11.8 | –11.8 | –50.1 |
| 26 | UMHSN00080939 | –11.8 | –11.8 | –11.8 | –49.9 |
| 27 | UMHSN00089274 | –11.5 | –11.8 | –11.8 | –49.7 |
| 28 | UMHSN00010807 | –11.3 | –11.4 | –11.4 | –49.4 |
| 29 | UMHSN00058310 | –10.5 | –11.8 | –11.8 | –49.3 |
| 30 | UMHSN00084553 | –11.1 | –11.4 | –11.5 | –49.0 |
| 31 | UMHSN00008381 | –10 | –11.5 | –11.6 | –48.8 |
| 32 | UMHSN00079819 | –10.3 | –11 | –11.1 | –48.6 |
| 33 | UMHSN00050809 | –9.9 | –11.6 | –11.3 | –48.3 |
| 34 | UMHSN00302140 | –12.1 | –12.1 | –12.1 | –47.4 |
| 35 | UMHSN00260998 | –10.9 | –11.5 | –11.5 | –46.8 |
| 36 | UMHSN00009954 | –11.4 | –11.5 | –11.6 | –45.5 |
| 37 | UMHSN00062975 | –11.5 | –11.6 | –11.4 | –45.2 |
| 38 | UMHSN00011720 | –12.7 | –12.8 | –11.9 | –44.2 |
| 39 | UMHSN00427183 | –10.9 | –11.4 | –11.2 | –43.9 |

^a^ Data ranked based on the 5 ns MD simulations.

^b^ Std. refers to standard docking calculations.

^c^ Mod. refers to moderate docking calculations.

^d^ Exp. refers to expensive docking calculations.
