# Supplementary figures and images for: SuperNatural inhibitors to reverse multidrug resistance emerged by ABCB1 transporter: Database mining, lipid-mediated molecular dynamics, and pharmacokinetics study
Source: PLoS One. 2023 Jul 26;18(7):e0288919. doi: 10.1371/journal.pone.0288919 (PMC10370898; doi:10.1371/journal.pone.0288919)

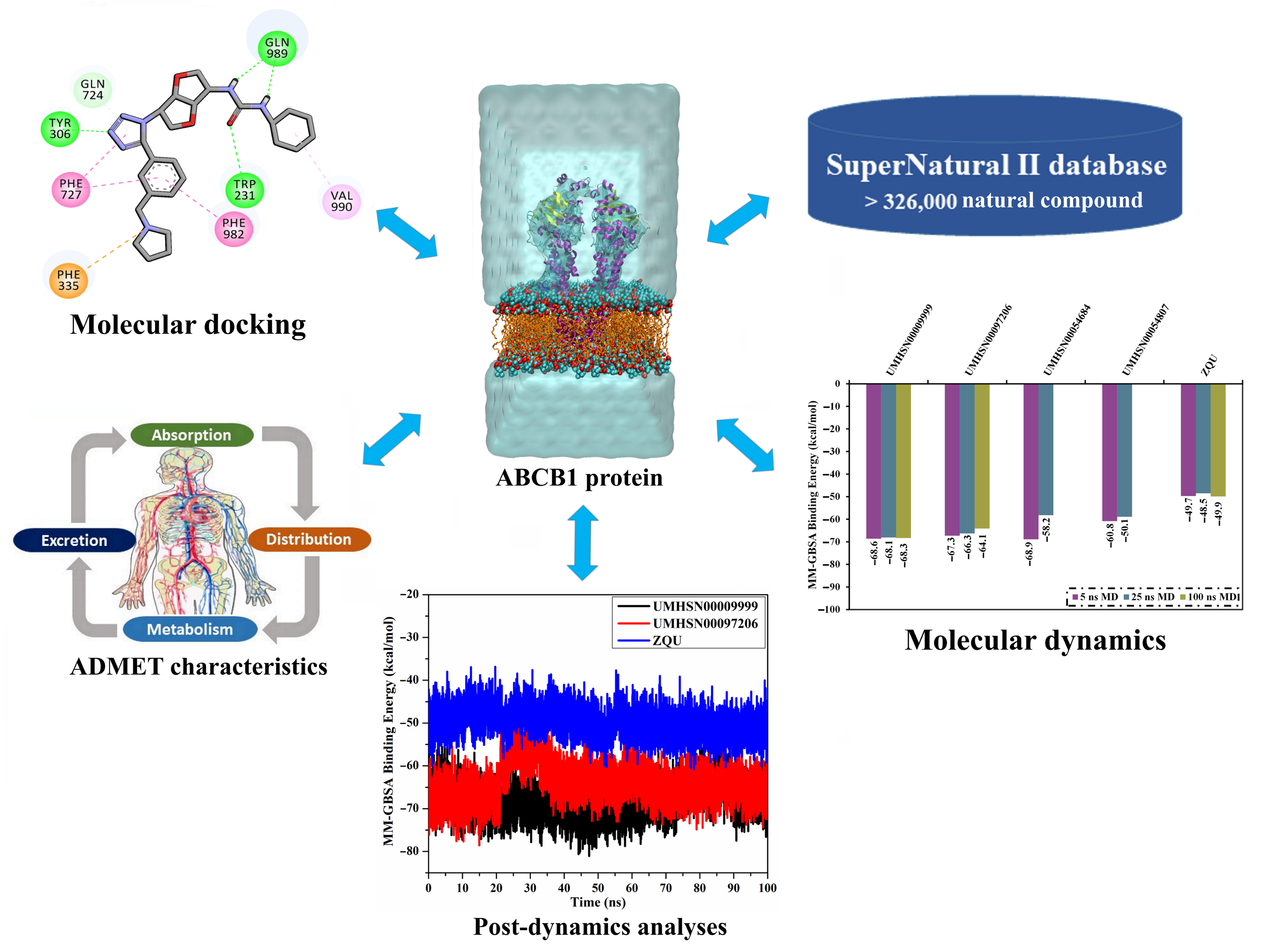

Supplement: S1 Graphical abstract — (TIF) [file pone.0288919.s005.tif]
